# The Clinical Pharmacokinetics and Pharmacodynamics of Glimepiride—A Systematic Review and Meta-Analysis

**DOI:** 10.3390/ph18010122

**Published:** 2025-01-17

**Authors:** Mubara Azhar, Mohammed S. Alasmari, Ammara Zamir, Hamid Saeed, Faleh Alqahtani, Tanveer Ahmad, Muhammad Fawad Rasool

**Affiliations:** 1 Department of Pharmacy Practice, Faculty of Pharmacy, Bahauddin Zakariya University, Multan 60800, Pakistan; mubara.azhar121@gmail.com (M.A.); ammarazamir20@gmail.com (A.Z.); 2 Drug and Poisoning Information Center, Security Forces Hospital, Riyadh 11481, Saudi Arabia; 3 Section of Pharmaceutics, University College of Pharmacy, Allama Iqbal Campus, University of Punjab, Lahore 54000, Pakistan; hamid.pharmacy@pu.edu.pk; 4 Department of Pharmacology and Toxicology, College of Pharmacy, King Saud University, Riyadh 11451, Saudi Arabia; 5 Institute for Advanced Biosciences (IAB), Grenoble Alpes University, 38700 La Tronche, France

**Keywords:** glimepiride, pharmacokinetics, pharmacodynamics, clearance, systematic review, diabetes

## Abstract

**Background/Objectives:** Glimepiride (GLM), a commonly used sulphonylurea drug for the management of type 2 diabetes mellitus (T2DM), has been the subject of numerous studies exploring its kinetic behaviors. However, a comprehensive evaluation that synthesizes all available pharmacokinetic (PK) data across diverse populations remains limited. This systematic review aims to provide detailed knowledge about the pharmacokinetics (PK), the associated pharmacodynamics (PD), and the drug interactions of GLM, which can be used to assess key parameters and identify factors influencing variability across diverse populations and clinical settings. **Methods:** A systematic search of the peer-reviewed literature was combined using major databases—Google Scholar, PubMed, Cochrane, and ScienceDirect, to identify studies reporting the PK of GLM. Following the data extraction, a meta-analysis using a random effect (RE) model was performed, where feasible, to quantitatively assess the variability of key PK parameters across different studies to create a more robust PK parameter estimate. **Results:** The final screening has yielded 40 articles. The area under the curve (AUC_0-∞_) and the peak concentration (C_max_) rise proportionately with increasing doses, depicting the linear kinetics of GLM. The subjects with genotype CYP2C9 *1/*3 depicted a 4-fold higher (AUC_0-∞_) as compared to that of the CYP2C9 *1/*1 population. Preliminary meta-analysis results indicated significant variability in (AUC_0-∞_) and C_max_ values among different studies. Heterogeneity across studies was high, warranting the use of RE models. **Conclusions:** The findings of this review would be helpful in the development and evaluation of PK models that may aid in suggesting individualized dosing.

## 1. Introduction

Glimepiride (GLM) is an antidiabetic drug belonging to the sulphonylurea pharmacological class (SUs) [1]. Its clinical use was initially reported in Sweden in 1995 [2], followed by the subsequent approval from the U.S. Food and Drug Administration (FDA) [3]. This medication is exclusively available in oral dosage form, with doses ranging from 1 to 4 mg [3,4]. The antidiabetic effect of GLM is exerted by binding to the ATP-dependent potassium (K^+^) channels in the pancreatic beta cells [1,5], leading to depolarization and the opening of calcium channels, which subsequently triggers insulin release [6]. GLM is widely utilized in the management of type 2 diabetes mellitus (T2DM) as an adjunct to diet and exercise to improve glycemic control [7].

The Biopharmaceutics Classification System (BCS) categorizes GLM in class II, depicting a low solubility of less than 4 µg/mL in water [8,9] and high permeation ability. It has a rapid and complete absorption with a bioavailability of approximately 100% [3]. The GLM binds to human serum albumin with a fraction > 99.5% [10]. The volume of distribution (Vd) for GLM following an intravenous (IV) dose is 8.81 L [4], and its elimination half-life (t_½_) ranges from 5 to 9 h for both single and multiple doses [2,11]. GLM undergoes complete metabolism via hepatic oxidative biotransformation through CYP2C9 isoenzymes, transforming into its active metabolite ‘cyclohexyl hydroxyl derivative’, which later changes into its inactive form ‘carboxyl derivative’. Around 60% of the given dose is excreted in urine [12] with a total body clearance of 47.8 mL/min [4], while the remaining portion is excreted in feces [13].

GLM has the chemical formula C_24_H_34_N_4_O_5_S [14] and a molecular weight of 490.62 g/mol [1]. The melting point is 207 °C [15], and the lipophilicity (LogP) value is 3.5 [16]. It has an acid dissociation constant (pK_a_) value of 4.32 and −3.7 in the strongest acid and base, respectively [12].

GLM is designated in the pregnancy risk category C [14]. The information on its permeation in breast milk is very sparse [17], so it is inadvisable for use in pregnant or nursing women, and children [18]. The most common adverse effect of GLM is hypoglycemia, which in long-standing T2DM leads to neuroglycopenia and a potential hypoglycemic coma [19]. The other possible side effects may include headache and dizziness, while infrequently occurring complications include hyponatremia, leukopenia, thrombocytopenia, and anemia [1]. GLM should be used cautiously in older individuals, those with anorexic or weakened conditions, and people with liver and kidney dysfunction [18]. 

The primary pharmacodynamic (PD) parameters evaluated in diabetes management include fasting plasma glucose (FPG), glycosylated hemoglobin (HbA1c levels), and 2 h post-prandial plasma glucose (2hr-PPG) levels. Additional PD parameters such as fasting C-peptide levels, serum insulin, changes in body weight, and insulin dosage are also considered in therapeutic assessments [18]. 

Despite the increasing number of studies evaluating the PK of glimepiride, individual studies often report varying results due to differences in study design, route of administration, sample size, or population characteristics. By pooling PK parameter data, the limitations of smaller sample sizes in individual PK studies can be overcome [20]. A meta-analysis allows for the systematic pooling of data, thus offering a more precise estimate of the PK parameter across multiple studies. Moreover, meta-analysis allows for an understanding of the extent of variability, which is crucial for evaluating how consistent or inconsistent a PK parameter is across studies. It also helps in identifying sources of heterogeneity that may affect clinical decision-making, such as dose optimization. This approach enables a more robust assessment of PK outcomes, contributing to informed decision-making in clinical practice and future research.

Online tools such as pkCSM and SwissADME are being used to interpret the PK characteristics of the drug, specifically illustrating its absorption, distribution, metabolism, and excretion. Whilst our manuscript contains data from real-world studies conducted among various types of populations, it contains information on the PK of GLM in healthy populations and diseased groups like renal patients, morbidly obese patients, etc. Moreover, it has also described its PK among the subgroups exhibiting genotypic polymorphism. Moreover, our manuscript also incorporated studies of DDIs, which would assist healthcare workers in understanding how the concomitant use of drugs alters its peculiar PK.

Therefore, in addition to the systematic review that was conducted to comprehensively evaluate the available PK and PD data, a meta-analysis was performed to quantitatively analyze the PK parameters across multiple studies. This additional quantitative approach was conducted to assess and understand the extent of the variability of PK parameters across different studies and populations. The previously reported reviews on the PK of GLM have been published on the impact of genetic polymorphism [21] and its treatment and management [3,18], and others outlined its clinical implications [4,22]. Some systematic reviews have explored the safety and efficacy of GLM monotherapy in children and adolescents and as a combination with metformin for managing T2DM [23,24], with one review addressing its use in elderly patients [25]. Given the absence of any previous systematic review or meta-analysis that comprehensively addresses all aspects of the PK and PD of GLM, this study was conducted to fill this gap in the knowledge.

## 2. Results

### 2.1. Results for the Literature Search

**Figure 1:**
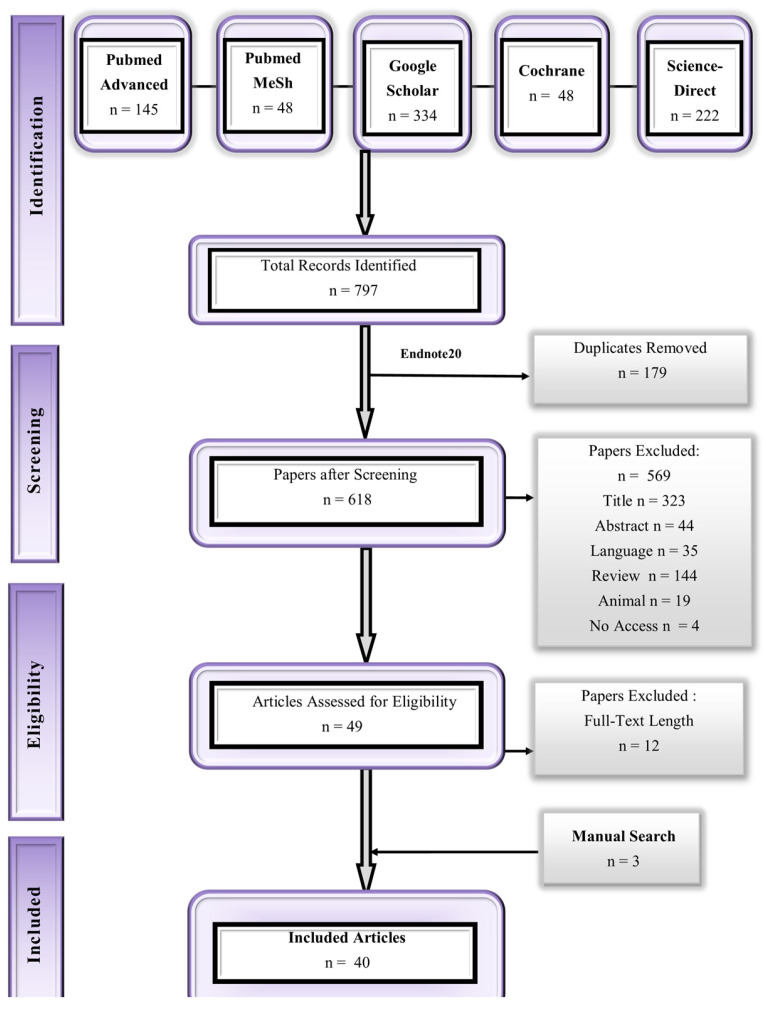
PRISMA flow diagram.

A comprehensive database search resulted in the identification of 797 results. The duplicates were identified and excluded. Out of the remaining 618 studies, 40 were selected in the final review. The screening details are given in the Preferred Reporting Items for Systematic Reviews and Meta-Analysis (PRISMA) flow diagram in Figure 1.

### 2.2. Characteristics of Included Studies

The demographic characteristics for the included articles are shown in Table 1. The PK and associated PD data and drug interactions were separately assessed and recorded. The reported methods of quantification for GLM were also included. To obtain serum glucose, the major parameters were maximum glucose (G_max_), the area under the glucose–time curve (AUGC), the decremental area under the curve (AUC), the maximum increase, as well as the maximum decrease. Moreover, the maximum effect (E_max_), the area under the effect–time curve (AUEC), the incremental AUC, and the maximum increase were used to ascertain the serum insulin.

### 2.3. Quality of the Included Studies

The JADAD, Critical Appraisal Skills Programme (CASP), and Critical Appraisal Clinical Pharmacokinetic (CACPK) scoring were utilized to assess the quality of the retrieved articles. According to JADAD scoring, 16 studies discussed non-randomization. The twenty-four studies were randomized, out of which eighteen were found with moderate and six with low quality. Similarly, thirty-four articles were characterized as high quality, and six studies were rated as being of moderate quality according to CASP criteria. The CACPK indicated that four articles had low, four had moderate, and thirty-two articles had high quality. The risk of bias through the Cochrane Collaboration Tool (CCT) determined that eight studies had a low, twenty-five had a moderate, and seven had a high risk of bias. The questions and details for the scoring of the articles are demonstrated in the Supplementary Information Tables S1–S4.

### 2.4. Clinical Pharmacokinetics of Healthy Populations

#### 2.4.1. Data Following Oral Route of Administration

A total of 24 studies have presented data following the oral route of administration. The comparison of two fixed-dose combinations (FDC) in a clinical study has reported that the area under the curve (AUC)_0-∞_ was found to be 700.6 ng h/mL and 656.0 ng h/mL for the test and reference GLM, respectively [48]. In another study, the administration of 2 mg and 4 mg of GLM resulted in a renal clearance (CL_ʀ)_ of 7.67 mL/min and 32.8 mL/min, correspondingly [32,55]. After administering 2 mg of GLM in two studies, the maximum concentration (C_max_) was recorded as 163.77 ng/mL and 184.7 ng/mL, respectively [33,43]. However, the administration of the doses of GLM ranging from 1 to 6 mg in a study has presented an increase of up to 5-fold in its C_max_ [38]. Additionally, three studies have reported the half-life (t_½_) within the range of 7.37–8.55 h [32,33,37]. 

In a bioequivalence study, the two FDCs of GLM with metformin (MET) reported a C_max_ of 144.0 ng/mL and 143 ng/mL for the test and reference combinations, respectively [34]. Similarly, in another study, the administration of 6 mg of GLM’s test and reference formulations resulted in an AUC_0-∞_ of 4399.0 ng h/mL and 4260.7 ng h/mL, correspondingly [52]. Moreover, in two different bioavailability studies, a comparison of GLM with its FDC tablets of MET [47] and pioglitazone (PIO) [50] produced similar results for the oral clearance (CL/F), with a variation of no more than 1–2%.

The effect of genotypic variations on GLM was investigated in three studies. The administration of 2 mg of GLM in a clinical study has reported a 3-fold increase in the AUC_0-∞_ for genotype CYP2C9 *3/*3 in comparison with CYP2C9 *1/*1 [40]. In another study, the C_max_ for two genotypes, i.e., CYP2C9 *1/*1 and CYP2C9 *1/*3 were recorded as 184.6 ng/mL and 270.5 ng/mL, correspondingly [57]. The other PK parameters are provided in Table 2.

#### 2.4.2. IV Administration

One clinical study depicted a C_max_ of 243 ng/mL after the administration of 1 mg of IV formulation. The remaining PK parameters are mentioned in Table 3 [63].

### 2.5. Clinical Pharmacokinetics of Diseased Populations

#### Oral Administration

Among the included studies, only five were conducted to determine the PK of GLM in the diseased populations. One of the clinical studies showed that C_max_ was 25% lower in obese subjects as compared to normal subjects [13]. In subjects with impaired renal function, the AUC_0-∞_ was decreased up to 54% in patients with CrCL < 20 mL/min in comparison with those having CrCL > 50 mL/min [61]. Another clinical study was conducted among diabetic patients which revealed that CL/F was 22.5 mL/min for CYP2C9 *1/*3 and 63 mL/min for CYP2C9 *1/*1 [51]. The other parameters are summarized in Table 4. 

### 2.6. Studies with Pharmacodynamics of Glimepiride

**Figure 2:**
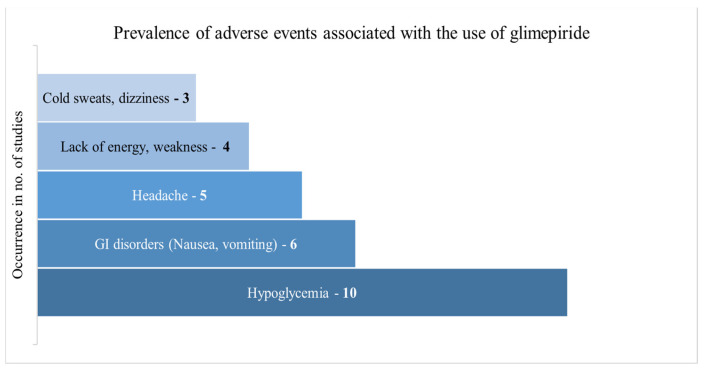
Prevalence of adverse events associated with glimepiride use.

Among the included studies, only seven had discussed the PD of GLM. The main characteristics determined were the HbA1c levels, serum glucose, and serum insulin. The HbA1c value was reported in two studies among the diseased population, with a value ranging between 6.9 and 7.1% [49,61]. Moreover, in a clinical study, E_max_ was found to be 22% higher when GLM was co-administered with evogliptin [26]. Another study presented a maximum increase of ~20% for the serum insulin, whereas other PD parameters were unaffected [58]. The complete information about the PD parameters is shown in Table 5. Several adverse events have been observed with the use of GLM, among them, the major occurring events are depicted in Figure 2.

### 2.7. Studies with the Interactions of Glimepiride

Of the retrieved articles, 11 studies have discussed interactions of GLM with other drugs. In a clinical study, a 33% reduction in AUC_0-∞_ was reported for GLM with rifampicin [60]. The coadministration of colesevelam with GLM reported an increase in the CL/F of the latter, i.e., 58.5 mL/min vs. 72.67 mL/min [35]. After the administration of 3 mg of GLM with 600 mg gemfibrozil, the AUC_0-∞_ was found to be 1.5-fold higher in the former [31]. Similarly, another clinical study reported an elevation in PK parameters (AUC_0-∞_, C_max,_ and t_½_) of up to 20%, following the administration of 0.5 mg of GLM with gemfibrozil [58]. Furthermore, one of the studies depicted a decrease of 23% in C_max_ when dapagliflozin was co-administered with GLM [46]. No significant differences were observed when GLM was concomitantly administered with ertugliflozin and gemigliptin (Table 6) [27,36].

### 2.8. Analysis of Effect Size of PK Parameters

**Figure 3:**
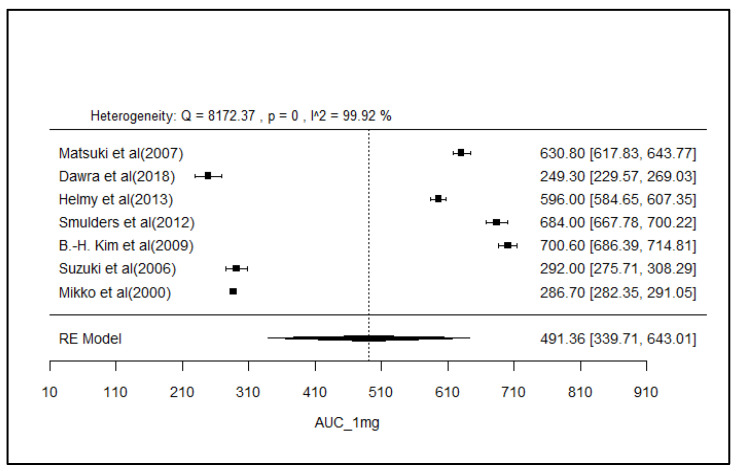
Forest plot of AUC for 1 mg glimepiride across multiple studies. Each study is represented by a square and a horizontal line indicating the 95% confidence interval (CI). The vertical line represents the overall pooled AUC estimate from the random effects model, with the diamond at the bottom showing the pooled AUC value and its CI. Studies with wider CI demonstrate greater variability in AUC values. Heterogeneity among studies is accounted for using the random effects model. In this figure, the pooled AUC for the 1 mg dose is 491.36 (95% CI: 340–643), and an I2 of 99.9% indicates substantial heterogeneity across the studies [27,38,39,48,49,51,60].

**Figure 4:**
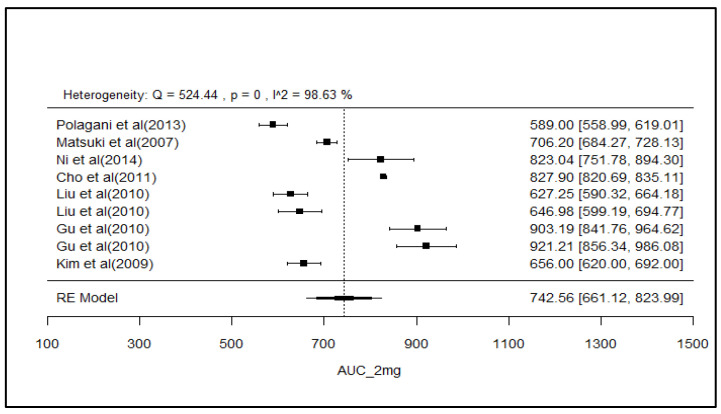
Forest plot of AUC for 2 mg glimepiride across multiple studies. Each study is represented by a square and a horizontal line indicating the 95% confidence interval (CI). The vertical line represents the overall pooled AUC estimate from the random effects model, with the diamond at the bottom showing the pooled AUC value and its CI. In this figure, the pooled AUC for the 2 mg dose is 742.56 (95% CI: 661–824), and an I^2^ of 98.6% indicates substantial heterogeneity across the studies [33,37,42,45,47,48,49].

**Figure 5:**
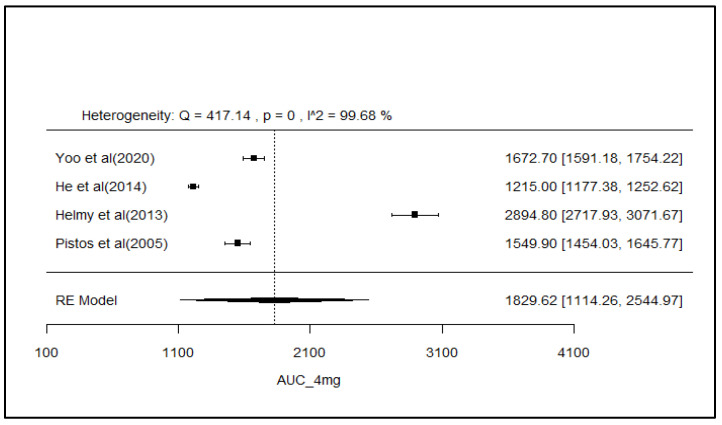
Forest plot of AUC for 4 mg glimepiride across multiple studies. Each study is represented by a square and a horizontal line indicating the 95% confidence interval (CI). The vertical line represents the overall pooled AUC estimate from the random effects model, with the diamond at the bottom showing the pooled AUC value and its CI. In this figure, the pooled AUC for the 4 mg dose is 1830 (95% CI: 1114–2545), and an I^2^ of 99.7% indicates substantial heterogeneity across the studies. [26,35,38,54].

**Figure 6:**
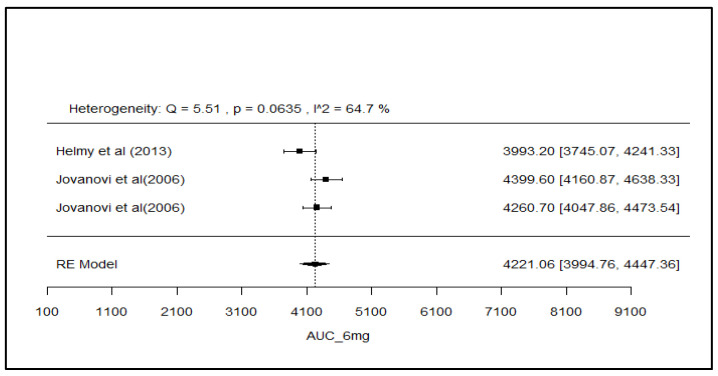
Forest plot of AUC for 6 mg glimepiride across multiple studies. Each study is represented by a square and a horizontal line indicating the 95% confidence interval (CI). The vertical line represents the overall pooled AUC estimate from the random effects model, with the diamond at the bottom showing the pooled AUC value and its CI. In this figure, the pooled AUC for the 6 mg dose is 4221 (95% CI: 3995–4447), and an I^2^ of 64.7% indicates moderate heterogeneity across the studies [38,52].

**Figure 7:**
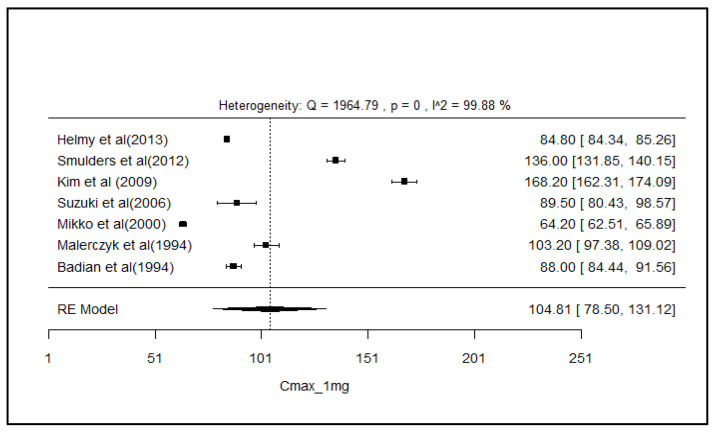
Forest plot of Cmax for 1 mg glimepiride across multiple studies. Each study is represented by a square and a horizontal line indicating the 95% confidence interval (CI). The vertical line represents the overall pooled Cmax estimate from the random effects model, with the diamond at the bottom showing the pooled Cmax value and its CI. In this figure, the pooled Cmax for the 1 mg dose is 104.8 (95% CI: 84.44–91.56), and an I^2^ of 99.88% indicates substantial heterogeneity across the studies [38,39,48,51,60,62,63].

**Figure 8:**
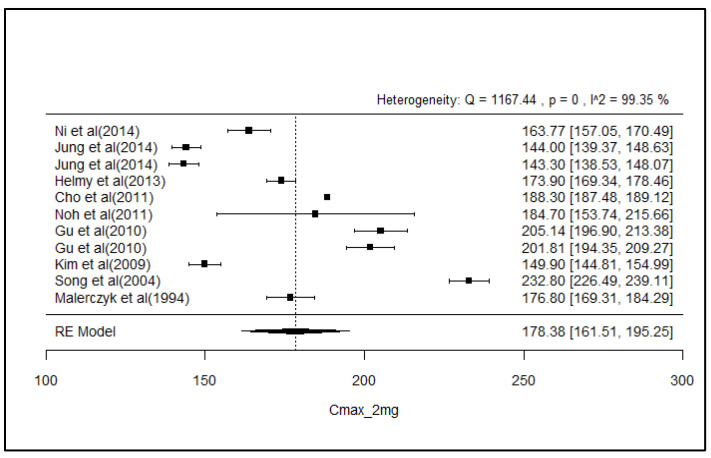
Forest plot of Cmax for 2 mg glimepiride across multiple studies. Each study is represented by a square and a horizontal line indicating the 95% confidence interval (CI). The vertical line represents the overall pooled Cmax estimate from the random effects model, with the diamond at the bottom showing the pooled Cmax value and its CI. In this figure, the pooled Cmax for the 2 mg dose is 178.4 (95% CI: 161.5–195.3), and an I2 of 99.35% indicates substantial heterogeneity across the studies [33,34,38,42,43,47,48,55,62].

**Figure 9:**
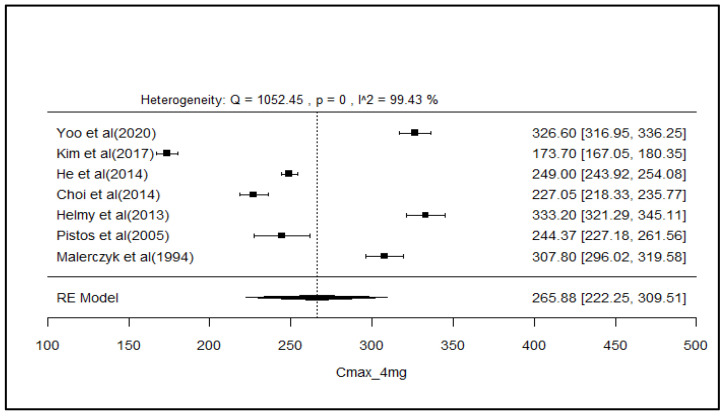
Forest plot of Cmax for 4 mg glimepiride across multiple studies. Each study is represented by a square and a horizontal line indicating the 95% confidence interval (CI). The vertical line represents the overall pooled Cmax estimate from the random effects model, with the diamond at the bottom showing the pooled Cmax value and its CI. In this figure, the pooled Cmax for the 4 mg dose is 265.9 (95% CI: 222.3–309.5), and an I^2^ of 99.4% indicates substantial heterogeneity across the studies [26,29,35,36,38,54,62].

**Figure 10:**
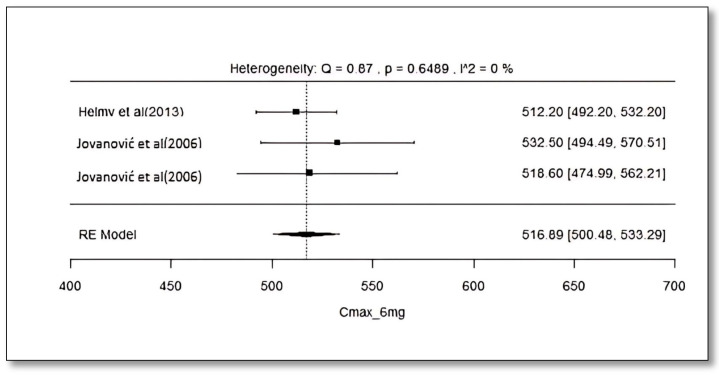
Forest plot of Cmax for 6 mg glimepiride across multiple studies. Each study is represented by a square and a horizontal line indicating the 95% confidence interval (CI). The vertical line represents the overall pooled Cmax estimate from the random effects model, with the diamond at the bottom showing the pooled Cmax value and its CI. In this figure, the pooled Cmax for the 6 mg dose is 516.9 (95% CI: 500.5–533.3), and an I^2^ of 0% indicates low heterogeneity across the studies [38,52].

The pooled AUC and C_max_ after different doses across various studies were estimated using a random effect model (Figure 3, Figure 4, Figure 5, Figure 6, Figure 7, Figure 8, Figure 9 and Figure 10). The pooled AUC for the 1, 2, 4, and 6 mg doses obtained were 491.36 (95% CI: 340–643), 742.56 (95% CI: 661–824), 1830 (95% CI: 1114–2545), and 4221 (95% CI: 3995–4447), respectively. The higher values of I^2^ (>90%) for AUC after 1, 2, and 4 mg depicted a substantial heterogeneity across the studies. Furthermore, the pooled effect sizes for the 1, 2, 4, and 6 mg doses were recorded as 104.8 (95% CI: 84.44–91.56), 178.4 (95% CI: 161.5–195.3), 265.9 (95% CI: 222.3–309.5), and 516.9 (95% CI: 500.5–533.3), conservatively for the C_max_. The higher values of I^2^ (>90%) for C_max_ after 1, 2, and 4 mg also showed substantial heterogeneity across various studies. However, it had been observed that heterogeneity for both parameters decreased with the higher dose of GLM, i.e., 6 mg.

## 3. Discussion

The main objective of this review was to systematically analyze and evaluate the PK and associated PD of GLM by utilizing the data obtained from various studies conducted on healthy and diseased populations. Among the retrieved studies, twenty-four were in the healthy population, five were focused on disease, seven discussed associated PD, and eleven were focused on drug-drug interactions. The C_max_ was higher with the IV formulation which may be due to a higher absorption rate of the GLM since the drug is directly introduced into the main bloodstream [63].

An increase in the C_max_ of the GLM and the recovery of its metabolites was depicted in this study, which affirms that GLM follows dose linearity in its ADME [62]. In a bioequivalence study, the similar values for the PK parameters in a test and reference tablet of GLM depicted that the two brands were bioequivalent to each other [52]. GLM is metabolized by the CYP2C9 enzyme, which has different phenotypes based on its activity; normal metabolizers, i.e., CYP2C9 *1/*1 with normal activity, intermediate metabolizers, i.e., CYP2C9 *1/*3 with the intermediate action of an enzyme, and poor metabolizers, i.e., CYP2C9 *3/*3 having low enzyme activity [65]. In a Korean population, the GLM exhibited variations in AUC_0-∞_ and t_½_ among subjects with intermediate and poor metabolizers, but the total number of participants in these groups was comparatively less than that of the normal metabolizers, thus making it difficult to justify this atypical behavior [40,42]. The bioavailability studies that were performed to compare FDC with the commercial brands of GLM displayed comparable results, ascertaining that they are relatively safe to market [47,50].

The prolonged use of GLM in progressive diseases such as diabetes can significantly affect its PK. As the disease worsens, a reduction in physiological functions is often observed. When there is a decline in kidney function, it leads to the accumulation of its parent drug, which may result in its toxicity. The PK behavior of GLM alters with the decline of physiological functions. Since T2DM is a long-term disease, maintaining compliance with its treatment regimen is often an issue for diabetic patients. A study was conducted to compare the use of GLM as a once-daily (OD) and twice-daily (BID) dose in diabetic patients to check its efficacy and compliance. This study depicted a decrease in exposure to GLM after the BID dose in comparison with OD, which suggests the use of the latter due to its higher compliance in patients with T2DM [49].

In a study conducted among the Japanese population, the genetic polymorphism due to CYP2C9 *3/*3 among diabetic patients depicted a 50% decrease in CL/F resulting in drug accumulation, which suggests its proper monitoring in this instance, as this accumulation may lead to its potential effects. [51]. The factor of obesity in T2DM leads to an abnormal PK profile due to various changes in drug disposition, so the dose and weight may be adjusted to attain normal glycemic values [13]. In T2DM patients with renal impairment, drug accumulation occurred due to the prolonged T_max_ and t_½_ of metabolites, which may result in increased chances of hypoglycemic attacks [61]; therefore, the proper monitoring of blood glucose may be required along with routine checkups for such patients while administering GLM.

When GLM was co-administered with gemfibrozil, there was a rise in the AUC_0-∞_ and C_max_ of the former due to gemfibrozil-mediated CYP2C9 inhibition [31]. Furthermore, the AUC_0-∞_ and C_max_ of GLM were significantly reduced with the concomitant use of bile acid sequestrants such as colesevelam, so these drugs may not be advised together. However, if needed, it is safer to administer GLM a few hours before colesevelam to avoid their potent interaction [35]. Moreover, fluconazole on coadministration with GLM resulted in the prolonged t_½_ of the latter; therefore, to avoid its effects, the dosing frequency of GLM may be reduced [59]. The administration of rifampicin with GLM produced minor effects on PK, but the person may still require monitoring as rifampicin is an inducer of CYP2C9, which plays a major part in the metabolism of GLM [60]. In addition, GLM showed no plausible interactions with ertugliflozin, ipragliflozin, evogliptin, and gemigliptin, which suggested their safe use when administered together [26,27,36,39].

The use of glyburide with GLM did not cause an impact on its PD in the given study, which may owe to the utilization of a very low dose, i.e., 0.5 mg of the latter [57]. Gemfibrozil is considered to have toxic and serious effects in patients using GLM because it is a potent inhibitor of the CYP2C9, but it has displayed minimum effects on its PD [58]. Furthermore, the coadministration of fluconazole and GLM led to a significant increase in GLM plasma levels and exposure due to the inhibition of CYP2C9-related metabolic transformation [66]. GLM has shown better glucose control with the concomitant use of GLM and evogliptin in treating T2DM [26]. Moreover, the administration of GLM as OD and BID regimens is considered safe enough, as their use did not significantly affect the PD and therefore is considered reliable [49].

To derive a more robust estimate of the PK parameters that account for variability across different studies, a meta-analysis was conducted for AUC and C_max_ data obtained from multiple studies to estimate a more precise overall effect size for these parameters. Since different doses of glimepiride were included across the studies, this meta-analysis allowed for an indirect assessment of dose proportionality by examining how a PK parameter scales with dose. Evaluating dose proportionality helps ensure that the drug behaves predictably across different dosing regimens, which is essential for safe and effective dosing guidelines. Therefore, a meta-analysis of pharmacokinetic studies provides valuable pooled estimates of a PK parameter, identifies variability, assesses generalizability, and supports dose optimization.

The pooled AUC and C_max_ were estimated using a random effects model across various studies with different doses (Figure 3, Figure 4, Figure 5, Figure 6, Figure 7, Figure 8, Figure 9 and Figure 10). Dosing proportionality has been verified from this meta-analysis for both AUC and C_max_. Significant heterogeneity, which could not be fully accounted for by the random effects model, was observed, as indicated by the I² statistic for the included studies. Different ethnic groups are more prone to exhibit genotypic variations related to the CYP2C9 enzyme. Additionally, the presence of comorbid conditions, study designs and techniques employed to measure concentrations, and the computation of PK parameters may be considered as these elements contribute to significant heterogeneity. Consequently, there is a need for future studies to place greater emphasis on the approach of addressing these factors. This highlights the need for careful dose adjustment in specific populations and suggests that further studies may be necessary to reduce uncertainty and better understand the factors influencing exposure to GLM and investigate their clinical implications.

Different scenarios are vital in clinical decision-making, in addition to dosage adjustments. First, patients with impaired kidney functions have a greater likelihood of developing hypoglycemia, highlighting the importance of monitoring the renal parameters in addition to blood glucose levels. Second, the potential drug interactions may reduce or enhance the effectiveness of GLM by influencing its metabolism via the CYP2C9 enzyme. Third, the impact of genotypic variations is more common in some populations which affects its efficacy and safety; therefore, tailoring the dosage regimen in such scenarios is crucial for achieving better outcomes.

This paper encompasses all the studies published until 10th October 2023. To date, no such review and meta-analysis is available that covers all the clinical parameters and interactions of GLM in detail. However, there were some restrictions such as less available studies performed among the diseased subgroups. In the case of renal impairment, there was only one study that interpreted the PK of GLM, which may have affected the accuracy of the results. Moreover, the available literature for examining the PK of GLM in high-risk groups is insufficient to facilitate and predict its PK behavior efficiently; therefore, it is recommended to conduct PK studies of GLM in those specific groups of the population. Furthermore, several studies had incorporated very small sample sizes, which may have resulted in the variability of PK characteristics and overall heterogeneity. For better reporting of PK data in future studies, the study design should clearly explain their sampling size and inclusion and exclusion criteria. Furthermore, the information regarding the covariates of the included studies such as age, gender, and weight should be explained explicitly. The method of assay should be mentioned and defined to check its accuracy and specificity. The doses of all the drugs utilized in the clinical study, as well as their dosage form and frequency, may be mentioned to avoid any confusion. Moreover, if there are any dropouts or missing data, it must be described to provide a clear validation. Moreover, some studies did not give any information on the distribution of subjects based on gender. Additionally, graphs were digitally scanned followed by the NCA as PK parameters were not given for them, which may lead to speculations in the final results up to some level.

## 4. Materials and Methods

### 4.1. Study Design for Review

This systematic screening was performed with the help of the Cochrane Handbook guidelines [67], and the Preferred Reporting Items for Systematic Reviews and Meta-Analysis (PRISMA) [68] was utilized for reporting the studies. This review is registered under the International Prospective Register of Systematic Reviews (PROSPERO) with the registry number CRD42024607962.

### 4.2. Search Strategy for the Literature

**Figure 11:**
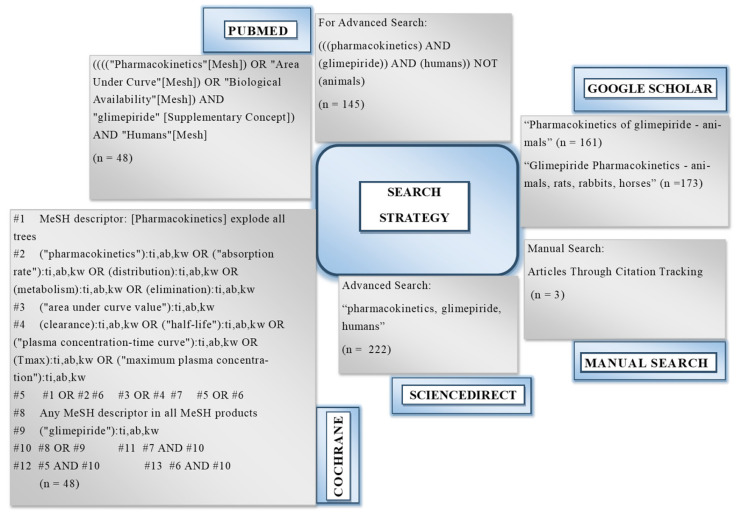
Literature search strategy.

An extensive literature review was performed for the screening of the articles by utilizing relevant keywords for the drug such as ‘pharmacokinetics’, ‘glimepiride’, ‘humans’, etc. This literature search was conducted until 10th October 2023, and various databases were comprehensively reviewed, i.e., Google Scholar, PubMed, Science Direct, and Cochrane. This search strategy is documented in the form of a flowchart as given in Figure 11.

### 4.3. Eligibility Criteria

This review comprised peer-reviewed articles containing data on the concentration–time profiles for oral and IV routes and in the populations including healthy and diseased conditions. Only the articles written in the English language were eligible. The clinical studies were selected irrespective of their year of publication and study design. Moreover, study criteria were established regardless of drug formulation, regimen, and design. Furthermore, studies addressing the drug interactions with GLM were also included in this review to evaluate potential modifications in its absorption, distribution, metabolism, and elimination (ADME).

### 4.4. Procedure for Selection

All articles retrieved from the specified databases were imported to Endnote 20 (version 20.0.1, Clarivate Analytics, Philadelphia, PA, USA), and duplicates were removed by the ‘remove duplicates’ option. After the elimination of duplicate articles, the remaining articles were reviewed by their titles and given abstracts. Any studies that involved animals or had no access were also excluded from the review. Lastly, the articles were thoroughly studied and selected after a full-text reading. The screening details are available in the Supplementary Table S5.

### 4.5. Procedure for Extraction of Data

The demographic details were retrieved from the included studies such as the author’s name, the title of the study, year of publication, gender, age, drug included, dosage form, dosage regimen, frequency, and associated PD data. Moreover, the method of assay was also included along with the demographics to assess the methods being employed in the determination and validation of GLM. In some studies, the PK data were not provided but concentration–time graphs were given, which were then scanned through GetData Graph Digitizer software (version 2.24), and non-compartmental analysis (NCA) was performed through PK Solver which is an add-in program in Microsoft Office Excel (version 2016) to calculate the PK parameters. The units of various PK and PD parameters were unified into similar units for proper interpretation [69].

### 4.6. Assessment of Quality of Included Studies

The quality of articles was assessed using various tools mentioned in the previously reported systematic reviews. Among them, JADAD [70] scores were categorized as high, low, or moderate with the values of more than four, less than three, and three to four, correspondingly. For the CASP tool [66], the articles were considered high quality if their scores exceeded six, moderate if four to six, and low if below four scores, whereas in the case of the Critical Appraisal Clinical Pharmacokinetic CACPK tool [71], it categorized it as a high-quality study if the score was above 13, moderate from 12 to 13, and low if it was below 12. Moreover, the Cochrane Collaboration Tool (CCT) [72] was used to check the risk of bias that classifies studies based on high, moderate, and low risk, if the total scores are below three, between three and four, and >four, respectively.

### 4.7. Summary Measures for Analysis

To obtain a more reliable estimate of PK parameters (AUC and C_max_) and its variability across various studies, a meta-analysis was performed on AUC and C_max_ data from multiple studies to determine a more accurate overall effect size. A random effects model was used to estimate the pooled effect sizes for PK parameters from the included studies. Not all studies in the systematic review were subjected to the meta-analysis. Only studies that reported the necessary quantitative data such as sample size, mean, and variance of PK parameters, were considered for the quantitative analysis. Studies lacking sufficient data for statistical pooling were excluded from meta-analysis but were still discussed qualitatively in the systemic review. The meta-analysis was conducted using the R programming language, employing the “metafor” package for effect size calculations [73]. Heterogeneity between studies was assessed using the I² statistic. The data were presented as means and standard deviations (SDs).

## 5. Conclusions

This review and meta-analysis have compiled all the available clinical studies of GLM conducted in healthy and diseased populations (diabetes, impaired kidney functions, and obesity). Additionally, it has provided data following oral and IV routes. There was a significant alteration observed in its PK in the case of renal impairment due to the accumulation of its active metabolites, which could lead to potential toxicity and adverse events. The genotypic polymorphism occurring due to the CYP2C9 enzyme produces peculiar changes in the PK of GLM, affecting its overall efficacy. This analysis has described and assessed the heterogeneity in AUC and the C_max_ of glimepiride through forest plots and I^2^ statistics. The evaluation of all studies indicated that GLM has a dose-dependent increase in its parameters such as AUC_0-∞_ and C_max_, which has been further verified through meta-analysis. This review has also provided insight into the clinical aspects and drug interactions which may be useful for clinicians to select suitable combinations. Moreover, the data given in this review may be used to develop PK drug models.

## Figures and Tables

**Table 1:** Study characteristics.

Scheme	Age (Years)	Gender	Population	Drug	Dose (mg)	Brand Name	Method of Analysis	Dosage Form	Frequency	Ref.
1	19–45	Male _100%_	HP (Korean)	GLM EVO	4 5	Amaryl^®^, Sanofi-Aventis	LC-MS	Tab	OD	[26]
2	18–55	Male _55%_ Female _44%_	HP (European)	GLM ERT	1 15	N/S	HPLC-MS	Tab	SD	[27]
3	25–45 ^a^	Male _100%_	HP (Egyptian)	GLM	1 ^a^	Amaryl^®^, Hoechst Marion Roussel	HPLC	Tab	OD	[28]
4	20–45 ^b^	Male _100%_	HP (Korean)	GLM ROS	4 20	N/S	HPLC-MS	Tab	OD	[29]
5	25–45 ^c^	Male _100%_	HP (Egyptian)	GLM	1	Amaryl^®^	LC-MS/MS	Tab	SD	[30]
6	18–27	Male _100%_	HP	GLM GMF	3 600	Amaryl^®^, Sanofi-Aventis	UV-Visible Spectroscopy	Tab	SD	[31]
7	N/S	N/S	HP (Chinese)	GLM	2	N/S	UPLC-MS	N/S	OD	[32]
8	19–28	Male _100%_	HP (Chinese)	GLM	2	Amaryl^®^, Sanofi-Aventis	LC-MS	Tab	SD	[33]
9	19–30 ^d^	Male _100%_	HP (Korean)	FDC	2/500	N/S	HPLC-LC/MS/MS	Tab	SD	[34]
10	18–45	Male Female	HP (American)	GLM COL	4	N/S	LC-MS	N/S	SD	[35]
11	20–45	Male _100%_	HP (Korean)	GLM GEMI	4 60	N/S	LC-MS/MS	Tab	SD	[36]
12	N/S	Male _100%_	HP (Indian)	GLM	2	TRIPILL (Cipla Limited	MS	N/S	OD	[37]
13	18–29	Male _100%_	HP (Egyptian)	GLM	1–6	Amaryl^®^, Sanofi-Aventis	HPLC-UV	Tab	SD	[38]
14	18–55	Male _50%_ Female _50%_	HP (Egyptian)	GLM IPRA	1, 2 150	N/S	LC-MS	Tab	SD Multiple- SD	[39]
15	22–29	Male _100%_	HP (Korean)	GLM	2	Amadiem; Dongsung Pharmaceutical Co	LC-MS	Tab	SD	[40]
16	N/S	N/S	HP (Korean)	GLM	1	N/S	LC-MS	N/S	N/S	[41]
17	20–28	Male _100%_	HP (Korean)	GLM	2	Amaryl, Handok/Aventis Pharma	LC-MS/MS	Tab	SD	[42]
18	N/S	N/S	HP (Korean)	GLM	2	N/S	LC-MS	Tab	SD	[43]
19	N/S	N/S	Disease (Korean)	GLM	2	Amaryl, Aventis	HPLC	Tab	N/S	[44]
20	18–26 ^e^	Male _100%_	HP (Japanese)	GLM	2	Dr. Reddy’s Laboratories Ltd.; Amaryl^®^ (Sanofi-Aventis Pharma	LC-MS	Tab	SD	[45]
21	18–45	Male _67%_ Female _33%_	HP (African American or Caucasian)	GLM DAPA	4 20	N/S	LC/MS-MS	Tab	SD	[46]
22	M = 18–26 F = 20–38	Male _50%_ Female _50%_	HP (Korean)	FDC ^d^ GLM, MET	2/500 2, 500	Amaryl^®^-M 2/500, Handok Pharmaceutical Co.; Amaryl^®^ Handok Pharmaceutical Co.	LC/MS-MS	Tab	SD	[47]
23	20–36	Male _100%_	HP (Korean)	FDC ^d, e^	1/500, 2/500 2/500, 1/500	Amaryl^®^-M 1/500, Handok Pharmaceutical Co.; Amaryl^®^-M 2/500, Handok Pharmaceutical Co.	HPLC-MS/MS	Tab	SD	[48]
24	62–65	Male _75%_ Female _25%_	Disease (Japanese)	GLM	1, 2	N/S	LC-MS	N/S	OD/BID	[49]
25	18–55	Male _49%_ Female _51%_	HP	FDC ^f^ GLM	4/30 4	N/S	LC-MS/MS	Tab	SD	[50]
26	35–85	Male Female	Disease (Japanese)	GLM	1	Amaryl, Aventis	N/S	Tab	SD	[51]
27	20–50	Male _62.5%_ Female _37.5%_	HP (Caucasian)	GLM ^e^	6	Amaryl^®^, Aventis Pharma; Remevita	HPLC	Tab	SD	[52]
28	N/S	Male _100%_	HP (Lebanese)	GLM	3	Amaryl^®^, Aventis	HPLC	Tab	SD	[53]
29	25–26	Male Female	HP (Caucasian)	GLM	4	N/S	LC-MS	Tab	SD	[54]
30	23–25	Male _100%_	HP (Asian)	GLM	2	Amaryl^®^, Handok-Aventis	HPLC	Tab	SD	[55]
31	18–70	Male _50%_ Female _50%_	Disease	GLM	8	N/S	HPLC	Tab	SD	[13]
32	N/S	N/S	HP	GLM	3	N/S	LC-MS	Tab	SD	[56]
33	(19–36) (19–27) ^g^	Male _33%_ Female _66%_	HP (Caucasian)	GLM GLY	0.5	Amaryl^®^, Sanofi-Aventis	N/S	Tab	SD	[57]
34	20–26	Male _20%_ Female _80%_	HP	GLM GMF	0.5	Amaryl^®^, Sanofi-Aventis	N/S	Tab	SD	[58]
35	19–27	Male _50%_ Female _50%_	HP	GLM FLC FLV	0.5 200, 400 100	Amaryl^®^, Sanofi-Aventis	N/S	Tab	SD	[59]
36	19–26	Male _50%_ Female _50%_	HP	GLM RIF	1	Amaryl^®^, Sanofi-Aventis	N/S	Tab	SD	[60]
37	(44–70) ^h^ (49–75) ^i^	Male _47%_ Female _53%_	Disease	GLM	1, 2, 3, 4, 6, 8	N/S	N/S	Tab	SD	[61]
38	23–32	Male _100%_	HP (Caucasian)	GLM	1, 2, 4, 8	Amaryl^®^	N/S	Tab	SD	[62]
39	18–40	Male _100%_	HP (Caucasian)	GLM	1	Amaryl^®^	HPLC	Tab, Inj	SD	[63]
40	N/S	N/S	N/S	GLM	3	N/S	HPLC	Tab	SD	[64]

Ref.: references; HP: healthy population; EVO: evogliptin; GLM: glimepiride; LC: liquid chromatography; MS: mass spectroscopy; OD: once daily; Tab: tablet; M: male; F: female; HPLC: high-performance liquid chromatography; ROS: rosuvastatin; ERT: ertugliflozin; SD: single dose; GMF: gemfibrozil; UPLC: ultra-performance liquid chromatography; N/S: not stated; MET: metformin; COL: colesevelam; GEMI: gemigliptin; IPRA: ipragliflozin; DAPA: dapagliflozin; FDC: fixed-dose combination; OD: once daily; BID: twice a day; BA: bioavailability; BE: bioequivalence; BID: twice a day; PIO: pioglitazone; GLY: glyburide; FLC: fluconazole; FLV: fluvoxamine; RIF: rifampicin; S: single-dose study; M: multiple-dose study; Inj.: injection (a) Group 1: optimized drug, Group 2: pure drug, and Group 3: commercial GLM tab; (b) G: GLM treatment, GR: GLM + ROS treatment, and R: ROS treatment; (c) Group 1: pure drug, Group 2: optimized drug, and Group 3: commercial GLM drug; (d) FDC: fixed-dose combination (GLM/MET); (e) test and reference GLM tab; (f) FDC: fixed-dose combination (GLM/PIO); (g) GLY, GLM; (h) single-dose study; (i) multiple-dose study.

**Table 2:** PK parameters in healthy populations following oral route.

Sr	Ref.	Dose (mg)		(AUC)_0-∞_ (ng.× h/mL)	C_max_ (ng/mL)	T_max_ (h)	t½ (h)	CL/F (mL/min)	CLʀ (mL/min)
1	[28]	1 ^a^		178.988	147.7	4	N/S	N/S	N/S
				2.19	32.47	2.5	N/S	N/S	N/S
				15.967	135.77	2.5	N/S	N/S	N/S
2	[30]	1 ^b^		3.595	21.533	4	4.006	N/S	N/S
				19.791	46.09	2.5	4.911	N/S	N/S
				6.626	135.16	2.5	3.55	N/S	N/S
3	[33]	2		823.04 ± 290.87	163.77 ± 45.73	2.53 ± 0.62	7.37 ± 2.24	N/S	N/S
4	[32]	2		4452.06 ± 539.78	414.83 ± 20.45	N/S	8.22 ± 2.50	N/S	7.67 ± 1
5	[34]	2/500 ^c^		N/S	144.0 ± 49.8	2.2 ± 0.8	3.0 ± 2.0	N/S	N/S
				N/S	143.3 ± 51.3	2.0 ± 0.9	2.9 ± 1.9	N/S	N/S
6	[37]	2		589 ± 75	62.8 ± 7.9	5.33 ± 0.52	8.55 ± 1.87	N/S	N/S
7	[38]	1		596.0 ± 141.9	84.8 ± 3.81	2.4 ± 1.7	8.81 ± 1.1	121.8 ± 29.4	N/S
		2		7.07 ± 1.41	173.9 ± 38.0	2.6 ± 1.2	7.07 ± 1.41	116.9 ± 25.9	N/S
		3		8.54 ± 3.31	254.7 ± 54.5	2.19 ± 0.83	8.54 ± 3.31	114.1 ± 32.2	N/S
		4		2894.8 ± 1105.2	333.2 ± 99.2	2.9 ± 0.9	7.63 ± 1.5	112 ± 44.1	N/S
		6		3993.2 ± 1550.5	512.2 ± 100.2	2.5 ± 0.8	8.75 ± 1.3	123.9 ± 56.7	N/S
8	[40] ^*^	CYP2C9 Genotypes							
		2	*1/*1	892.7	218.5	2.8	2.9	42.4583	N/S
			*1/*3	832.9	161.3	3	2.9	40.0217	N/S
			*3/*3	4371.9	267.8	4	12.3	7.625	N/S
9	[41] ^*^	1 ^d^		881.69	83.56	13.45	0.74	18.9	N/S
				795.7	106.84	13.44	0.53	20.95	N/S
10	[42]	CYP2C9 Genotypes							
		2	Total	827.9 ± 49.9	188.3 ± 9.5	2.6 ± 0.1	6.1 ± 0.3	N/S	N/S
			*1/*1	806.1 ± 48.9	184.6 ± 9.1	2.5 ± 0.1	6.2 ± 0.4	N/S	N/S
			*1/*3	1309.65	270.55	3.25	5.6	N/S	N/S
11	[43] ^*^	2 ^e^	Parent drug	N/S	184.7 ± 91.2	2.8 ± 1.3	4.3 ± 0.5	N/S	N/S
			M 1	N/S	29.3 ± 4.9	3.5 ± 1.3	4.3 ± 0.6	N/S	N/S
			M 2	N/S	4.9 ± 1.9	6.7 ± 1.2	3.8 ± 0.5	N/S	N/S
12	[45]	2 ^f^	Parent drug	627.25 ± 184.61	75.71 ± 21.11	5.76 ± 2.07	4.74 ± 1.66	N/S	N/S
				646.98 ± 238.92	74.05 ± 24.95	4.93 ± 2.81	4.08 ± 1.18	N/S	N/S
			M 1	288.76 ± 63.09	25.13 ± 5.06	7.07 ± 2.45	5.74 ± 1.45	N/S	N/S
				303.46 ± 61.86	24.83 ± 5.13	6.78 ± 2.43	5.05 ± 1.13	N/S	N/S
13	[47]	2/500 ^c^		903.19 ± 250.72	205.14 ± 56.08	1.0 ^1^	6.89 ± 2.46	39.333 ± 10	N/S
		2		921.21 ± 264.77	201.81 ± 50.75	3.0 ^1^	6.39 ± 1.77	38.5 ± 9.33	N/S
14	[48]	1/500 ^c^		700.6 ± 198.6	168.2 ± 54.9	1.75 ^1^ (1.0–4.0) ^2^	8.2 ± 2.5	N/S	N/S
		2/500 ^c^		656.0 ± 201.2	149.9 ± 47.4	2.02 ^1^ (1.0–4.0) ^2^	8.5 ± 2.7	N/S	N/S
15	[50] ^3^*	4/30 ^g^		2363.39	285.29	3.02	13.05	112.85	N/S
		4		2334.51	304.60	2.33	18.07	114.22	N/S
16	[52] ^3^	6 ^d^		4399.6 ± 1491.8	532.5 ± 190.1	2.5 ± 0.5	8.8 ± 3.0	N/S	N/S
				4260.7 ± 1330.0	518.6 ± 218.6	2.5 ± 0.7	8.9 ± 2.5	N/S	N/S
17	[53] ^3^*	3		1301.85	220.58	2.69	5.04	115.22	N/S
18	[54]	4		1549.9 ± 299.54	244.37 ± 71.6	2.9 ± 0.98	4.61 ± 0.83	N/S	N/S
19	[55]	2		N/S	232.8 ± 28.4	2.14 ± 0.38	2.16 ± 0.21	N/S	32.8 ± 7.9
20	[56] *	3		1044.38	210.82	1.56	15.485	143.63	N/S
21	[57]	CYP2C9 Genotype		116.1 ^1^ (60.1–171.1) ^2^	29.9 ^1^ (23.1–51.2) ^2^	1.25 ^1^ (1.0–5.0) ^2^	1.9 ^1^ (1.1–2.5) ^2^	N/S	N/S
		0.5	*1/*1						
			*1/*2	125.1 ^1^ (94.7–170.5) ^2^	31.6 ^1^ (17.5–35.8) ^2^	2.0 ^1^ (1.5–4.0) ^2^	1.9 ^1^ (1.8–3.2) ^2^	N/S	N/S
			*1/*3 or *2/*3	310.1 ^1^ (151.2–313.3) ^2^	37.8 ^1^ (2951.4) ^2^	2.5 ^1^ (1.5–4) ^2^	3 ^1^ (2.5–4) ^2^	N/S	N/S
22	[62] ^3^	1 ^f^ 2 ^f^ 4 ^f^ 8 ^f^	Parent drug	N/S	103.2 ± 34.3	2.3 ± 0.5	1.2 ± 0.5	N/S	55.3 ± 16.3
				N/S	176.8 ± 44.1	2.4 ± 0.5	1.3 ± 0.4	N/S	53.5 ± 15.5
				N/S	307.8 ± 69.4	2.1 ± 0.6	1.5 ± 0.5	N/S	53.6 ± 10.6
				N/S	550.8 ± 151.9	2.8 ± 1.2	1.5 ± 0.4	N/S	65.5 ± 21.1
		1 ^f^ 2 ^f^ 4 ^f^ 8 ^f^	M 1	N/S	24.0 ± 5.7	2.8 ± 0.8	3.1 ± 0.9	N/S	146 ± 72
				N/S	42.1 ± 9.6	2.8 ± 0.5	2.5 ± 0.7	N/S	170 ± 48
				N/S	76.7 ± 19.9	3.3 ± 0.7	3.0 ± 0.8	N/S	166 ± 90
				N/S	135.0 ± 40.4	3.4 ± 1.1	3.2 ± 1.1	N/S	169 ± 47
23	[63] ^3^	1 ^f^		N/S	88 ± 21	2.7 ± 1.4	3.1 ± 1.2	N/S	45 ± 16
				N/S	20 ± 6	3.9 ± 1.9	3.3 ± 1.2	N/S	143 ± 48
24	[64] ^3^	3 ^e^		634.36	348	0.77	1.09	236.46	N/S
				383.12	91.2	1.50	2.39	436.522	N/S
				173.59	35.2	3.01	2.39	864.08	N/S

(AUC)0-∞: area under the curve from zero to infinity; C_max_: Maximum Plasma and Serum Concentration; T_max_: time to reach maximum concentration; t½: half-life; CL/F: oral clearance; CLʀ: renal clearance; Ref.: reference; HP: healthy population; N/S: not stated; M 1: metabolite; M 2: metabolite 2; (a) Group 1: optimized drug, Group 2: pure drug, and Group 3; commercial GLM tab; (b) Group 1: pure drug, Group 2: optimized drug, and Group 3: commercial GLM tab; (c) FDC: fixed-dose combination (glimepiride/metformin) test and reference; (d) test and reference; (e) parent drug, metabolite 1, and metabolite 2; (f) parent drug and metabolite 1 (test, reference); (g) FDC: fixed-dose combination (glimepiride/pioglitazone). All values are given as Mean ± SD. ^1^ Value is provided as a Median. ^2^ Values in brackets represent the Range. ^3^ Values denote the serum concentration–time profiles. * Values are obtained through Non-Compartmental Analysis (NCA).

**Table 3:** PK parameters in healthy populations following IV route.

Sr. no.	Ref	Dose (mg)	(AUC) _0-∞_ (ng × h/mL)	C_max_ (ng/mL)	T_max_ (h)	t½ (h)	CL/F (mL/min)	CL (mL/min)
**1**	[63] ^1^	1 ^a^	N/S	243 ± 33	N/S	3.4 ± 2.0	N/S	48 ± 20
			N/S	24 ± 5	1.6 ± 0 4	2.7 ± 1.0	N/S	175 ± 94

Ref.: references; (AUC)0-∞: area under the curve from zero to infinity; C_max_: Maximum Plasma and Serum Concentration; T_max_: time to reach maximum concentration; t½: half-life; CL/F: oral clearance; CL: total body clearance; HP: healthy population; N/S: not stated; (a) parent drug and metabolite 1. All values are given as Mean ± SD. ^1^ Values denote the serum concentration–time profile.

**Table 4:** PK parameters in diseased populations.

Sr.	Ref.	Patient Characteristics	Dose (mg)		(AUC)0-∞ (ng × h/mL)		Cmax (ng/mL)	Tmax (h)	t½ (h)	CL/F (mL/min)	CLʀ (mL/min)
1	[44] *	DP (Southeast Asian)	2		51.9400		7.306	6.032	2.001	1283.53	N/S
2	[49]	DP (Japanese)	2 ^a^		706.2 ± 63.3		N/S	1.88± 0.21	3.28 ± 0.21	N/S	N/S
			1 ^b^		630.8 ± 93.6		N/S	2.15 ± 0.4	N/S	N/S	N/S
3	[51] ^3^	DP (Japanese)	1	*1/*1	292 ± 101.8 *		89.5 ± 37.8	N/S	N/S	^2^ 63 ± 24	N/S
				*1/*3	762.7 (654.6–870.9) ^1^		141.5 (123.8–159.2) ^1^	N/S	N/S	^2^ 22.5 (25.33–19.67) ^1^	N/S
4	[13]	DP	8	Parent drug ^c^	4004 ± 1319		547 ± 218	2.89 ± 0.90	12.6 ± 12.8 (2.80–54.85) ^1^	20.20 ± 7.23	N/S
					3281 ± 1362		410 ± 124	2.90 ± 0.89	8.89 ± 3.91	20.35 ± 8.9	N/S
				M 1 ^d^	1887 ± 754		180 ± 63	4.50 ± 0.78	9.76 ± 3.24	N/S	N/S
					1686 ± 476		135 ± 52	4.33 ± 0.82	11.6 ± 5.2	N/S	N/S
				M 2 ^e^	549 ± 212		50.3 ± 15.7	5.00 ± 1.14	7.09 ± 3.89	N/S	N/S
					383 ± 211		36.5 ± 14.7	5.36 ± 1.45	6.37 ± 4.87	N/S	N/S
5	[61]	DP with Renal Impairment	1, 2, 3, 4, 6, 8	Parent drug							
				^f^	S	1357 ± 452	359.2 ± 98.3	1.9 ± 0.2	2.28 ± 0.79	41.6 ± 18.5	N/S
					M	N/S	N/S	N/S	5.6 ± 3.0	68.1 ± 26.4	N/S
				^g^	S	622 ± 106	205.3 ± 29.0	2.7 ± 1.3	1.06 ± 0.23	81.1 ± 12.8	N/S
					M	N/S	N/S	N/S	3.2 ± 2.2	71.4 ± 17.5	N/S
				^h^	S	622 ± 226	194.0 ± 42.4	2.2 ± 1.0	2.19 ± 1.13	91.1 ± 36.5	N/S
					M	N/S	N/S	N/S	3.6 ± 1.9	97.8 ± 48.7	N/S
				M 1							
				^f^	S	N/S	70.8 ± 14.0	3.8 ± 0.8	2.79 ± 0.86	132.1 ± 66.4	49.6 ± 48.9
					M	N/S	N/S	N/S	3.75 ± 1.3	100.2 ± 30.4	31.8 ± 21.1
				^g^	S	N/S	93.0 ± 12.5	3.2 ± 0.8	2.32 ± 0.93	107.0 ± 33.7	9.2 ± 7.0
					M	N/S	N/S	N/S	3.96 ± 1.8	80.6 ± 21.1	11.8 ± 8.3
				^h^	S	N/S	103.6 ± 24.1	4.1 ± 2.3	4.88 ± 2.92	67.5 ± 39.6	4.9 ± 7.5
					M	N/S	N/S	N/S	8.0 ± 4.6	59.7 ± 33.3	2.1
				M 2							
				^f^	S	N/S	21.8 ± 8.5	4.8 ± 1.5	4.91 ± 2.94	306.5 ± 63.6	52.7 ± 40.7
					M	N/S	N/S	N/S	3.5 ± 1.1	184.2 ± 51.8	89.7 ± 61.0
				^g^	S	N/S	42.0 ± 2.6	5.7 ± 1.2	3.06 ± 0.50	140.8 ± 36.5	20.5 ± 12.7
					M	N/S	N/S	N/S	4.2 ± 1.2	111.9 ± 38.3	27.6 ± 9.4
				^h^	S	N/S	61.7 ± 25.9	7.0 ± 1.2	8.37 ± 1.90	61.5 ± 30.6	3.7 ± 7.5
					M	N/S	N/S	N/S	14.9 ± 12.3	62.0 ± 32.9	3.9 ± 3.5

Ref: references; (AUC)0-∞: area under the curve from zero to infinity; C_max_: Maximum Plasma and Serum Concentration; T_max_: time to reach maximum concentration; t½: half-life; CL/F: oral clearance; CLʀ: renal clearance; DP: diabetic patients; N/S: not stated; M 1: metabolite 1; M 2: metabolite 2; S: single-dose study; M: multiple-dose study. (a) 2 mg OD: once daily; (b) 1 mg BID: twice a day; (c) parent drug (normal weight and morbidly obese weight); (d) M 1 (normal weight and morbidly obese weight); (e) M 2 (normal weight and morbidly obese weight); (f) CrCL >50mL/min; (g) CrCL = 20–50 mL/min; (h) CrCL < 20 mL/min; All the values denote the serum concentration–time profiles. All values are given as Mean ± SD. ^1^ Data in brackets represent the Range. ^2^ The data represent CL (Total Body CL). ^3^ Data represents CYP2C9 Genotype alleles *1/*1 and *1/*3. * Values are obtained through Non-Compartmental Analysis (NCA).

**Table 5:** PD parameters of included studies.

Sr.	Ref.	Dose (mg)			HbA1c Levels (%)	Serum Glucose				Serum Insulin			
						Gmax (mmol/L)	AUGC (mmol × h/L)	Decremental AUC (0-3) (mmol × h/L)	Decremental AUC (0–7) (mmol × h/L)	Emax (pmol/L)	AUEC (pmol × h/L)	Incremental AUC (0–3) (pmol × h/L)	Incremental AUC (0–7) (pmol × h/L)
1	[26]	4	GLM		N/S	7.5 ± 1.9	16.17 ± 2.68	N/S	N/S	518.4 ± 262.8	850.2 ± 393	N/S	N/S
			EVO		N/S	7.0 ± 0.83	14.79 ± 1.76	N/S	N/S	633 ± 400.8	990.6 ± 636	N/S	N/S
		2	OD		6.9 ± 0.2	N/S	N/S	N/S	N/S	N/S	N/S	N/S	N/S
		1	BID		7.1 ± 0.1	N/S	N/S	N/S	N/S	N/S	N/S	N/S	N/S
3	[57] ^1^	0.5	CYP2C9 Genotypes										
			*1/*1 ^3^		N/S	N/S	N/S	−0.6 (−2.0–2.95)	−4.3 (−9.9–1.1)	N/S	N/S	N/S	N/S
			*1/*2 ^3^		N/S	N/S	N/S	−0.9 (−1.9–3.6)	−8.0 (−10.2–3.4)	N/S	N/S	N/S	N/S
			*1/*3 or *2/*3 ^3^		N/S	N/S	N/S	−0.1 (−−2.0–0.2)	−8.8 (−12.1–(−3.2))	N/S	N/S	N/S	N/S
4	[58]	0.5		GLM	N/S	N/S	N/S	−0.88 ± 1.27	1.96 ± 3.65	N/S	N/S	0.382 ± 0.182	0.964 ± 0.614
				GMF	N/S	N/S	N/S	−1.05 ± 2.13	1.5 ± 3.70	N/S	N/S	0.413 ± 0.303	0.88 ± 0.654
5	[59]	0.5		GLM	N/S	N/S	N/S	0.63 ± 1.45	0.40 ± 3.35	N/S	N/S	N/S	N/S
		100		FLV	N/S	N/S	N/S	–0.15 ± 2.10	–0.50 ± 4.92	N/S	N/S	N/S	N/S
		200, 400		FLC	N/S	N/S	N/S	1.15 ± 1.07	1.43 ± 3.48	N/S	N/S	N/S	N/S
6	[60]	1		GLM	N/S	N/S	N/S	0.57 ± 0.5	4.51 ± 1.07	N/S	N/S	N/S	N/S
				RIF	N/S	N/S	N/S	0.26 ± 0.55	5.05 ± 1.34	N/S	N/S	N/S	N/S
7	[61] *^2^*	1, 2, 3, 4, 6, 8			6.99 ± 1.2	N/S	N/S	N/S	N/S	N/S	N/S	N/S	N/S

Ref: reference; Gmax: Maximum Glucose Concentration; AUGC: area under the glucose–time curve; decremental AUC(0–3) net area below baseline of blood glucose from time zero to 3 h; decremental AUC(0–7) net area below baseline of blood glucose from time zero to 7 h; Max Inc.: maximum increase in serum glucose; Max Dec.: maximum decrease in serum glucose; Emax: maximum serum insulin level, AUEC: area under the serum insulin–time curve; incremental AUC(0–3) net area above baseline serum insulin from time zero to 3 h; decremental AUC(0–7) net area below baseline of serum insulin from zero to 7 h; Max Inc.: maximum increase in serum insulin; GLM: glimepiride; HP: healthy population; EVO: evogliptin; OD: once daily; BID: twice a day; GMF: gemfibrozil; FLV: fluvoxamine; FLC: fluconazole; RIF: rifampicin. All values are given as Mean ± SD. ^1^ Values are given as Median (Range). ^2^ Values denote the serum concentration–time profiles. ^3^ CYP2C9 Genotype alleles *1/*1, *1/*2, and *1/*3 or *2/*3.

**Table 6:** PK parameters of glimepiride in drug-drug interactions.

Sr.	Ref.	Dose (mg)		Drug	(AUC) _0-∞_ (ng × h/mL)	C_max_ (ng/mL)	T_max_ (h)	t½ (h)	CL/F (mL/min)	CLʀ (mL/min)
1	[26] ^1^	4	Parent drug	GLM	1672.7 ± 623. 9 (783.9–3293.8)	326.6 ± 98.5 (143.8–562.9)	3 ^2^ (1.5–5) ^3^	4.7 ± 2.2 (1.7–4.2)	45 ± 16.67 (20–85)	N/S
				GLM + EVO	1794.9 ± 653.2 (883.6–3282.8)	350.9 ± 97.4 (185.1–547.8)	4 ^2^ (1–6) ^3^	4.2 ± 2.0 (1.7–8.4)	41.67 ± 13.33 (20–75)	N/S
			M 1	GLM	611.9 ± 180.7 (309.4–1179.5)	81.3 ± 20.6 (47.7–135.3)	N/S	N/S	N/S	N/S
				GLM + EVO	652.6 ± 197.7 (387–1331.4)	84.2 ± 19 (55.8–137)	N/S	N/S	N/S	N/S
2	[27]	1		GLM	249.3 ± 213.55	34.35 ± 19.9	3 ^2^ (1.0–12.0) ^3^	5.89 ± 2.79	N/S	N/S
				GLM + ERT	296.7 ± 306.9	33.47 ± 15.79	4 ^2^ (1.5–12.0) ^3^	6.68 ± 4.02	N/S	N/S
3	[29]	4		GLM	N/S	173.7 ± 55.4	4.0 ^2^ (2.0–6.0) ^3^	13.3 ± 12.3	4.5 ± 1.3	N/S
				GLM + ROS	N/S	180.5 ± 65.3	3.0 ^2^ (1.5–5.0) ^3^	11.7 ± 5.0	4.4 ± 1.5	N/S
4	[31]	3		GLM	1498 ± 21.6	327.1 ± 3.4	1.5	2.6 ± 0.195	33.38 ± 0.4773	N/S
				GLM + GMF	3619.124 ± 58.0	1108.5 ± 44.52	1.5	4.1 ± 0.215	13.82 ± 0.2167	N/S
5	[35]	4		GLM	1215 ± 311	249 ± 56	1.0 ^2^ (0.9–3.93) ^3^	5.98 ^2^	58.5 ± 14.97	N/S
				GLM + COL	971 ± 244	233 ± 73	1.0 ^2^ (1.0–9.02) ^3^	6.59 ^2^	72.67 ± 16.62	N/S
				GLM 4hr before COL	1139 ± 318	256 ± 56	1.0 ^2^ (1.0–5.0) ^3^	5.52 ^2^	62.67 ± 15.5	N/S
6	[36]	4	Parent drug	GLM + GEMI	N/S	231.32 ± 71.58	3 ^2^	6.54 ± 2.30	N/S	N/S
				GLM	N/S	227.05 ± 72.64	4 ^2^	6.37 ± 2.9	N/S	N/S
			M 1	GLM + GEMI	N/S	29.58 ± 8.23	4 ^2^	5.87 ± 2.19	N/S	N/S
				GLM	N/S	28.26 ± 8.4	4 ^2^	6.42 ± 2.18	N/S	N/S
7	[39]	1–2								
				GLM	684 ± 211	136 ± 36	1.50 ^2^ (1.0–5.0) ^3^	6.8 ± 1.6	53.33 ± 16.67	N/S
				GLM + IPRA	720 ± 22	150 ± 41	1.00 ^2^ (1.0–5.0) ^3^	7.1 ± 1.8	50 ± 15	N/S
8	[46] *	4		GLM	4771.269	699.489	7.8742	16.1423	55.89	N/S
				GLM + DAPA	5375.324812	565.628	8.06713	16.751	49.6	N/S
9	[58]	0.5		GLM	137.9 ± 69.2	31.3 ± 5.2	1.5^2^ (1–4) ^3^	2.1 ± 0.6	N/S	N/S
				GLM + GMF	169.9 ± 82.7	35.6 ± 13.6	1.5^2^ (1–3) ^3^	2.3 ± 0.5	N/S	N/S
10	[59]	0.5		GLM	132.2 ± 61.4	32.7 ± 10.5	1.5 ^2^ (1–5) ^3^	2.0 ± 0.5	N/S	N/S
				GLM + FLV	175.4 ± 93.3	46.7 ± 18.6	1.5 ^2^ (1–1.5) ^3^	2.3 ± 0.5	N/S	N/S
				GLM + FLC	314.9 ± 122.2	49.2 ± 9.6	2.0 ^2^ (1.5–5) ^3^	3.3 ± 0.9	N/S	N/S
11	[60]	1		GLM	286.7 ± 35.1	64.2 ± 9.1	1.5 ^2^ (1.0–3.0) ^3^	2.6 ± 0.3	N/S	N/S
				GLM + RIF	190.3 ± 25.2	55.5 ± 7.2	1.0 ^2^ (1.0–2.0) ^3^	2.0 ± 0.2	N/S	N/S

Ref.: references; (AUC)0-∞: area under the curve from zero to infinity; C_max:_ Maximum Plasma and Serum Concentration; T_max:_ time to reach maximum concentration; t½: half-life; CL/F: oral clearance; CLʀ: renal clearance; N/S: not stated; GLM: glimepiride; EVO: evogliptin; ERT: ertugliflozin; ROS: rosuvastatin; GMF: gemfibrozil; COL: colesevelam; GEMI: gemigliptin; IPRA: ipragliflozin; DAPA: dapagliflozin; FLV: fluvoxamine; FLC: fluconazole; RIF: rifampicin. All values are given as Mean ± SD. ^1^ All values are given at a steady state condition ^2^ Values are given as Median. ^3^ Values given in the brackets represent the Range. * Values are obtained through Non-Compartmental Analysis (NCA).

## Data Availability

All the data used for this publication are either presented in the main article or are available as Supplementary Information.

## Supplementary Materials

The following supporting information can be downloaded at: https://www.mdpi.com/article/10.3390/ph18010122/s1, Supplementary Table S1: Quality Assessment of Included Articles based on JADAD Scoring, Supplementary Table S2: Quality Assessment of Included Articles based on Critical Appraisal Skills Program (CASP) Scoring, Supplementary Table S3: Quality Assessment of Included Articles based on Critical Appraisal Clinical Pharmacokinetics Tool (CACPK) Scoring, and Supplementary Table S4: Quality Assessment of Included Articles based on Cochrane Collaboration Tool (CCT), and Supplementary Table S5: Screening and Exclusion of articles based on their titles, abstract, animal studies, reviews, and accessibility.
