# Supplementary material for: The Clinical Pharmacokinetics and Pharmacodynamics of Glimepiride—A Systematic Review and Meta-Analysis
Source: Pharmaceuticals (Basel). 2025 Jan 17;18(1):122. doi: 10.3390/ph18010122 (PMC11768776; doi:10.3390/ph18010122)
Supplement: Supplementary file 1 [file pharmaceuticals-18-00122-s001.zip › pharmaceuticals-3384662-supplementary.pdf]

The Clinical Pharmacokinetics and Pharmacodynamics of Glimepiride – A systematic review and meta-analysis

Supplementary Information

Supplemenary Table S1: Quality Assessment of Included Articles based on JADAD Scoring

**Sr. no**	**Reference**	Was the study described as randomized?	Was the method used to generate the sequence of randomization described and appropriate?	Was the study described as double blind?	Was the method of double blinding described and appropriate?	Was there a description of withdrawls and dropouts?	Total score
1	[1]	1	1	0	0	1	3
2	[2]	1	1	0	0	0	2
3	[3]	0	0	0	0	0	0
4	[4]	1	1	0	0	1	3
5	[5]	0	0	0	0	0	0
6	[6]	1	1	0	0	0	2
7	[7]	0	0	0	0	0	0
8	[8]	0	0	0	0	0	0
9	[9]	1	1	1	0	1	4
10	[10]	1	1	0	0	1	3
11	[11]	1	1	0	0	1	3

Sr. no	Reference	Was the study described as randomized?	Was the method used to generate the sequence of randomization described and appropriate?	Was the study described as double blind?	Was the method of double blinding described and appropriate?	Was there a description of withdrawls and dropouts?	Total score
12	[12]	0	0	0	0	0	0
13	[13]	1	1	0	0	1	3
14	[14]	1	1	0	0	1	3
15	[15]	1	1	0	0	0	2
16	[16]	0	0	0	0	0	0
17	[17]	0	0	0	0	0	0
18	[18]	0	0	0	0	0	0
19	[19]	0	0	0	0	0	0
20	[20]	1	1	0	0	1	3
21	[21]	1	1	0	0	1	3
22	[22]	1	1	0	0	1	3
23	[23]	1	1	0	0	1	3
24	[24]	1	1	0	0	1	3
25	[25]	1	1	0	0	1	3
26	[26]	0	0	0	0	0	0

**Sr. no**	**Reference**	Was the study described as randomized?	Was the method used to generate the sequence of randomization described and appropriate?	Was the study described as double blind?	Was the method of double blinding described and appropriate?	Was there a description of withdrawls and dropouts?	Total score
27	[27]	1	1	0	0	1	3
28	[28]	0	0	0	0	0	0
29	[29]	0	0	0	0	0	0
30	[30]	0	0	0	0	0	0
31	[31]	1	1	0	0	1	3
32	[32]	0	0	0	0	0	0
33	[33]	0	0	0	0	0	0
34	[34]	1	0	0	0	0	1
35	[35]	1	1	1	0	0	3
36	[36]	1	1	0	0	1	3
37	[37]	0	0	0	0	1	1
38	[38]	1	1	1	0	0	3
39	[39]	1	1	0	0	0	2
40	[40]	0	0	0	0	0	0

Supplemenary Table S2: Quality Assessment of Included Articles based on Critical Appraisal Skills Program (CASP) Scoring

Sr. no	Reference	1	2	3	4	5	6	7	8	9	10	CASP Score
1	[1]	Y	Y	Y	Y	Y	Y	CT	Y	Y	Y	9
2	[2]	Y	Y	Y	Y	Y	Y	N	Y	Y	Y	9
3	[3]	Y	Y	CT	Y	Y	Y	N	Y	Y	Y	8
4	[4]	Y	Y	Y	Y	Y	Y	N	Y	Y	Y	9
5	[5]	Y	Y	Y	Y	Y	Y	N	Y	Y	Y	9
6	[6]	Y	Y	Y	Y	Y	Y	N	Y	Y	Y	9
7	[7]	CT	CT	Y	Y	Y	Y	N	Y	Y	Y	7
8	[8]	Y	Y	Y	Y	Y	Y	N	Y	Y	Y	9
9	[9]	Y	Y	Y	Y	Y	Y	N	Y	Y	Y	9
10	[10]	Y	Y	Y	Y	Y	CT	CT	N	Y	Y	7

**Sr. no**	**Reference**	**1**	**2**	**3**	**4**	**5**	**6**	**7**	**8**	**9**	**10**	**CASP Score**
11	[11]	Y	Y	Y	Y	Y	Y	N	Y	Y	Y	9
12	[12]	Y	Y	Y	Y	Y	Y	N	Y	Y	Y	9
13	[13]	Y	Y	Y	Y	Y	Y	N	Y	Y	Y	9
14	[14]	Y	Y	Y	Y	Y	Y	N	Y	Y	Y	9
15	[15]	y	y	y	y	y	CT	N	Y	Y	Y	8
16	[16]	Y	Y	Y	Y	Y	CT	N	CT	Y	Y	7
17	[17]	Y	Y	Y	Y	Y	CT	CT	N	Y	Y	7
18	[18]	Y	Y	Y	Y	CT	CT	N	N	Y	Y	6
19	[19]	Y	Y	Y	Y	CT	CT	N	N	Y	Y	6
20	[20]	Y	Y	Y	Y	Y	Y	N	Y	Y	Y	9
21	[21]	Y	Y	Y	Y	Y	Y	CT	Y	Y	Y	9
22	[22]	Y	Y	Y	Y	Y	Y	CT	Y	Y	Y	9

Sr. no	Reference	1	2	3	4	5	6	7	8	9	10	CASP Score
23	[23]	Y	Y	Y	Y	Y	Y	N	Y	Y	Y	9
24	[24]	Y	Y	Y	Y	Y	Y	N	Y	Y	Y	9
25	[25]	Y	Y	Y	Y	Y	Y	N	Y	Y	Y	9
26	[26]	Y	Y	Y	Y	Y	Y	N	Y	Y	Y	9
27	[27]	Y	Y	Y	Y	Y	Y	CT	Y	Y	Y	9
28	[28]	Y	Y	Y	Y	Y	Y	CT	Y	Y	Y	9
29	[29]	Y	Y	Y	Y	CT	CT	N	CT	Y	Y	6
30	[30]	Y	Y	Y	Y	CT	CT	N	N	Y	Y	6
31	[31]	Y	Y	Y	Y	Y	Y	N	Y	Y	Y	9
32	[32]	Y	Y	Y	Y	CT	CT	CT	CT	Y	Y	6
33	[33]	Y	Y	Y	Y	Y	Y	CT	Y	Y	Y	9
34	[34]	Y	Y	Y	Y	Y	Y	CT	Y	Y	Y	9

Sr. no	Reference	1	2	3	4	5	6	7	8	9	10	CASP Score
35	[35]	Y	Y	Y	Y	Y	Y	CT	Y	Y	Y	9
36	[36]	Y	Y	Y	Y	Y	Y	CT	Y	Y	Y	9
37	[37]	Y	Y	Y	Y	Y	Y	CT	Y	Y	Y	9
38	[38]	Y	Y	Y	Y	Y	Y	CT	Y	Y	Y	9
39	[39]	Y	Y	Y	Y	Y	Y	CT	Y	Y	Y	9
40	[40]	Y	Y	Y	Y	N	N	CT	N	Y	Y	6

Y= YES, N= NO, CT= CAN'T TELL

Questions:
Q 1: Was there a clear statement of the aims of the research?
Q 2: Is a qualitative methodology appropriate?
Q 3: Was the research design appropriate to address the aims of the research?
Q 4: Are the study's theoretical underpinnings clear, consistent, and conceptually coherent?
Q 5: Was the recruitment strategy appropriate to the aims of the search?
Q 6: Was the data collected in a way that addressed the research issue?
Q 7: Has the relationship between researchers and participants been adequately considered?
Q 8: Have ethical issues been taken into consideration?
Q 9: Was the data analysis sufficiently rigrous?
Q 10: Is there a clear statement of findings?

Supplemenary Table S3: Quality Assessment of Included Articles based on Crtical Appraisal Clinical Pharmacokinetics Tool (CACPK) Scoring

Sr. no	Reference	Q1	Q2	Q3	Q4	Q5	Q6	Q7	Q8	Q9	Q10	Q11	Q12	Q13	Q14	Q15	Q16	Q17	Q18	Q19	Q20	Q21	Total Score
1	[1]	Y	Y	Y	Y	N	Y	Y	IDK	Y	Y	Y	IDK	N	Y	IDK	Y	N	Y	Y	Y	Y	15
2	[2]	Y	Y	Y	Y	IDK	Y	Y	IDK	Y	Y	Y	IDK	IDK	Y	Y	Y	N	IDK	Y	Y	Y	15
3	[3]	Y	Y	Y	Y	N	N	N	IDK	Y	Y	Y	IDK	Y	IDK	Y	Y	Y	Y	N	N	IDK	12
4	[4]	Y	Y	Y	Y	Y	Y	Y	IDK	Y	Y	Y	IDK	Y	Y	Y	Y	N	Y	Y	Y	Y	18
5	[5]	Y	Y	Y	Y	N	N	Y	IDK	Y	Y	Y	IDK	Y	IDK	IDK	Y	Y	Y	Y	Y	Y	15
6	[6]	Y	Y	Y	Y	Y	Y	Y	IDK	Y	Y	Y	IDK	Y	IDK	IDK	Y	N	Y	Y	Y	Y	16
7	[7]	Y	Y	Y	Y	Y	N	Y	IDK	Y	Y	Y	IDK	Y	Y	Y	Y	N	Y	Y	Y	Y	17
8	[8]	Y	Y	Y	Y	IDK	N	Y	IDK	Y	Y	Y	IDK	Y	IDK	IDK	Y	N	N	Y	Y	N	12
9	[9]	Y	Y	Y	Y	Y	N	Y	IDK	Y	Y	Y	IDK	Y	Y	Y	Y	N	Y	Y	Y	Y	17
10	[10]	Y	Y	Y	Y	Y	Y	Y	IDK	Y	Y	Y	IDK	Y	Y	Y	Y	N	Y	Y	Y	Y	18
11	[11]	Y	Y	Y	Y	Y	Y	Y	IDK	Y	Y	Y	IDK	Y	Y	Y	Y	N	Y	Y	Y	Y	18
12	[12]	Y	Y	Y	Y	N	N	Y	IDK	Y	Y	Y	IDK	Y	Y	Y	Y	N	IDK	Y	Y	N	14
13	[13]	Y	Y	Y	Y	Y	Y	Y	IDK	Y	Y	Y	IDK	Y	Y	Y	Y	N	Y	Y	Y	Y	18
14	[14]	Y	Y	Y	Y	Y	Y	Y	IDK	Y	Y	Y	IDK	IDK	Y	Y	Y	N	Y	Y	Y	Y	17
15	[15]	Y	Y	Y	Y	Y	Y	Y	IDK	Y	Y	Y	IDK	Y	Y	Y	Y	N	Y	Y	Y	Y	18

Sr. no	Reference	Q1	Q2	Q3	Q4	Q5	Q6	Q7	Q8	Q9	Q10	Q11	Q12	Q13	Q14	Q15	Q16	Q17	Q18	Q19	Q20	Q21	Total Score
16	[16]	Y	Y	Y	Y	Y	Y	Y	IDK	Y	Y	Y	IDK	Y	N	N	IDK	N	N	IDK	IDK	IDK	11
17	[17]	Y	Y	Y	Y	Y	Y	Y	IDK	Y	Y	Y	IDK	Y	IDK	IDK	Y	N	Y	Y	Y	Y	16
18	[18]	Y	Y	Y	Y	IDK	IDK	Y	IDK	Y	Y	Y	IDK	Y	N	N	Y	N	Y	Y	Y	Y	14
19	[19]	Y	Y	Y	Y	N	N	Y	IDK	Y	Y	Y	IDK	Y	Y	IDK	Y	N	IDK	IDK	IDK	IDK	11
20	[20]	Y	Y	Y	Y	Y	Y	Y	IDK	Y	Y	Y	IDK	Y	Y	Y	Y	N	Y	Y	Y	Y	18
21	[21]	Y	Y	Y	Y	Y	Y	Y	Y	Y	Y	Y	IDK	N	IDK	IDK	Y	N	Y	Y	Y	Y	16
22	[22]	Y	Y	Y	Y	Y	Y	Y	IDK	Y	Y	Y	IDK	Y	Y	Y	Y	N	Y	Y	Y	Y	18
23	[23]	Y	Y	Y	Y	Y	N	Y	IDK	Y	Y	Y	IDK	Y	Y	Y	Y	Y	Y	Y	Y	Y	19
24	[24]	Y	Y	Y	Y	Y	N	Y	IDK	Y	Y	Y	IDK	N	Y	Y	Y	N	Y	Y	Y	N	15
25	[25]	Y	Y	Y	Y	Y	Y	Y	IDK	Y	Y	Y	IDK	Y	Y	Y	Y	N	Y	Y	Y	Y	18
26	[26]	Y	Y	Y	Y	Y	IDK	Y	IDK	Y	Y	Y	IDK	N	Y	Y	Y	N	Y	Y	Y	Y	16
27	[27]	Y	Y	Y	Y	Y	N	Y	IDK	Y	Y	Y	IDK	Y	Y	Y	Y	N	Y	Y	Y	Y	17
28	[28]	Y	Y	Y	Y	Y	Y	Y	IDK	Y	Y	Y	IDK	Y	Y	Y	Y	N	Y	Y	IDK	IDK	16
29	[29]	Y	Y	Y	Y	Y	N	Y	IDK	Y	Y	Y	IDK	Y	Y	Y	Y	N	Y	Y	Y	Y	17
30	[30]	Y	Y	Y	Y	Y	IDK	Y	IDK	Y	Y	Y	IDK	Y	N	IDK	Y	N	IDK	Y	Y	IDK	13
31	[31]	Y	Y	Y	Y	Y	Y	Y	IDK	Y	Y	Y	IDK	Y	IDK	IDK	Y	N	Y	Y	Y	Y	16
32	[32]	Y	Y	Y	IDK	IDK	IDK	IDK	IDK	Y	Y	N	IDK	Y	N	N	Y	N	IDK	N	N	N	7
33	[33]	Y	Y	Y	Y	Y	N	Y	IDK	Y	Y	N	IDK	N	N	N	Y	N	Y	Y	Y	Y	13

Sr. no	Reference	Q 1	Q 2	Q 3	Q4	Q5	Q6	Q7	Q8	Q 9	Q1 0	Q1 1	Q1 2	Q1 3	Q1 4	Q1 5	Q1 6	Q1 7	Q1 8	Q1 9	Q2 0	Q2 1	Total Score
34	[34]	Y	Y	Y	Y	Y	IDK	Y	IDK	Y	Y	Y	IDK	Y	Y	Y	Y	N	Y	Y	Y	Y	17
35	[35]	Y	Y	Y	Y	Y	N	Y	IDK	Y	Y	Y	IDK	Y	N	N	Y	Y	Y	Y	Y	Y	16
36	[36]	Y	Y	Y	Y	Y	N	Y	IDK	Y	Y	Y	IDK	Y	N	N	Y	Y	Y	Y	Y	Y	16
37	[37]	Y	Y	Y	Y	Y	Y	Y	IDK	Y	Y	Y	IDK	Y	Y	Y	Y	N	N	Y	Y	N	16
38	[38]	Y	Y	Y	Y	Y	Y	Y	IDK	Y	Y	Y	IDK	N	Y	Y	Y	N	Y	Y	Y	Y	17
39	[39]	Y	Y	Y	Y	IDK	Y	Y	IDK	Y	Y	Y	IDK	IDK	Y	Y	Y	N	Y	Y	Y	Y	16
40	[40]	Y	Y	Y	Y	IDK	N	N	IDK	Y	Y	N	IDK	IDK	N	N	Y	N	N	IDK	IDK	IDK	10

Y= YES, N= NO, IDK= I DON'T KNOW

Questions:

Q 1: Was a clear description of the objectives of the study provided?
Q 2: Was a clear and comprehensive rationale provided to support the purpose of the study?
Q 3: Was the chosen study design appropriately selected and justified?
Q 4: Was the dosing (i.e. dose, route of administration, and dosing interval) of the drug in the study justified for the intended study?
Q 5: Were the outcome measures endpoints of the study appropriate to address the objectives of the study?
Q 6: Were the exclusion criteria of participants included AND appropriate for the intended outcomes of the study?
Q 7: Where applicable, were the relevant baseline characteristics of the participants adequately described?
Q 8: Were plausible interacting covariates described *a priori* or in post hoc evaluation?
Q 9: Was the description of the used biological sample analytical methods sample analysis methods or citations of prior validation studies provided in the publication or affiliated appendix?
Q 10: Was the method of data sampling of analytics appropriate for the study?
Q 11: Was a clear description of the sampling site provided and justified?
Q 12: Was the number of half-lives elapsed within the sampling period appropriate for the analyzed drug?
Q 13: Were sample storage conditions appropriate and described in a manner that could be accurately replicated?
Q 14: If applicable, was there a clear description of the pharmacokinetic model, its development, validation and justification for use?
Q 15: Was the described population pharmacokinetic approach validation method appropriate for the analysis?
Q 16: Were the essential pharmacokinetic parameters required to make the results applicable in clinical settings included?
Q 17: Were the pharmacokinetic equations used to calculate the patient's pharmacokinetic parameters presented or cited within the article?
Q 18: Were the chosen statistical tests and software to perform the statistical analysis appropriate to achieve the study objectives?
Q 19: Were all patients enrolled in the study accounted for?
Q 20: In the event of missing data or outliers, was the process for analysis justified and appropriate?

Q 21: Were appropriate summary statistics to describe centrality and variance used to present the pharmacokinetic results?

Supplemenary Table S4: Quality Assessment of Included Articles based on Cochrane Collaboration Tool (CCT)

Sr. no	Reference	Random sequence (selection bias)	Allocation concealment (selection bias)	Blinding of participant and researchers (performance bias)	Blinding of outcome assessment (detection bias)	Incomplete outcome data (attrition bias)	Selective reporting (reporting bias)	Other bias	Total score
1	[1]	LR	HR	HR	LR	LR	LR	LR	5
2	[2]	HR	HR	UR	LR	LR	LR	LR	4
3	[3]	LR	UR	UR	UR	UR	UR	UR	1
4	[4]	LR	HR	HR	HR	LR	LR	LR	4
5	[5]	LR	UR	UR	UR	UR	UR	LR	2
6	[6]	LR	HR	HR	UR	LR	LR	LR	4
7	[7]	UR	UR	HR	UR	LR	LR	LR	4
8	[8]	UR	HR	HR	UR	LR	LR	LR	3
9	[9]	LR	LR	LR	UR	LR	LR	LR	6
10	[10]	UR	UR	UR	UR	LR	LR	LR	3
11	[11]	HR	UR	HR	UR	LR	LR	LR	3
12	[12]	UR	UR	UR	UR	UR	UR	LR	1
13	[13]	UR	HR	HR	UR	UR	UR	LR	1
14	[14]	LR	HR	UR	UR	LR	LR	LR	4
15	[15]	HR	UR	UR	UR	UR	UR	LR	1
16	[16]	UR	UR	UR	UR	UR	UR	LR	1
17	[17]	HR	UR	UR	UR	LR	LR	LR	3
18	[18]	UR	UR	UR	UR	LR	LR	LR	3

Sr. no	Reference	Random sequence (selection bias)	Allocation concealment (selection bias)	Blinding of participant and researchers (performance bias)	Blinding of outcome assessment (detection bias)	Incomplete outcome data (attrition bias)	Selective reporting (reporting bias)	Other bias	Total score
19	[19]	UR	UR	UR	UR	LR	LR	LR	3
20	[20]	HR	HR	HR	HR	LR	LR	LR	3
21	[21]	HR	UR	UR	UR	LR	LR	LR	3
22	[22]	LR	UR	UR	UR	LR	LR	LR	4
23	[23]	UR	UR	UR	UR	LR	LR	LR	3
24	[24]	LR	UR	UR	UR	LR	LR	LR	4
25	[25]	HR	UR	UR	UR	LR	LR	LR	3
26	[26]	HR	UR	UR	UR	LR	LR	LR	3
27	[27]	LR	UR	UR	UR	LR	LR	LR	4
28	[28]	UR	UR	UR	UR	UR	LR	LR	2
29	[29]	UR	UR	UR	UR	LR	LR	LR	3
30	[30]	UR	UR	UR	UR	LR	LR	LR	3
31	[31]	LR	UR	UR	UR	LR	LR	LR	4
32	[32]	UR	UR	UR	UR	LR	LR	LR	3
33	[33]	HR	UR	UR	UR	LR	LR	LR	3
34	[34]	LR	UR	LR	LR	LR	LR	LR	6
35	[35]	LR	UR	LR	LR	LR	LR	LR	6
36	[36]	LR	UR	LR	UR	LR	LR	LR	5
37	[37]	HR	UR	HR	HR	LR	LR	LR	3
38	[38]	LR	LR	LR	UR	LR	LR	LR	6
39	[39]	LR	LR	LR	UR	LR	LR	LR	6

Sr. no	Reference	Random sequence (selection bias)	Allocation concealment (selection bias)	Blinding of participant and researchers (performance bias)	Blinding of outcome assessment (detection bias)	Incomplete outcome data (attrition bias)	Selective reporting (reporting bias)	Other bias	Total score
40	[40]	UR	UR	UR	UR	UR	UR	LR	1

HR= HIGH RISK, LR= LOW RISK, UR= UNKNOWN RISK

Supplementary Table S5: Screening and Exclusion of articles based on their titles, abstract, animal studies, reviews and accessibilty

Sr no	Reference	Reason
1	(1991). "Author index volumes 501–550." Journal of Chromatography A 551(1): 1-155.	Title
2	(1991). "Compound index volumes 501–550." Journal of Chromatography A 551(1): 316-547.	Title
3	(1993). "Liquid column chromatography." Journal of Chromatography A 649(2): B143-B198.	Title
4	(1993). "Planar chromatography." Journal of Chromatography A 649(2): B224-B235.	Title
5	(1995). "Abstracts of the “Internistendagen” Veldhoven, Netherlands, 27–28 April, 1995." The Netherlands Journal of Medicine 46(5): A45-A81.	Title
6	(1995). "Cardiovascular alterations in diabetes mellitus." Journal of Molecular and Cellular Cardiology 27(6): A421-A434.	Title
7	(1996). "Glimepiride for NIDDM." Med Lett Drugs Ther 38(975): 47-48.	Title
8	(1996). "Master author index to volumes 294–316(1996)." European Journal of Pharmacology 294: 1-52.	Title
9	(1997). "Master keyword index to volumes 317–339(1997)." European Journal of Pharmacology 317-339: 47-185.	Title
10	(2002). "2002 Abstracts of Contributed Papers." Journal of the American Pharmaceutical Association (1996) 42(2): 283-335.	Title
11	(2005). "LIST OF CONTENTS." Journal of Pharmaceutical and Biomedical Analysis 36(5): 1129-1137.	Title
12	(2005). "SUBJECT INDEX." Journal of Pharmaceutical and Biomedical Analysis 39(5): 1099-1104.	Title
13	(2005). "Volume Contents." Journal of Hepatology 42(6): III-XVI.	Title
14	(2006). "Author Index Volumes 307-325 (2006)." International Journal of Pharmaceutics 325(1): e1-e22.	Title
15	(2006). "Rosiglitazone/glimepiride (Avandaryl) for diabetes." Med Lett Drugs Ther 48(1230): 22-24.	Title

16	(2006). "Subject Index Volumes 307-325 (2006)." International Journal of Pharmaceutics 325(1): e23-e36.	Title
17	(2007). "Pioglitazone/glimepiride (Duetact) for diabetes." Med Lett Drugs Ther 49(1253): 9-11.	Title
18	(2007). "References." American Journal of Kidney Diseases 49(2, Supplement 2): S160-S179.	Title
19	(2008). "The Challenges and Opportunities of Managing Diabetes in Long-Term Care." Journal of the American Medical Directors Association 9(4, Supplement): 1-20.	Title
20	(2009). "3rd BBBB-Bosphorus International Conference on Pharmaceutical Sciences." European Journal of Pharmaceutical Sciences 38(1, Supplement): S1-S220.	Title
21	(2010). "National Kidney Foundation 2010 Spring Clinical Meetings Abstracts." American Journal of Kidney Diseases 55(4): B1-B31.	Title
22	(2010). "Thursday Posters." Journal of the American Society for Mass Spectrometry 21(5, Supplement): S122-S151.	Title
23	(2013). "5th International BBBB Conference / Abstract Book." European Journal of Pharmaceutical Sciences 50: 1-234.	Title
24	(2016). "XXIV World Allergy Congress 2015." World Allergy Organization Journal 9: 14.	Title
25	(2018). "119th Annual Meeting of the American Association of Colleges of Pharmacy, Boston, Massachusetts, July 21-25, 2018." American Journal of Pharmaceutical Education 82(5): 7158.	Title
26	(2020). "Graphical Abstract TOC." Journal of Drug Delivery Science and Technology 60: 102251.	Title
27	Abbink, E. J., et al. (2004). "Compared to glibenclamide, repaglinide treatment results in a more rapid fall in glucose level and beta-cell secretion after glucose stimulation." Diabetes/metabolism research and reviews 20(6): 466-471.	Review
28	Abe, M., et al. (2011). "Antidiabetic agents in patients with chronic kidney disease and end-stage renal disease on dialysis: metabolism and clinical practice." Current drug metabolism 12(1): 57-69.	Review
29	Abou-Omar, M. N., et al. (2021). "Validation of a novel UPLC-MS/MS method for estimation of metformin and empagliflozin simultaneously in human plasma using freezing lipid precipitation approach and its application to pharmacokinetic study." Journal of Pharmaceutical and Biomedical Analysis 200: 114078.	Title
30	Abou-Taleb, B. A., et al. (2020). "In-vitro and in-vivo performance of locally manufactured glimepiride tablet generics compared to the innovator (Amaryl(®)) tablets." Drug Dev Ind Pharm 46(2): 192-199.	Full-Length Text
31	Adar, L. (2008). Genetic Polymorphism and Utilization of Phenytoin as a Probe Marker for CYP2C9 Phenotyping, Hebrew University.	Title
32	Ahmad, M. Z., et al. (2022). "Nanoemulgel as an approach to improve the biopharmaceutical performance of lipophilic drugs: Contemporary research and application." Journal of Drug Delivery Science and Technology 72: 103420.	Title
33	Ahmed, O. A., et al. (2016). "Mechanistic analysis of Zein nanoparticles/PLGA triblock in situ forming implants for glimepiride." Int J Nanomedicine 11: 543-555.	Abstract
34	Akbar, S. and S. Akbar (2020). "Gymnema sylvestre R. Br.(Apocynaceae) (Syns.: Asclepias germinata Roxb.; Periploca sylvestris Retz.)." Handbook of 200 Medicinal Plants: A Comprehensive Review of Their Traditional Medical Uses and Scientific Justifications: 981-990.	Review
35	Akhlaghi, F., et al. (2017). "Clinical pharmacokinetics and pharmacodynamics of antihyperglycemic medications in children and adolescents with type 2 diabetes mellitus." Clinical pharmacokinetics 56: 561-571.	Review

36	Akhtar, N., et al. (2020). "Self-Generating nano-emulsification techniques for alternatively-routed, bioavailability enhanced delivery, especially for anti-cancers, anti-diabetics, and miscellaneous drugs of natural, and synthetic origins." Journal of Drug Delivery Science and Technology 58: 101808.	Title
37	Akhtar, W., et al. (2016). "The therapeutic journey of pyridazinone." European Journal of Medicinal Chemistry 123: 256-281.	Review
38	Alexovič, M., et al. (2018). "Achievements in robotic automation of solvent extraction and related approaches for bioanalysis of pharmaceuticals." Journal of Chromatography B 1092: 402-421.	Review
39	Algeelani, S., et al. (2018). "Inhibitory effects of sulfonylureas and non-steroidal anti-inflammatory drugs on in vitro metabolism of canagliflozin in human liver microsomes." Biopharm Drug Dispos 39(3): 135-142.	Title
40	ALI, S., et al. (2013). "Sulfonylureas: Physico-chemical Properties, Analytical Methods of Determination." Oral Antidiabetics 119: 73.	Review
41	Ali, S. K., et al. (2019). "Pharmaceutical quality evaluation of different glimepiride brands marketed in Karachi (Pakistan): In pursuance to global issue of availability and affordability of quality medicines." Pak J Pharm Sci 32(6): 2709-2715.	Review
42	Alkattan, A. and E. Alsalameen (2021). "Polymorphisms of genes related to phase-I metabolic enzymes affecting the clinical efficacy and safety of clopidogrel treatment." Expert opinion on drug metabolism & toxicology 17(6): 685-695.	Review
43	Almeling, S., et al. (2012). "Charged aerosol detection in pharmaceutical analysis." Journal of Pharmaceutical and Biomedical Analysis 69: 50-63.	Review
44	Ambery, P., et al. (2016). "Open-label randomized non-inferiority trial of a fixed-dose combination of glimepiride and atorvastatin for the treatment of people whose Type 2 diabetes is uncontrolled on metformin." Diabetic Medicine 33(8): 1084-1093.	Abstract
45	Ambhore, J. P., et al. (2023). "Medicinal chemistry of non-peptidomimetic dipeptidyl peptidase IV (DPP IV) inhibitors for treatment of Type-2 diabetes mellitus: Insights on recent development." Journal of Molecular Structure 1284: 135249.	Title
46	Ammazzalorso, A., et al. (2019). "Multitarget PPARγ agonists as innovative modulators of the metabolic syndrome." European Journal of Medicinal Chemistry 173: 261-273.	Review
47	Annaji, M., et al. (2020). "Application of Extrusion-Based 3D Printed Dosage Forms in the Treatment of Chronic Diseases." Journal of Pharmaceutical Sciences 109(12): 3551-3568.	Review
48	Antonesi, I. M., et al. (2011). "PHARMACOKINETIC MODELING OF GLIMEPIRIDE PLASMA CON-CENTRATION IN HEALTHY SUBJECTS." The Medical-Surgical Journal 115(3): 949-953.	Title
49	Antonesi, I. M., et al. (2011). "Pharmacokinetic modeling of glimepiride plasma concentration in healthy subjects." Rev Med Chir Soc Med Nat Iasi 115(3): 949-953.	Title
50	Ariano, R. E., et al. (2017). "No role for patient body weight on renal function assessment for drug dosing." Journal of Antimicrobial Chemotherapy 72(6): 1802-1811.	Title
51	Armor, B. L. and M. L. Britton (2004). "Diabetes mellitus non-glucose monitoring: point-of-care testing." Annals of Pharmacotherapy 38(6): 1039-1047.	Review
52	Arulmozhi, D. K. and B. Portha (2006). "GLP-1 based therapy for type 2 diabetes." European Journal of Pharmaceutical Sciences 28(1): 96-108.	Review
53	Assendelft, W. and H. Guchelaar (2011). "JJ Swen, JAM Wessels, A Krabben." Translating Pharmacogenetics to Primary Care 11(11): 125.	Title

54	Astrup, A., et al. (2005). "Diabetes and the endocrine pancreas." Diabetes Care 28: 956-962.	Title
55	Ataby, O. A., et al. (2013). "Genetic polymorphism of cytochrome P450 2C9 (CYP2C9) in two ethnic groups in Iran." Am J Biomed Sci 5(3): 177-187.	Title
56	Awad, A., et al. (2018). "3D printed medicines: A new branch of digital healthcare." International Journal of Pharmaceutics 548(1): 586-596.	Review
57	Aziz, A., et al. (2017). "Pharmacokinetic study of glimepiride alone and in combination with atorvastatin in healthy male volunteers." LATIN AMERICAN JOURNAL OF PHARMACY 36(1): 151-156.	Title
58	Babadi, D., et al. (2021). "Biopharmaceutical and pharmacokinetic aspects of nanocarrier-mediated oral delivery of poorly soluble drugs." Journal of Drug Delivery Science and Technology 62: 102324.	Review
59	Babadi, D., et al. (2020). "Nanoformulation strategies for improving intestinal permeability of drugs: A more precise look at permeability assessment methods and pharmacokinetic properties changes." Journal of Controlled Release 321: 669-709.	Review
60	Baciewicz, A. M., et al. (2008). "Update on Rifampin and Rifabutin Drug Interactions." The American Journal of the Medical Sciences 335(2): 126-136.	Review
61	Badian, M., et al. (1996). "Pharmacokinetics and pharmacodynamics of the hydroxy-metabolite of glimepiride (Amarylregistered trade mark) after intravenous administration." Drug metabolism and drug interactions. 13(1): 69-85.	Not accessible
62	Badian, M., et al. (1992). "Determination of the absolute bioavailability of glimepiride (HOE 490), a new sulphonylurea." Int J Clin Pharmacol Ther Toxicol 30(11): 481-482.	Full-Length Text
63	Badian, M., et al. (1996). "Pharmacokinetics and pharmacodynamics of the hydroxy-metabolite of glimepiride (Amaryl®) after intravenous administration." Drug Metabolism and Drug Interactions 13(1): 69-85.	Full-Length Text
64	Bae, J.-w., et al. (2011). "Frequency of CYP2C9 alleles in Koreans and their effects on losartan pharmacokinetics." Acta Pharmacologica Sinica 32(10): 1303-1308.	Title
65	Baghel, U. S., et al. (2012). "Gradient HPLC method development and validation for Simultaneous estimation of Rosiglitazone and Gliclazide." Asian Pacific Journal of Tropical Disease 2: S756-S760.	Title
66	Bahloul, B., et al. (2023). "Nanomedicine-based potential phyto-drug delivery systems for diabetes." Journal of Drug Delivery Science and Technology 82: 104377.	Title
67	Banerjee, K., et al. (2017). "Role of Ranolazine in cardiovascular disease and diabetes: Exploring beyond angina." International Journal of Cardiology 227: 556-564.	Review
68	Bansal, G., et al. (2020). "An overview on medicinal perspective of thiazolidine-2,4-dione: A remarkable scaffold in the treatment of type 2 diabetes." Journal of Advanced Research 23: 163-205.	Title
69	Barman, B., et al. (2018). "Poisoning in elderly." Indian Journal of Medical Specialities 9(3): 113-117.	Title
70	Barna, O. and E. Pogrebnyak (2018). "Ефективний та безпечний контроль глікемії при використанні гліклазиду MR та глімепіриду в лікуванні цукрового діабету 2 типу." Ліки України(2 (218)): 23-29.	Language
71	Basit, A., et al. (2012). "Glimepiride: evidence-based facts, trends, and observations." Vascular health and risk management: 463-472.	Review
72	Becić, F., et al. (2003). "[Glimepiride--an oral antidiabetic agent]." Med Arh 57(2): 125-127.	Title
73	Becker, M. (2009). Individualizing Pharmacotherapy: Genetic factors and co-prescribed drugs affecting pharmacotherapy.	Title

74	Beckett, R. D. and A. L. Wilhite (2015). Drugs that affect lipid metabolism. Side Effects of Drugs Annual, Elsevier. 37: 559-565.	Title
75	Bell, T. J. and E. E. Wright (2010). "Can Therapies That Target the Incretin System Improve Our Ability to Treat Type 2 Diabetes?" Journal of the National Medical Association 102(6): 511-525.	Title
76	Berton, M., et al. (2023). "Physiologically Based Pharmacokinetic Modelling to Identify Physiological and Drug Parameters Driving Pharmacokinetics in Obese Individuals." Clinical Pharmacokinetics 62(2): 277-295.	Full-Length Text
77	Bhatt, D., et al. (2014). "Investigating the role of plasma glucose concentration as a phenotypic marker for CYP2C9 genetic variants, in the diabetic population of Gujarat." Indian Journal of Pharmaceutical Sciences 76(1): 72.	Title
78	Bhaumik, U., et al. (2009). "Development and validation of a high-performance liquid chromatographic method for bioanalytical application with rimonabant." Journal of Pharmaceutical and Biomedical Analysis 49(4): 1009-1013.	Title
79	Bin Jardan, Y. A., et al. (2021). "Assessment of glibenclamide pharmacokinetics in poloxamer 407-induced hyperlipidemic rats." Saudi Pharmaceutical Journal 29(7): 719-723.	Animal
80	Bleskestad, I. H. and K. I. Birkeland (2003). "[Differences between oral antidiabetics]." Tidsskr Nor Laegeforen 123(6): 808-809.	Title
81	Blickle, J., et al. (1999). "Actualités dans les traitements du diabète de type 2. Les agents insulinosécréteurs." La Revue de médecine interne 20: 351s-359s.	Language
82	Bode, B. W., et al. (2011). "Comparison of the Efficacy and Tolerability Profile of Liraglutide, a Once-Daily Human GLP-1 Analog, in Patients With Type 2 Diabetes ≥65 and <65 Years of Age: A Pooled Analysis from Phase III Studies." The American Journal of Geriatric Pharmacotherapy 9(6): 423-433.	Title
83	Bogusz, M. J. and A. Carracedo (2004). Chapter 23 Forensic analysis. Journal of Chromatography Library. E. Heftmann, Elsevier. 69: 1073-1133.	Title
84	Böhm, R., et al. (2009). "[Hypoglycemic risk of insulinotropic drugs]." Med Monatsschr Pharm 32(12): 453-458.	Title
85	Bonfilio, R., et al. (2010). "A review of analytical techniques for determination of glimepiride: present and perspectives." Therapeutic drug monitoring 32(5): 550-559.	Review
86	Bongioanni, A., et al. (2022). "Amino acids and its pharmaceutical applications: A mini review." International Journal of Pharmaceutics 613: 121375.	Review
87	Borges, N. C., et al. (2007). "[Comparison study of two glimepiride formulations bioavailability in healthy volunteers of both sexes after a single dose administration]." Arq Bras Endocrinol Metabol 51(6): 950-955.	Title
88	Borobia, A. M., et al. (2009). "CYP2C9 polymorphism in five autochthonous population of the same geographic area (Spanish Pyrenees)." Pharmacological research 59(2): 107-111.	Title
89	Bose, S., et al. (2021). "Comparative in vitro evaluation of glimepiride containing nanosuspension drug delivery system developed by different techniques." Journal of Molecular Structure 1231: 129927.	TItle
90	Bouazza, N., et al. (2016). "Evaluation of the pharmacokinetics of glibenclamide tablet given, off label, orally to children suffering from neonatal syndromic hyperglycemia." European journal of clinical pharmacology 72: 1373-1379.	Title
91	Bouchghoul, H. (2021). Déterminants de l’hypoglycémie néonatale et maternelle chez les femmes ayant un diabète gestationnel traité par glyburide, Université Paris-Saclay.	Language
92	Bozkurt, Ö., et al. (2007). "Pharmacogenetics of glucose-lowering drug treatment: a systematic review." Molecular diagnosis & therapy 11: 291-302.	Review

93	Briscoe, V. J., et al. (2010). "The role of glimepiride in the treatment of type 2 diabetes mellitus." Expert Opinion on Drug Metabolism & Toxicology 6(2): 225-235.	Review
94	Brodeur, M. R. and A. L. Stirling (2001). "Delirium associated with zolpidem." Annals of Pharmacotherapy 35(12): 1562-1564.	Title
95	Brunetti, A. (2012). "Pharmacogenetics in type 2 diabetes." Clinical Management Issues 6(4): 119-125.	Review
96	Brunetti, A., et al. (2014). "Pharmacogenetics of type 2 diabetes mellitus: An example of success in clinical and translational medicine." World Journal of Translational Medicine 3(3): 141-149.	Review
97	Burger, D., et al. (2013). "Clinical management of drug–drug interactions in HCV therapy: Challenges and solutions." Journal of Hepatology 58(4): 792-800.	Title
98	Buse, J. (2000). "Combining insulin and oral agents." The American Journal of Medicine 108(6, Supplement 1): 23-32.	Title
99	Cabaleiro, T., et al. (2013). "Evaluation of the relationship between sex, polymorphisms in CYP2C8 and CYP2C9, and pharmacokinetics of angiotensin receptor blockers." Drug Metabolism and Disposition 41(1): 224-229.	Title
100	Campbell, R. K. (1998). "Glimepiride: role of a new sulfonylurea in the treatment of type 2 diabetes mellitus." Annals of pharmacotherapy 32(10): 1044-1052.	Review
101	Cascorbi, I. (2006). "Genetic basis of toxic reactions to drugs and chemicals." Toxicology Letters 162(1): 16-28.	Review
102	Castellan, A.-C., et al. (2013). "Quantitative prediction of the impact of drug interactions and genetic polymorphisms on cytochrome P450 2C9 substrate exposure." Clinical pharmacokinetics 52: 199-209.	Title
103	Celestin, M. N. and F. M. Musteata (2021). "Impact of Changes in Free Concentrations and Drug-Protein Binding on Drug Dosing Regimens in Special Populations and Disease States." Journal of Pharmaceutical Sciences 110(10): 3331-3344.	Review
104	Céspedes-Garro, C., et al. (2015). "Worldwide interethnic variability and geographical distribution of CYP2C9 genotypes and phenotypes." Expert opinion on drug metabolism & toxicology 11(12): 1893-1905.	Review
105	Chelghoum, M., et al. (2021). "New trends in the use of medicinal plants by Algerian diabetic patients, considerations of herb-drug interactions." Journal of Ethnopharmacology 274: 113984.	Review
106	Chen, S. Z., et al. (2014). "Drug-drug interaction of losartan and glimepiride metabolism by recombinant microsome CYP2C9*1, 2C9*3, 2C9*13, and 2C9*16 in vitro." Int J Clin Pharmacol Ther 52(9): 732-738.	Abstract
107	Choi, H., et al. (2014). "No pharmacokinetic drug interaction between gemigliptin and glimepiride." Clinical pharmacology and therapeutics 95: S39.	Title
108	Choi, J.-S. (2017). "RETRACTED: Enhanced stability and solubility of pH-dependent drug, telmisartan achieved by solid dispersion." Journal of Drug Delivery Science and Technology 37: 194-203.	Title
109	Chow, E., et al. (2019). "CYP2C19* 2 polymorphism is associated with impaired oral clearance of gliclazide in healthy Chinese." Pharmacogenomics and Personalized Medicine: 397-401.	Title
110	Colagiuri, S., et al. (2018). "The place of gliclazide MR in the evolving type 2 diabetes landscape: A comparison with other sulfonylureas and newer oral antihyperglycemic agents." Diabetes Res Clin Pract 143: 1-14.	Review
111	CONJUNTA, R. and B. Y. FARMACOCINÉTICA "CORDOBA, ARGENTINA."	Language
112	Connor, J. D., et al. (2005). "Single Dose Pharmacokinetics of Glimepiride (GLIM) in Pediatric Patients with Type 2 Diabetes Mellitus." Diabetes 54: A141.	Abstract

113	Constable, S. and M. Pirmohamed (2016). Pharmacogenetics and Metabolic Disease. Pharmacogenetics, CRC Press: 259-288.	Title
114	Crettol, S., et al. (2010). "Pharmacogenetics of phase I and phase II drug metabolism." Current pharmaceutical design 16(2): 204-219.	Title
115	Croom, K. F. and P. L. McCormack (2009). "Liraglutide: a review of its use in type 2 diabetes mellitus." Drugs 69(14): 1985-2004.	Review
116	Ctri (2022). "Bioavailability study of Amaryl (glimepiride) Tablet 1 mg in healthy, adult, human subjects under fasting condition." https://trialsearch.who.int/Trial2.aspx?TrialID=CTRI/2022/08/044839.	Not accessible
117	Ctri (2022). "Bioavailability study of Amaryl (glimepiride) Tablet 1 mg in healthy, adult, human subjects under fed condition." https://trialsearch.who.int/Trial2.aspx?TrialID=CTRI/2022/11/046993.	Not accessible
118	Cuautle-Rodríguez, P., et al. (2019). "Frequency of CYP2C9 (* 2,* 3 and IVS8-109A> T) allelic variants, and their clinical implications, among Mexican patients with diabetes mellitus type 2 undergoing treatment with glibenclamide and metformin." Biomedical reports 10(5): 283-295.	Title
119	Cyr, M., et al. (2002). "Increased lithium dose requirement in a hyperglycemic patient." Annals of Pharmacotherapy 36(3): 427-429.	Title
120	Dai, D., et al. (2014). "CYP2C9 polymorphism analysis in Han Chinese populations: building the largest allele frequency database." The pharmacogenomics journal 14(1): 85-92.	Title
121	DAN, H.-L. and F. YI (2011). "Effects of Gemfibrozil on several commonly used oral hypoglycemic agents." Chinese Journal of Clinical Pharmacology and Therapeutics 16(1): 116.	Title
122	Darwish, K. M., et al. (2016). "Design, synthesis, and biological evaluation of novel thiazolidinediones as PPARγ/FFAR1 dual agonists." European Journal of Medicinal Chemistry 109: 157-172.	Title
123	Das, S. K., et al. (2022). "A Study of Potential drug-drug interactions in HIV positive individuals with Co-morbidities." Research Journal of Pharmacy and Technology 15(5): 1960-1966.	Title
124	Dave, R. A. and M. E. Morris (2016). "Novel high/low solubility classification methods for new molecular entities." International Journal of Pharmaceutics 511(1): 111-126.	Title
125	Davies, B. J., et al. (2006). "Enantioselective assay for the determination of perhexiline enantiomers in human plasma by liquid chromatography." Journal of Chromatography B 832(1): 114-120.	Title
126	Dawed, A. Y., et al. (2022). Precision Medicine in Diabetes, Springer.	Title
127	Dawed, A. Y., et al. "Genome-Wide Meta-Analysis Identifies the Organic Anion-Transporting Polypeptide Gene SLCO1B1 and Statins as Modifiers of Glycemic Response to Sulfonylureas."	Title
128	Dawed, A. Y., et al. (2021). "Genome-wide meta-analysis identifies genetic variants associated with glycemic response to sulfonylureas." Diabetes Care 44(12): 2673-2682.	Title
129	Dawed, A. Y., et al. (2016). "Pharmacogenetics in type 2 diabetes: influence on response to oral hypoglycemic agents." Pharmacogenomics and personalized medicine: 17-29.	Review
130	De Nicola, L., et al. (2014). "Sodium/Glucose Cotransporter 2 Inhibitors and Prevention of Diabetic Nephropathy: Targeting the Renal Tubule in Diabetes." American Journal of Kidney Diseases 64(1): 16-24.	Review
131	Deacon, C. F. and H. E. Lebovitz (2016). "Comparative review of dipeptidyl peptidase-4 inhibitors and sulphonylureas." Diabetes Obes Metab 18(4): 333-347.	Review

132	Deb, S. and S. Arrighi (2021). "Potential effects of COVID-19 on cytochrome P450-mediated drug metabolism and disposition in infected patients." European journal of drug metabolism and pharmacokinetics 46(2): 185-203.	Title
133	Deeks, E. D. (2012). "Linagliptin: a review of its use in the management of type 2 diabetes mellitus." Drugs 72(13): 1793-1824.	Review
134	DeLuca, J., et al. (2022). "Applying Pharmacogenomic Guidelines to Combat Medical Care." Military Medicine 187(Supplement_1): 18-24.	Title
135	Demirpolat, E. and M. B. Y. Aycan (2013). "Bioequivalence and pharmacogenetics." Current pharmacogenomics and personalized medicine 11(4): 288-294.	Title
136	Denneboom, W., et al. (2008). "Drug-induced hypoglycaemia in elderly users of antidiabetic agents; incidence and risk factors." Improving medication safety in the elderly: 91.	Title
137	Deon, M., et al. (2022). "A critical review of traditional and advanced characterisation tools to drive formulators towards the rational development of 3D printed oral dosage forms." International Journal of Pharmaceutics 628: 122293.	Review
138	Derosa, G. (2007). "Pioglitazone plus glimepiride: a promising alternative in metabolic control." International Journal of Clinical Practice 61: 28-36.	Review
139	Derosa, G., et al. (2006). "Fixed-dose combination rosiglitazone/glimepiride in the treatment of Type 2 diabetes mellitus." Clinical Practice 3(5): 559.	Review
140	Derosa, G. and S. A. T. Salvadeo (2009). "Glimepiride-pioglitazone hydrochloride in the treatment of type 2 diabetes." Clinical Medicine. Therapeutics 1: CMT. S2016.	Review
141	Dholakia, J., et al. (2021). "Strategies for the delivery of antidiabetic drugs via intranasal route." International Journal of Pharmaceutics 608: 121068.	Review
142	Dhondale, M. R., et al. (2023). "Current Trends in API Co-Processing: Spherical Crystallization and Co-Precipitation Techniques." Journal of Pharmaceutical Sciences 112(8): 2010-2028.	Title
143	Dickinson, G. (2007). Propagation of pharmacogenetic differences in cytochrome P450 into pharmacokinetic and pharmacodynamic measures, University of Sheffield.	Title
144	Djelmis, J., et al. (1999). "SCIENTIFIC PAPERS." DIABETOLOGIA CROATICA 28: 1.	Title
145	Dorji, P. W., et al. (2019). "Pharmacogenetic relevant polymorphisms of CYP2C9, CYP2C19, CYP2D6, and CYP3A5 in Bhutanese population." Drug Metabolism and Personalized Therapy 34(4): 20190020.	Title
146	Dotsikas, Y., et al. (2005). "Development of a rapid method for the determination of glimepiride in human plasma using liquid-liquid extraction based on 96-well format micro-tubes and liquid chromatography/tandem mass spectrometry." Rapid Commun Mass Spectrom 19(14): 2055-2061.	Full-Length Text
147	Douros, A., et al. (2015). "Estimating kidney function and use of oral antidiabetic drugs in elderly." Fundam Clin Pharmacol 29(3): 321-328.	Abstract
148	Dsilva, L. C., et al. (2013). "Effect of food on the absorption of metformin from sustained release metformin hydrochloride formulations in healthy Indian volunteers." Asian journal of pharmaceutical and clinical research 6(1): 95-99.	Title
149	Durga Prasad Reddy, R. and V. Sharma (2020). "Additive manufacturing in drug delivery applications: A review." International Journal of Pharmaceutics 589: 119820.	Review
150	Durkin, R. and G. Weisinger (1990). Rat preproenkephalin gene structure 5 promoter sequence determination 15 kb and analysis. Society for Neuroscience Abstracts.	Animal

151	Eckardt, K.-U., et al. (2009). "Special Issue: KDIGO Clinical Practice Guideline for the Care of Kidney Transplant Recipients." American Journal of Transplantation 9: S1-S155.	Title
152	Edwards, A. and M. H. Ensom (2012). "Pharmacokinetic effects of bariatric surgery." Annals of Pharmacotherapy 46(1): 130-136.	Title
153	Elmasry, M. S., et al. (2023). "Earth-friendly micellar UPLC technique for determination of four hypoglycemic drugs in different pharmaceutical dosage forms and spiked human plasma." BMC Chem 17(1): 74.	Title
154	Escott, G. M., et al. (2021). "Monitoring and management of hyperglycemia in patients with advanced diabetic kidney disease." Journal of Diabetes and its Complications 35(2): 107774.	Review
155	Ezuruike, U. F. and J. M. Prieto (2014). "The use of plants in the traditional management of diabetes in Nigeria: Pharmacological and toxicological considerations." Journal of Ethnopharmacology 155(2): 857-924.	Review
156	Fachi, M. M., et al. (2017). "A systematic and critical review on bioanalytical method validation using the example of simultaneous quantitation of antidiabetic agents in blood." Journal of Chromatography B 1055-1056: 61-71.	Review
157	Fanning, K. J., et al. (2009). "Sulphonylurea physicochemical-pharmacokinetic relationships in the pancreas and liver." Journal of Pharmaceutical Sciences 98(8): 2807-2821.	Title
158	Fediuk, D. J., et al. (2020). "Overview of the Clinical Pharmacology of Ertugliflozin, a Novel Sodium-Glucose Cotransporter 2 (SGLT2) Inhibitor." Clin Pharmacokinet 59(8): 949-965.	Review
159	Ferreira, G. S., et al. (2020). "Comparison of drug efficacy in two animal models of type 2 diabetes: A systematic review and meta-analysis." European Journal of Pharmacology 879: 173153.	Animal
160	Forero-Schwanhaeuser, S., et al. (2016). "Glycaemic lowering with albiglutide: effective at 1 week and efficacy maintained for 1 year." Diabetologia 59(1): S379-S380.	Title
161	Forst, T., et al. (2013). "Effect of vildagliptin compared to glimepiride on postprandial proinsulin processing in the β cell of patients with type 2 diabetes mellitus." Diabetes Obes Metab 15(6): 576-579.	Full-Length Text
162	Forst, T., et al. (2010). "Linagliptin (BI 1356), a potent and selective DPP-4 inhibitor, is safe and efficacious in combination with metformin in patients with inadequately controlled Type 2 diabetes." Diabet Med 27(12): 1409-1419.	Title
163	Frick, A., et al. (1998). "Biopharmaceutical characterization of oral immediate release drug products. In vitro/in vivo comparison of phenoxymethylpenicillin potassium, glimepiride and levofloxacin." Eur J Pharm Biopharm 46(3): 305-311.	Abstract
164	Fukuen, S., et al. (2005). "Sulfonylurea Agents Exhibit Peroxisome Proliferator-activated Receptor γ Agonistic Activity*." Journal of Biological Chemistry 280(25): 23653-23659.	Title
165	Gagne, J. J., et al. (2016). "Switch-backs associated with generic drugs approved using product-specific determinations of therapeutic equivalence." Pharmacoepidemiol Drug Saf 25(8): 944-952.	Title
166	Gagne, J. J., et al. (2017). "Outcomes Associated with Generic Drugs Approved Using Product-Specific Determinations of Therapeutic Equivalence." Drugs 77(4): 427-433.	Title
167	Galatage, S. T., et al. (2022). "Oral self-emulsifying nanoemulsion systems for enhancing dissolution, bioavailability and anticancer effects of camptothecin." Journal of Drug Delivery Science and Technology 78: 103929.	Title

168	Ganesh, T., et al. (2016). "Investigation of molecular interaction between cefpodoxime acid and human mixtard insulin by ultrasonic and spectral methods." Journal of Pharmaceutical and Biomedical Analysis 129: 237-245.	Title
169	Garber, S. M., et al. (2009). "Hypoglycemia associated with the use of levofloxacin [corrected][published erratum appears in AM J HEALTH SYST PHARM AJHP 2009 Jul 15; 66 (14): 1247]." American Journal of Health-System Pharmacy 66(11).	Title
170	García-Arieta, A., et al. (2015). "On the Biopharmaceutics Classification System Biowaiver of Ibuprofen." Journal of Pharmaceutical Sciences 104(8): 2429-2432.	Title
171	George, R. E. and S. Joseph (2014). "A review of newer treatment approaches for type-2 diabetes: Focusing safety and efficacy of incretin based therapy." Saudi Pharmaceutical Journal 22(5): 403-410.	Review
172	Ghoghari, A., et al. (2016). "Quantitative determination of saroglitazar, a predominantly PPAR alpha agonist, in human plasma by a LC-MS/MS method utilizing electrospray ionization in a positive mode." Biomed Chromatogr 30(12): 1900-1907.	Title
173	Gilroy, C. A., et al. (2016). "Controlled release of biologics for the treatment of type 2 diabetes." Journal of Controlled Release 240: 151-164.	Title
174	Glintborg, B., et al. (2007). "Are patients reliable when self-reporting medication use? Validation of structured drug interviews and home visits by drug analysis and prescription data in acutely hospitalized patients." J Clin Pharmacol 47(11): 1440-1449.	Title
175	Gökalp, O., et al. (2011). "Mild hypoglycaemic attacks induced by sulphonylureas related to CYP2C9, CYP2C19 and CYP2C8 polymorphisms in routine clinical setting." European journal of clinical pharmacology 67: 1223-1229.	Title
176	Gomis, R., et al. (2000). "Appropriate timing of glimepiride administration in patients with type 2 diabetes millitus: a study in Mediterranean countries." Endocrine 13: 117-121.	Abstract
177	Gong, L., et al. (2017). "Polymorphisms in cytochrome P450 oxidoreductase and its effect on drug metabolism and efficacy." Pharmacogenetics and genomics 27(9): 337-346.	Review
178	GONZALEZ, N. P., et al. "FRECUENCIAS ALELICAS Y GENOTIPICAS DE POLIMORFISMOS EN LOS GENES CYP2C9, VKORC1 Y CYP4F2 EN PACIENTES COLOMBIANOS ANTICOAGULADOS CON WARFARINA."	Language
179	Gonzalez, O., et al. (2011). "Development of an LC–MS/MS method for the quantitation of 55 compounds prescribed in combined cardiovascular therapy." Journal of Chromatography B 879(3): 243-252.	Title
180	Gorodetskaya, G., et al. (2017). "Pharmacogenetic testing in the treatment of type 2 diabetes with sulfonylurea drugs." Bulletin of the Scientific Centre for Expert Evaluation of Medicinal Products. Regulatory Research and Medicine Evaluation 7(4): 233-241.	Review
181	Greupink, R., et al. (2013). "Semi-mechanistic physiologically-based pharmacokinetic modeling of clinical glibenclamide pharmacokinetics and drug–drug-interactions." European Journal of Pharmaceutical Sciences 49(5): 819-828.	Title
182	Grimm, M., et al. (2023). "Comparing the gastric emptying of 240 mL and 20 mL water by MRI and caffeine salivary tracer technique." European Journal of Pharmaceutics and Biopharmaceutics 184: 150-158.	Title
183	Grzeszczak, W. (2001). "Poprawa skuteczności leczenia chorych na cukrzycę typu 2 po wprowadzeniu glipizydu w formie wolno uwalnianej—Glibenese GITS." Diabetologia Praktyczna 2(3): 175-181.	Language
184	Grzeszczak, W. (2001). "Type 2 diabetes treatment improvement after introduction of extended release tablets of glipizide, Glibenese GITS." Clinical Diabetology 2(3): 175-182.	Title

185	Gupta, A. K., et al. (1999). "Drug interactions with itraconazole, fluconazole, and terbinafine and their management." Journal of the American Academy of Dermatology 41(2): 237-249.	Title
186	Gupta, S. and V. Jhawat (2017). "Quality by design (QbD) approach of pharmacogenomics in drug designing and formulation development for optimization of drug delivery systems." Journal of Controlled Release 245: 15-26.	Title
187	Hahr, A. J. and M. E. Molitch (2022). "Management of Diabetes Mellitus in Patients With CKD: Core Curriculum 2022." American Journal of Kidney Diseases 79(5): 728-736.	Title
188	Hall, R. G., et al. (2013). "Dosing considerations for obese patients receiving cancer chemotherapeutic agents." Annals of Pharmacotherapy 47(12): 1666-1674.	Review
189	Hallifax, D., et al. (2012). "Clearance-dependent underprediction of in vivo intrinsic clearance from human hepatocytes: Comparison with permeabilities from artificial membrane (PAMPA) assay, in silico and caco-2 assay, for 65 drugs." European Journal of Pharmaceutical Sciences 45(5): 570-574.	Title
190	Han, D.-G., et al. (2019). "A novel high-performance liquid chromatographic method combined with fluorescence detection for determination of ertugliflozin in rat plasma: Assessment of pharmacokinetic drug interaction potential of ertugliflozin with mefenamic acid and ketoconazole." Journal of Chromatography B 1122-1123: 49-57.	Animal
191	Han, X., et al. (2017). "Biomedical Informatics Approaches to Identifying Drug-Drug Interactions: Application to Insulin Secretagogues." Epidemiology 28(3): 459-468.	Title
192	Hanley, M. J., et al. (2010). "Effect of obesity on the pharmacokinetics of drugs in humans." Clinical pharmacokinetics 49: 71-87.	Review
193	Hanna, M., et al. (2008). "Prolonged-release oxycodone enhances the effects of existing gabapentin therapy in painful diabetic neuropathy patients." European Journal of Pain 12(6): 804-813.	Title
194	Hartmann, S. N., et al. (2004). "Lumiracoxib does not affect methotrexate pharmacokinetics in rheumatoid arthritis patients." Annals of Pharmacotherapy 38(10): 1582-1587.	Title
195	He, L., et al. (2017). "Comparative study on the interaction between 3 CYP2C9 allelic isoforms and benzbromarone by using LC–MS/MS method." Journal of Chromatography B 1070: 97-103.	Title
196	He, Y. L., et al. (2013). "Differential effects of vildagliptin and glimepiride on glucose fluctuations in patients with type 2 diabetes mellitus assessed using continuous glucose monitoring." Diabetes Obes Metab 15(12): 1111-1119.	Abstract
197	Hebert, M. F. (2013). "Impact of pregnancy on maternal pharmacokinetics of medications." Clinical pharmacology during pregnancy: 17-39.	Title
198	Hefnawy, M. M., et al. (2012). "Multi-objective optimization strategy based on desirability functions used for electrophoratic separation and quantification of rosiglitazone and glimepiride in plasma and formulations." Drug Test Anal 4(1): 39-47.	Full-Length Text
199	Heidenreich, P. A., et al. (2022). "2022 ACC/AHA/HFSA Guideline for the Management of Heart Failure." Journal of Cardiac Failure 28(5): e1-e167.	Title
200	Hillman, M. A., et al. (2004). "Relative impact of covariates in prescribing warfarin according to CYP2C9 genotype." Pharmacogenetics and Genomics 14(8): 539-547.	Title
201	Hiroi, S., et al. (2012). "Bioequivalence of a pioglitazone-glimepiride combination tablet versus coadministered single-dose pioglitazone and glimepiride in healthy Japanese subjects." Diabetes management (London, England) 2(5 SUPPL.): 21-28.	Full-Length Text

202	Hirota, T., et al. (2013). "Impact of genetic polymorphisms in CYP2C9 and CYP2C19 on the pharmacokinetics of clinically used drugs." Drug metabolism and pharmacokinetics 28(1): 28-37.	Review
203	Hizel, C., et al. (2017). Introduction: Every individual is different and precision medicine offers options for disease control and treatment. Progress and Challenges in Precision Medicine, Elsevier: 1-34.	Title
204	Hocking, S. L. "Systematic review of the association between inherited genetic variants and response to blood glucose lowering therapies."	Review
205	Hohendorff, J., et al. (2012). "Lack of Association between Arg144Cys Variant of CYP2C9 Gene and Therapeutic Response to Oral Agents in Type 2 Diabetes Patients." International Journal of Human Genetics 12(2): 83-86.	Title
206	Holstein, A., et al. (2012). "CYP2C metabolism of oral antidiabetic drugs--impact on pharmacokinetics, drug interactions and pharmacogenetic aspects." Expert Opin Drug Metab Toxicol 8(12): 1549-1563.	Review
207	Holstein, A. and E.-H. Egberts (2003). "Risk of hypoglycaemia with oral antidiabetic agents in patients with Type 2 diabetes." Experimental and clinical endocrinology & diabetes 111(07): 405-414.	Review
208	Holstein, A., et al. (2011). "Impact of clinical factors and CYP2C9 variants for the risk of severe sulfonylurea-induced hypoglycemia." European journal of clinical pharmacology 67: 471-476.	Title
209	Holstein, A., et al. (2010). "Severe sulfonylurea-induced hypoglycemia: a problem of uncritical prescription and deficiencies of diabetes care in geriatric patients." Expert opinion on drug safety 9(5): 675-681.	Review
210	Holstein, A., et al. (2011). "Therapy with oral antidiabetic drugs: applied pharmacogenetics." The British Journal of Diabetes & Vascular Disease 11(1): 10-16.	Review
211	Horie, T., et al. (2021). "Severe hypoglycaemia under abemaciclib administration in a patient with breast cancer: A case report." Molecular and Clinical Oncology 14(3): 1-1.	Title
212	Horton, E. S. (2009). "Defining the Role of Basal and Prandial Insulin for Optimal Glycemic Control." Journal of the American College of Cardiology 53(5, Supplement): S21-S27.	Title
213	Hu, C., et al. (2022). "A Sensitive HPLC-MS/MS Method for Determination of Obeticholic Acid in Human Plasma: Application to a Pharmacokinetic Study in Healthy Volunteers." J Chromatogr Sci 60(6): 545-550.	Title
214	Hu, L., et al. (2012). "Pharmacogenetics of P450 oxidoreductase: implications in drug metabolism and therapy." Pharmacogenetics and genomics 22(11): 812-819.	Title
215	Hu, S. (2002). "Interaction of nateglinide with KATP channel in β-cells underlies its unique insulinotropic action." European Journal of Pharmacology 442(1): 163-171.	Title
216	Hu, S., et al. (2003). "The mechanisms underlying the unique pharmacodynamics of nateglinide." Diabetologia 46 Suppl 1: M37-43.	Review
217	Huang, C. and J. C. Florez (2011). "Pharmacogenetics in type 2 diabetes: potential implications for clinical practice." Genome Medicine 3: 1-9.	Review
218	Huang, P.-J., et al. (2021). "Pharmacogenetic perspectives in improving pharmacokinetic profiles for efficient bioequivalence trials with highly variable drugs: A review." International journal of Pharmacokinetics 5(1): IPK02.	Review

219	Huang, Q. and Z.-q. Liu (2011). Pharmacogenetics for T2DM and Anti-Diabetic Drugs. Recent Advances in the Pathogenesis, Prevention and Management of Type 2 Diabetes and its Complications, IntechOpen.	Title
220	HUANG, Z.-j., et al. (2007). "Recent advance in association between genetic polymorphisms in CYP2C9 and pharmacokinetics and pharmacodynamics of sulphonylureas." Chinese Journal of Clinical Pharmacology and Therapeutics 12(2): 219.	Title
221	Hurren, K. M. and N. R. Pinelli (2012). "Drug-drug interactions with glucagon-like peptide-1 receptor agonists." Annals of Pharmacotherapy 46(5): 710-717.	Review
222	Hussain, H. R., et al. (2022). "Fenugreek seed mucilage grafted poly methacrylate pH-responsive hydrogel: A promising tool to enhance the oral bioavailability of methotrexate." International Journal of Biological Macromolecules 202: 332-344.	Title
223	Hussar, D. A. (2002). "New Drugs of 2001." Journal of the American Pharmaceutical Association (1996) 42(2): 227-266.	Review
224	Hussar, D. A. and J. Samuel (2011). "Vilazodone hydrochloride, linagliptin, and alcaftadine." Journal of the American Pharmacists Association 51(4): 557-559.	Title
225	Hussar, D. A. and S. Yenner (2014). "Apremilast, albiglutide, and empagliflozin." Journal of the American Pharmacists Association 54(5): 562-567.	Title
226	Hydrie, M. Z. I., et al. (2006). "Glimepiride study on type-2 diabetic subjects." Pakistan Journal of Medical Sciences 22(2): 132.	Title
227	Ibrahim, H., et al. (2016). "Mining association patterns of drug-interactions using post marketing FDA's spontaneous reporting data." Journal of Biomedical Informatics 60: 294-308.	Title
228	Imawaka, H., et al. (2012). "Inclusion of Human Intravenous Study Data in New Drug Applications—Impact of the Japanese Guidance." Journal of Pharmaceutical Sciences 101(7): 2557-2563.	Title
229	Inoue, K., et al. (2003). "Less frequent body weight gain in elderly type 2 diabetic patients treated with glimepiride." Geriatrics & Gerontology International 3(1): 56-59.	Abstract
230	Iqbal, T., et al. (2004). "Validation of a simplified method for determination of cimetidine in human plasma and urine by liquid chromatography with ultraviolet detection." Journal of Chromatography B 799(2): 337-341.	Title
231	Izutsu, K.-i., et al. (2020). "Approaches to supply bioequivalent oral solid pharmaceutical formulations through the lifecycles of products: Four-media dissolution monitoring program in Japan." Journal of Drug Delivery Science and Technology 56: 101378.	Title
232	Jain, D. S., et al. (2006). "Liquid chromatography-tandem mass spectrometry validated method for the estimation of indapamide in human whole blood." J Chromatogr B Analyt Technol Biomed Life Sci 834(1-2): 149-154.	Title
233	Jain, S., et al. (2022). "Enhanced stability and oral bioavailability of erlotinib by solid self nano emulsifying drug delivery systems." International Journal of Pharmaceutics 622: 121852.	Title
234	Jan, A., et al. (2023). "Association of CYP2C9* 2 Allele with Sulphonylurea-Induced Hypoglycaemia in Type 2 Diabetes Mellitus Patients: A Pharmacogenetic Study in Pakistani Pashtun Population." Biomedicines 11(8): 2282.	Title
235	Jarrar, Y. B. and S.-J. Lee (2014). "Molecular functionality of CYP2C9 polymorphisms and their influence on drug therapy." Drug metabolism and drug interactions 29(4): 211-220.	Review
236	Jin, T., et al. (2016). "Genetic polymorphisms analysis of drug-metabolizing enzyme CYP2C9 in the Uyghur population." Xenobiotica 46(8): 709-714.	Title

237	Jirovský, D., et al. (2010). "Electrochemical characterization of repaglinide and its determination in human plasma using liquid chromatography with dual-channel coulometric detection." Journal of Chromatography B 878(31): 3243-3248.	Title
238	Ju, G., et al. (2020). "Evaluation of Bioequivalency and Pharmacokinetic Parameters for Two Formulations of Glimepiride 1-mg in Chinese Subjects." Drug Design, Development and Therapy: 2637-2644.	Full-Length Text
239	Kadokura, T., et al. (2014). "Clinical pharmacokinetics and pharmacodynamics of the novel SGLT2 inhibitor ipragliflozin." Clin Pharmacokinet 53(11): 975-988.	Review
240	Kaku, K. (2013). "Fasiglifam as a new potential treatment option for patients with type 2 diabetes." Expert Opin Pharmacother 14(18): 2591-2600.	Review
241	Kalra, S., et al. (2013). "Usage pattern, glycemic improvement, hypoglycemia, and body mass index changes with sulfonylureas in real-life clinical practice: results from OBSTACLE Hypoglycemia Study." Diabetes Technol Ther 15(2): 129-135.	Title
242	Kang, P., et al. (2023). "Effects of CYP2C9 and CYP2C19 genetic polymorphisms on the pharmacokinetics and pharmacodynamics of gliclazide in healthy subjects." Archives of Pharmacal Research: 1-10.	Title
243	Karami, F., et al. (2019). "Analytical methodologies for determination of methotrexate and its metabolites in pharmaceutical, biological and environmental samples." Journal of Pharmaceutical Analysis 9(6): 373-391.	Review
244	Karkhaneh, L., et al. (2022). "Pharmacogenomics of sulfonylureas in type 2 diabetes mellitus; a systematic review." Journal of Diabetes & Metabolic Disorders 21(1): 863-879.	Review
245	Kasahara, N., et al. (2016). "A Pharmacokinetic/Pharmacodynamic Drug-Drug Interaction Study of Tofogliflozin (a New SGLT2 Inhibitor) and Selected Anti-Type 2 Diabetes Mellitus Drugs." Drug Res (Stuttg) 66(2): 74-81.	Title
246	Kavvoura, F., et al. (2014). "The genetic architecture of type 2 diabetes pharmacotherapy: the emerging genomic evidence." Current Pharmaceutical Design 20(22): 3610-3619.	Review
247	Keating, G. M. (2010). "Vildagliptin: a review of its use in type 2 diabetes mellitus." Drugs 70(16): 2089-2112.	Review
248	Kelani, K. M., et al. (2019). "Determination of pioglitazone, its metabolite and alogliptin in human plasma by a novel LC-MS/MS method; application to a pharmacokinetic study." Journal of Chromatography B 1132: 121803.	Title
249	Kerru, N., et al. (2018). "Current anti-diabetic agents and their molecular targets: A review." European Journal of Medicinal Chemistry 152: 436-488.	Review
250	Kheniser, K. G. and S. R. Kashyap (2018). "Diabetes management before, during, and after bariatric and metabolic surgery." Journal of Diabetes and its Complications 32(9): 870-875.	Title
251	Khursheed, R., et al. (2019). "Treatment strategies against diabetes: Success so far and challenges ahead." European Journal of Pharmacology 862: 172625.	Review
252	Kim, E., et al. (2016). "Modeling of aceclofenac metabolism to major metabolites in healthy volunteers." Drug Metabolism and Pharmacokinetics 31(6): 458-463.	Title
253	Kim, J. Y., et al. (2012). "Investigation of an active film coating to prepare new fixed-dose combination tablets for treatment of diabetes." Int J Pharm 427(2): 201-208.	Title

254	Kim, K.-A., et al. (2014). "Multiplex pyrosequencing method to determine CYP2C9* 3, VKORC1* 2, and CYP4F2* 3 polymorphisms simultaneously: its application to a Korean population and comparisons with other ethnic groups." Molecular biology reports 41: 7305-7312.	Title
255	Kim, K. P., et al. (2012). "Pharmacokinetics of a fixed-dose glimepiride/sustained-release metformin combination." Int J Clin Pharmacol Ther 50(2): 142-149.	Title
256	Kim, N. K., et al. (2009). "Substrate-dependent functional alterations of seven CYP2C9 variants."	Title
257	Kim, N.-T., et al. (2022). "Effects of CYP2C9* 3 and* 13 alleles on the pharmacokinetics and pharmacodynamics of glipizide in healthy Korean subjects." Archives of Pharmacal Research 45(2): 114-121.	Title
258	Kim, Y. M., et al. (2007). "Identifying drugs needing pharmacogenetic monitoring in a Korean hospital." American journal of health-system pharmacy 64(2): 166-175.	Title
259	Kirchheiner, J. (2004). "Arzneitherapieempfehlungen auf pharmakogenetischer Basis."	Language
260	Kirchheiner, J., et al. (2005). "Effect of genetic polymorphisms in cytochrome p450 (CYP) 2C9 and CYP2C8 on the pharmacokinetics of oral antidiabetic drugs: clinical relevance." Clinical pharmacokinetics 44: 1209-1225.	Review
261	Kirchheiner, J., et al. (2004). "The CYP2C9 polymorphism: from enzyme kinetics to clinical dose recommendations." Personalized medicine 1(1): 63-84.	Title
262	Kirienkova, E. V. e., et al. (2015). "The postprandial dynamics of gastroduodenal zone hormones in patients with metabolic obesity associated or not associated with type 2 diabetes." Diabetes mellitus 18(4): 22-27.	Title
263	Kirk, D. and C. Misita (2013). "Spuriously elevated testosterone measurements caused by application of testosterone gel at or near the phlebotomy site." Annals of Pharmacotherapy 47(1): e5-e5.	Title
264	Kishore Kumar, H., et al. (2012). "Simultaneous determination of atorvastatin and glimepiride by LC-MS/MS in human plasma and its application to a pharmacokinetic study." American Journal of Analytical Chemistry 2012.	Full-Length Text
265	Klatt, S., et al. (2013). "The influence of oral antidiabetic drugs on cellular drug uptake mediated by hepatic OATP family members." Basic Clin Pharmacol Toxicol 112(4): 244-250.	Title
266	Klen, J., et al. (2014). "CYP2C9, KCNJ11 and ABCC8 polymorphisms and the response to sulphonylurea treatment in type 2 diabetes patients." European journal of clinical pharmacology 70: 421-428.	Title
267	Koeber, R., et al. (2012). "Implementation of a cost-effective HPLC/UV-approach for medical routine quantification of donepezil in human serum." Journal of Chromatography B 881-882: 1-11.	Title
268	Kojro, G., et al. (2019). "Matrix effect screening for cloud-point extraction combined with liquid chromatography coupled to mass spectrometry: Bioanalysis of pharmaceuticals." Journal of Chromatography A 1591: 44-54.	Title
269	Kononenko, I. V., et al. (2015). "Pharmacogenetics of hypoglycemic agents." Diabetes mellitus 18(4): 28-34.	Title
270	Kovacs, P. and E. Pearson (2016). "Pharmacogenetics of sulfonylureas." The Genetics of Type 2 Diabetes and Related Traits: Biology, Physiology and Translation: 483-497.	Title
271	Kudaravalli, J., et al. (2013). "Safety and efficacy of sulfonylurea drugs in type 2 diabetes mellitus." Apollo Medicine 10(2): 165-168.	Abstract

272	Kumar, V., et al. (2008). "Differential genotype dependent inhibition of CYP2C9 in humans." Drug metabolism and disposition 36(7): 1242-1248.	Title
273	Kundlik, M. L., et al. (2012). "Rapid and specific approach for direct measurement of glimepiride in human plasma by LC-ESI-MS-MS employing automated 96 well format: application to a bioequivalence study." J Chromatogr Sci 50(1): 64-70.	Abstract
274	Kuritzky, L. (2010). "Managing Type 2 Diabetes in the Primary Care Setting: Beyond Glucocentricity." The American Journal of the Medical Sciences 340(2): 133-143.	Review
275	Landgraf, R. (2000). "Meglitinide analogues in the treatment of type 2 diabetes mellitus." Drugs Aging 17(5): 411-425.	Review
276	Lang, V. and P. E. Light (2010). "The molecular mechanisms and pharmacotherapy of ATP-sensitive potassium channel gene mutations underlying neonatal diabetes." Pharmacogenomics and personalized medicine: 145-161.	Title
277	Lang, V. Y., et al. (2012). "Pharmacogenomic analysis of ATP-sensitive potassium channels coexpressing the common type 2 diabetes risk variants E23K and S1369A." Pharmacogenet Genomics 22(3): 206-214.	Title
278	Langtry, H. D. and J. A. Balfour (1998). "Glimepiride: a review of its use in the management of type 2 diabetes mellitus." Drugs 55: 563-584.	Review
279	Lau, D. C. W. and H. Teoh (2015). "Impact of Current and Emerging Glucose-Lowering Drugs on Body Weight in Type 2 Diabetes." Canadian Journal of Diabetes 39: S148-S154.	Review
280	Laursen, T., et al. (1993). Subcutaneous absorption kinetics of two highly concentrated preparations of recombinant human growth hormone, SAGE Publications.	Title
281	Lee, H.-I., et al. (2014). "Response to Suarez-Kurtz's comments on strongly increased exposure of meloxicam in CYP2C9* 3/* 3 individuals." Pharmacogenetics and Genomics 24(8): 407-408.	Title
282	Lee, H.-I., et al. (2014). "Strongly increased exposure of meloxicam in CYP2C9* 3/* 3 individuals." Pharmacogenetics and Genomics 24(2): 113-117.	Title
283	Lee, S. H., et al. (2016). "Omarigliptin, a once-weekly DPP-4 inhibitor, improves glycemic control in patients inadequately controlled on the combination of metformin and glimepiride." Diabetes 65: A50.	Title
284	Lee, S.-J. and J.-G. Shin (2014). "The pharmacogenomics of cytochrome P450s: from molecular to clinical application." Fifty years of cytochrome P450 research: 345-370.	Title
285	León, D. D. D., et al. (2006). "Role of glucagon-like peptide-1 in the pathogenesis and treatment of diabetes mellitus." The International Journal of Biochemistry & Cell Biology 38(5): 845-859.	Review
286	Li, C., et al. (2014). "Direct comparison of two albumin-based paclitaxel-loaded nanoparticle formulations: Is the crosslinked version more advantageous?" International Journal of Pharmaceutics 468(1): 15-25.	Title
287	Li, G., et al. (2022). "The drug interaction potential of berberine hydrochloride when co-administered with simvastatin, fenofibrate, gemfibrozil, metformin, glimepiride, nateglinide, pioglitazone and sitagliptin in beagles." Arabian Journal of Chemistry 15(2): 103562.	Animal
288	Li, J., et al. (2015). "Prediction of drug disposition in diabetic patients by means of a physiologically based pharmacokinetic model." Clin Pharmacokinet 54(2): 179-193.	Title
289	Li, N., et al. (2018). "Recent progress of the development of dipeptidyl peptidase-4 inhibitors for the treatment of type 2 diabetes mellitus." European Journal of Medicinal Chemistry 151: 145-157.	Review

290	Li, Q., et al. (2017). "Polymorphisms of the KCNQ1 gene are associated with the therapeutic responses of sulfonylureas in Chinese patients with type 2 diabetes." Acta Pharmacologica Sinica 38(1): 80-89.	Abstract
291	Li, R., et al. (2017). "Transporter-mediated disposition, clinical pharmacokinetics and cholestatic potential of glyburide and its primary active metabolites." Drug Metabolism and Disposition 45(7): 737-747.	Title
292	Li, W., et al. (2023). "Research progress on classification, sources and functions of dietary polyphenols for prevention and treatment of chronic diseases." Journal of Future Foods 3(4): 289-305.	Title
293	Li, W. L., et al. (2004). "Natural medicines used in the traditional Chinese medical system for therapy of diabetes mellitus." Journal of Ethnopharmacology 92(1): 1-21.	Review
294	Li, X., et al. (2022). "Drug-induced liver injury in COVID-19 treatment: Incidence, mechanisms and clinical management." Frontiers in Pharmacology 13: 1019487.	Title
295	LI, Y., et al. (2012). "Effect of CYP2C9, CYP2C19, CYP3A4 polymorphism on metabolism of sulfonyluea antidiabetic drugs." Chinese Journal of Clinical Pharmacology and Therapeutics 17(5): 582.	Title
296	Li, Y., et al. (2023). "Milk-derived exosomes as a promising vehicle for oral delivery of hydrophilic biomacromolecule drugs." Asian Journal of Pharmaceutical Sciences 18(2): 100797.	Title
297	Li, Z., et al. (2017). "Discovery of phenylsulfonyl acetic acid derivatives with improved efficacy and safety as potent free fatty acid receptor 1 agonists for the treatment of type 2 diabetes." European Journal of Medicinal Chemistry 138: 458-479.	Title
298	Liao, J., et al. (2023). "Recent advances in biomimetic nanodelivery systems: New brain-targeting strategies." Journal of Controlled Release 358: 439-464.	Title
299	Ligtenberg, J. J. M., et al. (1995). "Clinical relevance of ATP-dependent potassium channels." The Netherlands Journal of Medicine 47(5): 241-251.	Title
300	Lim, Y.-J., et al. (2014). "Genetic polymorphisms of CYP2C9, CYP2C19, CYP2D6, CYP3A4, and CYP3A5 in Vietnamese-Koreans." Translational and Clinical Pharmacology 22(2): 70-77.	title
301	Lin, Y., et al. (2023). "Effects of different doses glimepiride intake on the pharmacokinetics of benzbromarone in rats." Pakistan Journal of Pharmaceutical Sciences 36(1).	Animal
302	Little, G. L. and K. S. Boniface (2005). "Are one or two dangerous? Sulfonylurea exposure in toddlers." The Journal of Emergency Medicine 28(3): 305-310.	Title
303	Liu, J., et al. (2020). "Mechanisms for oral absorption enhancement of drugs by nanocrystals." Journal of Drug Delivery Science and Technology 56: 101607.	Title
304	LIU, J. and Y.-q. XIONG "Influence of related genetic polymorphisms to pharmacokinetics and pharmacodynamics of Fluvastatin." Chinese Journal of Clinical Pharmacology and Therapeutics 17(11): 1282.	Title
305	Liu, X., et al. (2012). "Pharmacokinetics of glimepiride in normal and diabetic rats and effects of co-adminstration Huangqi injection on it." Chin. J. New Drugs Clin. Rem 31: 227-230.	Animal
306	Lloyd-Jones, D. M., et al. (2016). "2016 ACC Expert Consensus Decision Pathway on the Role of Non-Statin Therapies for LDL-Cholesterol Lowering in the Management of Atherosclerotic Cardiovascular Disease Risk: A Report of the American College of Cardiology Task Force on Clinical Expert Consensus Documents." Journal of the American College of Cardiology 68(1): 92-125.	Title

307	Loomba-Albrecht, L. A., et al. (2010). "A novel glucokinase gene mutation and its effect on glycemic/C-peptide fluctuations in a patient with maturity-onset diabetes of the young type 2." Diabetes Res Clin Pract 87(3): e23-25.	Title
308	Lopresti, A. L. and S. J. Smith (2021). "Ashwagandha (Withania somnifera) for the treatment and enhancement of mental and physical conditions: A systematic review of human trials." Journal of Herbal Medicine 28: 100434.	Review
309	Lu, S., et al. (2018). "Pharmacokinetics of CYP2C9, CYP2C19, and CYP2D6 substrates in healthy Chinese and European subjects." European Journal of Clinical Pharmacology 74: 285-296.	Title
310	Lubowsky, N. D., et al. (2007). "Management of Glycemia in Patients With Diabetes Mellitus and CKD." American Journal of Kidney Diseases 50(5): 865-879.	Title
311	Luo, Y. (2020). The interaction between glimepiride and other drugs metabolized by CYP2C9 using an in vitro model, Tufts University-Graduate School of Biomedical Sciences.	Abstract
312	Mach, M., et al. (2021). "Discovery and development of CPL207280 as new GPR40/FFA1 agonist." European Journal of Medicinal Chemistry 226: 113810.	Title
313	Macha, S., et al. (2012). "Pharmacokinetics of empagliflozin, a sodium glucose cotransporter 2 inhibitor, and glimepiride following co-administration in healthy volunteers: a randomised, open-label, crossover study." J Diabetes Res Clin Metab 1(1): 14.	Title
314	Macha, S., et al. (2010). BI 10773, A HIGHLY SELECTIVE AND POTENT SODIUM-GLUCOSE CO-TRANSPORTER-2 INHIBITOR (SGLT-2), DOES NOT ALTER THE PHARMACOKINETICS OF GLIMEPIRIDE FOLLOWING CO-ADMINISTRATION IN HEALTHY VOLUNTEERS. JOURNAL OF CLINICAL PHARMACOLOGY, SAGE PUBLICATIONS INC 2455 TELLER RD, THOUSAND OAKS, CA 91320 USA.	Not accessible
315	Madaan, T., et al. (2016). "Sodium glucose CoTransporter 2 (SGLT2) inhibitors: Current status and future perspective." European Journal of Pharmaceutical Sciences 93: 244-252.	Title
316	Maekawa, K., et al. (2009). "Substrate-dependent functional alterations of seven CYP2C9 variants found in Japanese subjects." Drug metabolism and disposition 37(9): 1895-1903.	Title
317	Maier, V., et al. (2009). "Determination of antihyperglycemic drugs in nanomolar concentration levels by micellar electrokinetic chromatography with non-ionic surfactant." Journal of Chromatography A 1216(20): 4492-4498.	Abstract
318	Maitland-van der Zee, A. and A. de Boer (2003). "Farmacogenetica: geneesmiddelentherapie aangepast aan het genotype van de patiënt?" Geneesmiddelenbulletin 37(3): 25-30.	Language
319	Maji, I., et al. (2021). "Solid self emulsifying drug delivery system: Superior mode for oral delivery of hydrophobic cargos." Journal of Controlled Release 337: 646-660.	Review
320	Mannino, G. C. and G. Sesti (2012). "Individualized therapy for type 2 diabetes: clinical implications of pharmacogenetic data." Molecular diagnosis & therapy 16: 285-302.	Title
321	Manolopoulos, V. G., et al. (2011). "Pharmacogenomics of oral antidiabetic medications: current data and pharmacoepigenomic perspective." Pharmacogenomics 12(8): 1161-1191.	Title
322	Marcath, L. A., et al. (2019). "Challenges to assess substrate-dependent allelic effects in CYP450 enzymes and the potential clinical implications." The pharmacogenomics journal 19(6): 501-515.	Title

323	Marjani, A. and A. M. Gharanjik (2018). "Genetic polymorphism of CYP2C9 among Sistani ethnic group in Gorgan." Indian Journal of Clinical Biochemistry 33: 208-213.	Title
324	Markoff, B. and A. Amsterdam (2008). "Impact of obesity on hospitalized patients." Mount Sinai Journal of Medicine: A Journal of Translational and Personalized Medicine: A Journal of Translational and Personalized Medicine 75(5): 454-459.	Title
325	Maruthur, N. M., et al. (2014). "The pharmacogenetics of type 2 diabetes: a systematic review." Diabetes Care 37(3): 876-886.	Review
326	Mary Rebecca, Y., et al. (2023). "Urinary excretion of metformin in diabetic patients with and without tuberculosis." Indian J Tuberc 70(1): 37-41.	Title
327	Masclee, A. A. and B. v. Hoek (2004). "Hepato-biliary and Pancreatic Function." Gastrointestinal Function in Diabetes Mellitus: 247-283.	Title
328	Massi-Benedetti, M. (2003). "Glimerpiride in type 2 diabetes mellitus: a review of the worldwide therapeutic experience." Clinical therapeutics 25(3): 799-816.	Review
329	Mastan, S. "The Possible Pharmacokinetic Interactions between Antiretroviral Drugs and Antidiabetic Drugs: An Overview SK. Mastan, G. Chaitanya 2, K. Raghunandan Reddy 2, 3 and K. Eswar Kumar 4."	Title
330	Mathew, A. J., et al. (2010). "Modeling of ATP-sensitive inward rectifier potassium channel 11 and inhibition mechanism of the natural ligand, ellagic acid, using molecular docking." Adv Exp Med Biol 680: 489-495.	Title
331	McCall, A. L. (2001). "Clinical review of glimepiride." Expert Opinion on Pharmacotherapy 2(4): 699-713.	Review
332	McGill, J. B. (2010). "Liraglutide: effects beyond glycaemic control in diabetes treatment." Int J Clin Pract Suppl(167): 28-34.	Review
333	Mechanick, J. I., et al. (2020). "Cardiometabolic-Based Chronic Disease, Addressing Knowledge and Clinical Practice Gaps: JACC State-of-the-Art Review." Journal of the American College of Cardiology 75(5): 539-555.	Review
334	Mégarbane, B., et al. (2022). "Glimepiride pharmacokinetics in overdose." Clin Toxicol (Phila) 60(11): 1284-1285.	Review
335	Mistri, H. N., et al. (2007). "Liquid chromatography tandem mass spectrometry method for simultaneous determination of antidiabetic drugs metformin and glyburide in human plasma." Journal of Pharmaceutical and Biomedical Analysis 45(1): 97-106.	Abstract
336	Mohsen, A. M., et al. (2017). "Enhanced oral bioavailability and sustained delivery of glimepiride via niosomal encapsulation: in-vitro characterization and in-vivo evaluation." Drug Development and Industrial Pharmacy 43(8): 1254-1264.	Animal
337	Monnier, L., et al. (2012). "Add-on therapies to metformin in type 2 diabetes: what modulates the respective decrements in postprandial and basal glucose?" Diabetes Technol Ther 14(10): 943-950.	Abstract
338	Mosikian, A., et al. (2016). "Possible approaches to CYP2C9-guided prescription of sulfonylureas in Russia." Pharmacogenomics 17(18): 2115-2126.	Title
339	Mottl, A. K., et al. (2022). "KDOQI US Commentary on the KDIGO 2020 Clinical Practice Guideline for Diabetes Management in CKD." American Journal of Kidney Diseases 79(4): 457-479.	Title
340	Muskiet, M. H. A., et al. (2022). "Postprandial renal haemodynamic effects of the dipeptidyl peptidase-4 inhibitor linagliptin versus the sulphonylurea glimepiride in adults with type 2 diabetes (RENALIS): a predefined substudy of a randomized, double-blind trial." Diabetes, obesity & metabolism 24(1): 115-124.	Abstract

341	Muskiet, M. H. A., et al. (2020). "Effects of DPP-4 Inhibitor Linagliptin Versus Sulfonylurea Glimepiride as Add-on to Metformin on Renal Physiology in Overweight Patients With Type 2 Diabetes (RENALIS): a Randomized, Double-Blind Trial." Diabetes care 43(11): 2889-2893.	Title
342	Musselman, R. C. (1998). "Monitoring the medications of clients with noninsulin-dependent diabetes mellitus." Home Care Provider 3(2): 95-99.	Title
343	Nachega, J. B., et al. (2012). "Antiretroviral therapy adherence and drug–drug interactions in the aging HIV population." Aids 26: S39-S53.	Title
344	Nakashima, M., et al. (1993). "Safety, pharmacodynamics and pharmacokinetics of glimepiride (HOE490) after single oral administration in healthy male volunteers." Rinsho Iyaku 9(3): 503-522.	Not accessible
345	Nam, Y. H., et al. (2020). "Sulfonylureas and Metformin Were Not Associated With an Increased Rate of Serious Bleeding in Warfarin Users: A Self-Controlled Case Series Study." Clin Pharmacol Ther 108(5): 1010-1017.	Title
346	Nct (2012). "Bioequivalence Study of 4 mg Glimepiride Tablet." https://clinicaltrials.gov/show/NCT01677247.	Not accessible
347	Nct (2012). "The Effects of GLP-1 in Maturity-Onset Diabetes of The Young (MODY)." https://clinicaltrials.gov/show/NCT01610934.	Title
348	Nct (2012). "Evaluation of Food Effect on the Pharmacokinetics of Sustained Release Metformin in Healthy Indian Volunteers." https://clinicaltrials.gov/show/NCT01561976.	Title
349	Nct (2015). "COMPOUND (INN): HOE490O - GLIMEPIRIDE / METFORMIN HCl (Amaryl® M)0 (Glimepiride/Metformin Hydrochloride Immediate Release Combination Tablet) in Fed Conditions in Healthy Male and/or Female Subjects." https://clinicaltrials.gov/show/NCT02395237.	Not accessible
350	Nct (2022). "Bioequivalence Study of 3 mg Glimepiride Tablet in Indonesia Healthy Subjects." https://clinicaltrials.gov/show/NCT05468879.	Not accessible
351	Neumiller, J. J. (2009). "Differential chemistry (structure), mechanism of action, and pharmacology of GLP-1 receptor agonists and DPP-4 inhibitors." Journal of the American Pharmacists Association 49(5, Supplement 1): S16-S29.	Review
352	Neumiller, J. J. and R. K. Campbell (2009). "Liraglutide: a once-daily incretin mimetic for the treatment of type 2 diabetes mellitus." Annals of pharmacotherapy 43(9): 1433-1444.	Review
353	Neumiller, J. J. and S. M. Setter (2009). "Pharmacologic management of the older patient with type 2 diabetes mellitus." The American Journal of Geriatric Pharmacotherapy 7(6): 324-342.	Review
354	Newman, D., et al. (2007). "Serum piperacillin/tazobactam pharmacokinetics in a morbidly obese individual." Annals of Pharmacotherapy 41(10): 1734-1739.	Title
355	Ng, L. C. and M. Gupta (2020). "Transdermal drug delivery systems in diabetes management: A review." Asian Journal of Pharmaceutical Sciences 15(1): 13-25.	Review
356	Nicolas, J.-M., et al. (2012). "Effect of gemfibrozil on the metabolism of brivaracetam in vitro and in human subjects." Drug Metabolism and Disposition 40(8): 1466-1472.	Title
357	Niemi, M. (2001). "Effects of induction and inhibition of Cytochrome P-450 enzymes on the pharmacokinetics and pharmacodynamics of oral antidiabetic drugs."	Title

358	Nirogi, R. V. S., et al. (2007). "A simple and rapid HPLC/UV method for the simultaneous quantification of theophylline and etofylline in human plasma." Journal of Chromatography B 848(2): 271-276.	Title
359	Nishida, H., et al. (2009). "Glimepiride Treatment Upon Reperfusion Limits Infarct Size via the phosphatidylinositol 3-Kinase/Akt Pathway in Rabbit Hearts." Journal of Pharmacological Sciences 109(2): 251-256.	Animal
360	Nix, D. E., et al. (1992). "Dose-ranging pharmacokinetic study of ciprofloxacin after 200-, 300-, and 400-mg intravenous doses." Annals of Pharmacotherapy 26(1): 8-10.	Title
361	Ntr (2013). "Amino acids in type 2 diabetes." https://trialsearch.who.int/Trial2.aspx?TrialID=NTR4181.	Title
362	Overkamp, D., et al. (2002). "Acute effect of glimepiride on insulin-stimulated glucose metabolism in glucose-tolerant insulin-resistant offspring of patients with type 2 diabetes." Diabetes Care 25(11): 2065-2073.	Abstract
363	Paixão, P., et al. (2010). "Prediction of the in vitro intrinsic clearance determined in suspensions of human hepatocytes by using artificial neural networks." European Journal of Pharmaceutical Sciences 39(5): 310-321.	Title
364	Paixão, P., et al. (2012). "Prediction of the human oral bioavailability by using in vitro and in silico drug related parameters in a physiologically based absorption model." International Journal of Pharmaceutics 429(1): 84-98.	Title
365	Palmer, K., et al. (2019). "Pharmacokinetic Study of Cefazolin in Short Daily Hemodialysis." Annals of Pharmacotherapy 53(4): 348-356.	Title
366	Pandit, S., et al. (2017). "Evaluation of herb-drug interaction of a polyherbal Ayurvedic formulation through high throughput cytochrome P450 enzyme inhibition assay." Journal of Ethnopharmacology 197: 165-172.	Title
367	Pandit, V., et al. (2012). "Bioavailability & Bioequivalence."	Title
368	Paneni, F. and T. F. Lüscher (2017). "Cardiovascular Protection in the Treatment of Type 2 Diabetes: A Review of Clinical Trial Results Across Drug Classes." The American Journal of Medicine 130(6, Supplement): S18-S29.	Review
369	Papazafiropoulou, A., et al. (2015). "Effects of oral hypoglycemic agents on platelet function." Journal of Diabetes and its Complications 29(6): 846-851.	Abstract
370	Park, H., et al. (2020). "Characterization and therapeutic efficacy evaluation of glimepiride and L-arginine co-amorphous formulation prepared by supercritical antisolvent process: Influence of molar ratio and preparation methods." Int J Pharm 581: 119232.	Abstract
371	Park, J. W., et al. (2019). "Effect of glimepiride on the pharmacokinetics of teneligliptin in healthy Korean subjects." Journal of Clinical Pharmacy and Therapeutics 44(5): 720-725.	Abstract
372	Park, Y. S., et al. (2012). "Quantification of galantamine in human plasma by validated liquid chromatography-tandem mass spectrometry using glimepride as an internal standard: application to bioavailability studies in 32 healthy Korean subjects." J Chromatogr Sci 50(9): 803-809.	Abstract
373	Pathania, S. and R. K. Rawal (2018). "Pyrrolopyrimidines: An update on recent advancements in their medicinal attributes." European Journal of Medicinal Chemistry 157: 503-526.	Review
374	Paul, A., et al. (2023). "Structural and molecular insights of protein tyrosine phosphatase 1B (PTP1B) and its inhibitors as anti-diabetic agents." Journal of Molecular Structure 1293: 136258.	Title

375	Paul, M. "Klinische Pharmakologie."	Language
376	Paz-Pacheco, E., et al. (2023). "Genetic Variants Associated with Poor Responsiveness to Sulfonylureas in Filipinos with Type 2 Diabetes Mellitus." Journal of the ASEAN Federation of Endocrine Societies 38(1): 31.	Title
377	Pearson, E. R. (2009). "Pharmacogenetics and future strategies in treating hyperglycaemia in diabetes." Frontiers in Bioscience-Landmark 14(11): 4348-4362.	Review
378	Peragallo-Dittko, V. (2007). "Matching Insulin to Patient: How to Make the Best Use of Premixed Insulin Analog Formulations." The Journal for Nurse Practitioners 3(2): 85-89.	Title
379	Pérez, M. A. C., et al. (2004). "A topological sub-structural approach for predicting human intestinal absorption of drugs." European Journal of Medicinal Chemistry 39(11): 905-916.	Title
380	Pervez, S., et al. (2023). "Transdermal Delivery of Glimepiride: A Novel Approach Using Nanomicelle-Embedded Microneedles." Pharmaceutics 15(8).	Review
381	Perwitasari, D. A., et al. (2021). "Identification of SNP rs1799853 of CYP2C9 Gene and Blood Sugar Levels In Diabetic Patients." Asian Journal of Pharmacology & Toxicology 9(1).	Title
382	Peternel, L., et al. (2012). "Suitability of Isolated Rat Jejunum Model for Demonstration of Complete Absorption in Humans for BCS-Based Biowaiver Request." Journal of Pharmaceutical Sciences 101(4): 1436-1449.	Animal
383	Pettus, J., et al. (2016). "Effect of ranolazine on glycaemic control in patients with type 2 diabetes treated with either glimepiride or metformin." Diabetes Obes Metab 18(5): 463-474.	Abstract
384	Pistos, C., et al. (2005). "Bioequivalence evaluation of two brands of glimepiride 4 mg tablets in healthy subjects." Int J Clin Pharmacol Ther 43(4): 203-208.	Not accessible
385	Plosker, G. L. (2012). "Dapagliflozin: a review of its use in type 2 diabetes mellitus." Drugs 72(17): 2289-2312.	Review
386	Plosker, G. L. and D. P. Figgitt (2004). "Repaglinide : a pharmacoeconomic review of its use in type 2 diabetes mellitus." Pharmacoeconomics 22(6): 389-411.	Review
387	Prajapati, S. K., et al. (2019). "Biodegradable polymers and constructs: A novel approach in drug delivery." European Polymer Journal 120: 109191.	Title
388	Price, R. D., et al. (2001). "A study to evaluate primary dressings for the application of cultured keratinocytes." British Journal of Plastic Surgery 54(8): 687-696.	Title
389	Profozic, V., et al. (1999). "Safety, efficacy, and pharmacokinetics of glimepiride in diabetic patients with renal impairment over a 3-month period." Diabetol Croat 28(1): 25-32.	Language
390	Profozic, V., et al. (1991). "Pharmacokinetics of glimepiride in kidney disease." Diabetes 40(1): 343A.	Not accessible
391	Puri, P. and N. Kotwal (2022). "An Approach to the Management of Diabetes Mellitus in Cirrhosis: A Primer for the Hepatologist." Journal of Clinical and Experimental Hepatology 12(2): 560-574.	Review
392	Puszkiel, A., et al. (2017). "A simple HPLC-UV method for quantification of enzalutamide and its active metabolite N-desmethyl enzalutamide in patients with metastatic castration-resistant prostate cancer." Journal of Chromatography B 1058: 102-107.	Title

393	Qiu, X., et al. (2015). "An UPLC–MS/MS method for the analysis of glimepiride and fluoxetine in human plasma." Journal of Chromatography B 980: 16-19.	Abstract
394	Que, L., et al. (2022). "No apparent pharmacokinetic interactions were found between henagliflozin: a novel sodium-glucose co-transporter 2 inhibitor and glimepiride in healthy Chinese male subjects." Journal of Clinical Pharmacy and Therapeutics 47(8): 1225-1231.	Not accessible
395	Rabbani, G. and S. N. Ahn (2019). "Structure, enzymatic activities, glycation and therapeutic potential of human serum albumin: A natural cargo." International Journal of Biological Macromolecules 123: 979-990.	Title
396	Ragia, G., et al. (2022). "SLCO1B1 c. 521T> C gene polymorphism decreases hypoglycemia risk in sulfonylurea-treated type 2 diabetic patients." Drug Metabolism and Personalized Therapy 37(4): 347-352.	Title
397	Ragia, G., et al. (2009). "Presence of CYP2C9* 3 allele increases risk for hypoglycemia in Type 2 diabetic patients treated with sulfonylureas." Pharmacogenomics 10(11): 1781-1787.	Title
398	Rajesh, S. Y., et al. (2018). "Impact of various solid carriers and spray drying on pre/post compression properties of solid SNEDDS loaded with glimepiride: in vitro-ex vivo evaluation and cytotoxicity assessment." Drug Dev Ind Pharm 44(7): 1056-1069.	Abstract
399	Ramakrishna, N. V. S., et al. (2004). "Quantitation of Valdecoxib in human plasma by high-performance liquid chromatography with ultraviolet absorbance detection using liquid–liquid extraction." Journal of Chromatography B 802(2): 271-275.	Title
400	Ramakrishna, N. V. S., et al. (2005). "Validated liquid chromatographic ultraviolet method for the quantitation of Etoricoxib in human plasma using liquid–liquid extraction." Journal of Chromatography B 816(1): 215-221.	Title
401	Ramakrishna, N. V. S., et al. (2005). "High-performance liquid chromatography method for the quantification of rabeprazole in human plasma using solid-phase extraction." Journal of Chromatography B 816(1): 209-214.	Title
402	Ramani, J., et al. (2022). "A review on the medicinal chemistry of sodium glucose co-transporter 2 inhibitors (SGLT2-I): Update from 2010 to present." European Journal of Medicinal Chemistry Reports 6: 100074.	Review
403	Raptis, S. A. and G. D. Dimitriadis (2001). "Oral hypoglycemic agents: insulin secretagogues, alpha-glucosidase inhibitors and insulin sensitizers." Exp Clin Endocrinol Diabetes 109 Suppl 2: S265-287.	Title
404	Raskin, P. and P. F. Mora (2010). "Glycaemic control with liraglutide: the phase 3 trial programme." Int J Clin Pract Suppl(167): 21-27.	Review
405	Ratheiser, K., et al. (1993). "Dose relationship of stimulated insulin production following intravenous application of glimepiride in healthy man." Arzneimittel-Forschung 43(8): 856-858.	Abstract
406	Rathod, P. and R. P. Yadav (2021). "Anti-diabesity potential of various multifunctional natural molecules." Journal of Herbal Medicine 27: 100430.	Review
407	Rebelo, A. I. C. (2016). Alterações Farmacocinéticas e Suas Implicações em Estados de Obesidade Mórbida em Adultos. Experiência Profissionalizante na Vertente de Farmácia Comunitária, Hospitalar e Investigação, Universidade da Beira Interior (Portugal).	Language
408	Reed, M. D., et al. (1991). Ceftizoxime Disposition in Neonates and Infants during the First Sex Months of Life, SAGE Publications.	Title

409	Remko, M. (2009). "Theoretical study of molecular structure, pKa, lipophilicity, solubility, absorption, and polar surface area of some hypoglycemic agents." Journal of Molecular Structure: THEOCHEM 897(1): 73-82.	Title
410	Ren, Q., et al. (2014). "Search for genetic determinants of sulfonylurea efficacy in type 2 diabetic patients from China." Diabetologia 57: 746-753.	Title
411	Ren, Q., et al. (2016). "Combined influence of genetic variants and gene-gene interaction on sulfonylurea efficacy in type 2 diabetic patients." Experimental and Clinical Endocrinology & Diabetes 124(03): 157-162.	Title
412	Ren, Q., et al. (2016). "Genetic and clinical predictive factors of sulfonylurea failure in patients with type 2 diabetes." Diabetes Technology & Therapeutics 18(9): 586-593.	Title
413	Rendell, M. (2004). "The role of sulphonylureas in the management of type 2 diabetes mellitus." Drugs 64: 1339-1358.	Review
414	Riddle, M. C. (2004). "Timely initiation of basal insulin." The American Journal of Medicine 116(3, Supplement 1): 3-9.	Title
415	Rivera-Mancía, S., et al. (2018). "Utility of curcumin for the treatment of diabetes mellitus: Evidence from preclinical and clinical studies." Journal of Nutrition & Intermediary Metabolism 14: 29-41.	Title
416	Rizzo, M. R., et al. (2005). "Repaglinide has more beneficial effect on cardiovascular risk factors than glimepiride: data from meal-test study." Diabetes Metab 31(3 Pt 1): 255-260.	Title
417	Robertson, C. (2008). "Translating ADA/EASD Guidelines and the ACE/AACE Road Maps into Primary Care of Patients with Type 2 Diabetes." The Journal for Nurse Practitioners 4(9): 661-671.	Title
418	Rolla, A. R. (2011). "Progression of Type 2 Diabetes and Insulin Initiation." Journal of the National Medical Association 103(3): 241-249.	Title
419	Rosak, C. (2002). "The pathophysiologic basis of efficacy and clinical experience with the new oral antidiabetic agents." Journal of Diabetes and its Complications 16(1): 123-132.	Abstract
420	Rosemary, J. and C. Adithan (2007). "The pharmacogenetics of CYP2C9 and CYP2C19: ethnic variation and clinical significance." Current clinical pharmacology 2(1): 93-109.	Title
421	Rosenkranz, B. (1996). "Pharmacokinetic basis for the safety of glimepiride in risk groups of NIDDM patients." Hormone and metabolic research 28(09): 434-439.	Review
422	Rosenkranz, B., et al. (1991). Pharmacokinetics of Glimepiride in Kidney-Disease. CLINICAL PHARMACOLOGY & THERAPEUTICS, Nature Publishing Group 75 VARICK ST, 9TH FLR, NEW YORK, NY 10013-1917 USA.	Review
423	Rosenstock, J. (2001). "Management of type 2 diabetes mellitus in the elderly: special considerations." Drugs Aging 18(1): 31-44.	Title
424	Rosskamp, R., et al. (1996). "Clinical profile of the novel sulphonylurea glimepiride." Diabetes Res Clin Pract 31 Suppl: S33-42.	Abstract
425	Ruikar, D. B., et al. (2022). "EXPLORING THE INHIBITION POTENTIAL OF CYP2C9 ENZYME: IN VITRO ASSESSMENT AND PREDICTION OF GLIMEPIRIDE-SULFAMETHOXAZOLE INTERACTIONS." Journal of Population Therapeutics and Clinical Pharmacology 29(04): 515-524.	Review
426	Ruikar, D. B. and S. J. Rajput "Effect of Pineapple and Pomegranate juices on CYP2C9 mediated glimepiride metabolism in vitro."	Title
427	Rustenbeck, I. (2016). "[Risk and benefit of sulfonylureas--their role in view of new treatment options for type 2 diabetes]." Med Monatsschr Pharm 39(2): 65-72; quiz 73-64.	Title

428	Ruyatkina, L. A. and M. Y. e. Sorokin (2012). "Modern antihyperglycemic therapy: safety and efficacy of glimepiride." Diabetes mellitus 15(2): 89-97.	Review
429	Saberi, M., et al. (2020). "The effect of CYP2C9 genotype variants in type 2 diabetes on the pharmacological effectiveness of sulfonylureas, diabetic retinopathy, and nephropathy." Vascular Health and Risk Management: 241-248.	Title
430	Saeidi, M., et al. (2017). "Genetic polymorphism of CYP2C9 among Sistani ethnic group in Gorgan." Annals of Medical and Health Sciences Research 7(1): 20-24.	Title
431	Salam, R. F. A., et al. (2014). "Effect of CYP2C9 gene polymorphisms on response to treatment with sulfonylureas in a cohort of Egyptian type 2 diabetes mellitus patients." Comparative Clinical Pathology 23: 341-346.	Title
432	Salerno, L., et al. (2019). "Progress in the development of selective heme oxygenase-1 inhibitors and their potential therapeutic application." European Journal of Medicinal Chemistry 167: 439-453.	Title
433	Sämann, A. and U. A. Müller (2013). "Dosage of antihyperglycemic drugs in patients with renal insufficiency." Diabetes and Kidney Disease: 186-201.	Title
434	Samer, C. F., et al. (2013). "Applications of CYP450 testing in the clinical setting." Molecular diagnosis & therapy 17: 165-184.	Review
435	Sana, T., et al. (2016). "Therapeutic effect of atorvastatin on kidney functions and urinary excretion of Glimepiride in healthy adult human male subjects." Pakistan journal of pharmaceutical sciences 29.	Full-Length Text
436	Sánchez-Pozos, K., et al. (2016). "Genetic variability of CYP2C9* 2 and CYP2C9* 3 in seven indigenous groups from Mexico." Pharmacogenomics 17(17): 1881-1889.	Title
437	Sankaralingam, S., et al. (2015). "The impact of obesity on the pharmacology of medications used for cardiovascular risk factor control." Canadian Journal of Cardiology 31(2): 167-176.	Review
438	Sarma, A., et al. (2023). "Green tea: Current trends and prospects in nutraceutical and pharmaceutical aspects." Journal of Herbal Medicine 41: 100694.	Title
439	Sasaki, T., et al. (2015). "Absence of Drug-Drug Interactions Between Luseogliflozin, a Sodium-Glucose Co-transporter-2 Inhibitor, and Various Oral Antidiabetic Drugs in Healthy Japanese Males." Adv Ther 32(5): 404-417.	Abstract
440	Satheeshkumar, N., et al. (2014). "Pioglitazone: A review of analytical methods." Journal of Pharmaceutical Analysis 4(5): 295-302.	Review
441	Sato, R., et al. (2010). "ABCC8 polymorphism (Ser1369Ala): influence on severe hypoglycemia due to sulfonylureas." Pharmacogenomics 11(12): 1743-1750.	Title
442	Scheen, A. J. (2005). "Drug interactions of clinical importance with antihyperglycaemic agents: an update." Drug safety 28: 601-631.	Review
443	Scheen, A. J. (2014). "[Canagliflozin (Invokana): kidney SGLT2 cotransporter inhibitor for treating type 2 diabetes]." Rev Med Liege 69(12): 692-699.	Title
444	Schroner, Z., et al. (2011). "Pharmacogenetics of oral antidiabetic treatment." Bratisl Lek Listy 112(8): 441-446.	Title
445	Schwartz, S. L. (2010). "Treatment of elderly patients with type 2 diabetes mellitus: a systematic review of the benefits and risks of dipeptidyl peptidase-4 inhibitors." Am J Geriatr Pharmacother 8(5): 405-418.	Review
446	Schwarz, U. (2003). "Clinical relevance of genetic polymorphisms in the human CYP2C9 gene." European journal of clinical investigation 33: 23-30.	Title

447	Scott, L. J. (2012). "Repaglinide: a review of its use in type 2 diabetes mellitus." Drugs 72(2): 249-272.	Review
448	Scott, L. J. (2015). "Teneligliptin: a review in type 2 diabetes." Clin Drug Investig 35(11): 765-772.	Review
449	Segregur, D., et al. (2019). "Impact of Acid-Reducing Agents on Gastrointestinal Physiology and Design of Biorelevant Dissolution Tests to Reflect These Changes." Journal of Pharmaceutical Sciences 108(11): 3461-3477.	Review
450	Sesti, G. and M. Hribal (2006). "Pharmacogenetics in type 2 diabetes: polymorphisms in candidate genes affecting responses to antidiabetic oral treatment." Current Pharmacogenomics 4(1): 69-78.	Title
451	Shabab, S., et al. (2021). "Protective effects of medicinal plant against diabetes induced cardiac disorder: A review." Journal of Ethnopharmacology 265: 113328.	Review
452	Shakeri-Nejad, K., et al. (2013). "Influence of mild and moderate liver impairment on the pharmacokinetics and metabolism of almorexant, a dual orexin receptor antagonist." European Journal of Pharmaceutical Sciences 49(5): 836-844.	Title
453	Shakweh, E. Y. (2018). Developing a Stratified Approach to Treatment in Type 2 Diabetes, University of Exeter (United Kingdom).	Title
454	Shao, H., et al. (2016). "Metabolic Interaction Potential between Clopidogrel and Sulfonylurea Antidiabetic Agents: Effects on Clopidogrel Bioactivation." Pharmacology 97(1-2): 18-24.	Title
455	Sharma, A. K., et al. (2012). "Sitagliptin, sitagliptin and metformin, or sitagliptin and amitriptyline attenuate streptozotocin-nicotinamide induced diabetic neuropathy in rats." Journal of Biomedical Research 26(3): 200-210.	Animal
456	Sharma, S. (2016). "Advantages of Teneligliptin Compared with Other DPP-4 Inhibitors in T2DM." IJCP Group of Publications 27(5): 436.	Title
457	Sharma, S., et al. (2023). "A Compendium of Bioavailability Enhancement via Niosome Technology." Pharm Nanotechnol 11(4): 324-338.	Title
458	Shashikumar, U., et al. (2023). "Trajectory in biological metal-organic frameworks: Biosensing and sustainable strategies-perspectives and challenges." International Journal of Biological Macromolecules: 127120.	Title
459	Shin, E., et al. (2017). "High-Dose Metformin May Increase the Concentration of Atorvastatin in the Liver by Inhibition of Multidrug Resistance–Associated Protein 2." Journal of Pharmaceutical Sciences 106(4): 961-967.	Title
460	Shin, K. H., et al. (2011). "Pharmacokinetic comparison of a new sustained-release formulation of glimepiride/metformin 1/500 mg combination tablet and a sustained-release formulation of glimepiride/metformin 2/500 mg combination tablet in healthy Korean male volunteers: a randomized, 2-sequence, 2-period, 2-treatment crossover study." Clin Ther 33(11): 1809-1818.	Abstract
461	Shi-Ying, J. I. N., et al. (2014). "Characterization and evaluation in vivo of baicalin-nanocrystals prepared by an ultrasonic-homogenization-fluid bed drying method." Chinese Journal of Natural Medicines 12(1): 71-80.	Title
462	Shobha, J. C. and M. R. Muppidi (2010). "Interaction between voriconazole and glimepiride." J Postgrad Med 56(1): 44-45.	Abstract
463	Shukla, U., et al. (1994). "Single dose and steadystate pharmacokinetics of glimepiride (G), a new sulfonylurea, in patients with non–insulin dependent diabetes mellitus (NIDDM)." Pharm Res 11: S368.	Review
464	Shuster, D. L., et al. (2011). "Glyburide Disposition During Pregnancy." Gestational Diabetes: 325.	Title

465	Silva Filho, E. Q. d. (2011). Estudo de propriedades físico-químicas da glibenclamida que influem sobre resultados do ensaio de dissolução para medicamento similar e genérico.	Language
466	Simos, Y. V., et al. (2021). "Trends of nanotechnology in type 2 diabetes mellitus treatment." Asian Journal of Pharmaceutical Sciences 16(1): 62-76.	Review
467	Sinz, M., et al. (2006). "Evaluation of 170 xenobiotics as transactivators of human pregnane X receptor (hPXR) and correlation to known CYP3A4 drug interactions." Curr Drug Metab 7(4): 375-388.	Title
468	Song, C., et al. (2022). "Impact of CYP2C19 and CYP2C9 gene polymorphisms on sodium valproate plasma concentration in patients with epilepsy." European Journal of Hospital Pharmacy 29(4): 198-201.	Title
469	Sonnenberg, G. E., et al. (1997). "Short-term comparison of once-versus twice-daily administration of glimepiride in patients with non-insulin-dependent diabetes mellitus." Annals of Pharmacotherapy 31(6): 671-676.	Abstract
470	Stalker, D., et al. (1994). "The effect of age and dosing regimen on the pharmacokinetics of glimepiride in subjects with noninsulin dependent diabetes mellitus." Pharm Res 11: S-339.	Title
471	Stevens, P. E., et al. (2013). "Evaluation and management of chronic kidney disease: synopsis of the kidney disease: improving global outcomes 2012 clinical practice guideline." Annals of internal medicine 158(11): 825-830.	Title
472	Stingl, J. (2004). Arzneitherapieempfehlungen auf pharmakogenetischer Basis, Berlin, Humboldt-Univ., Habil.-Schr., 2004.	Language
473	Strugaru, A.-M., et al. (2019). "Simultaneous determination of metformin and glimepiride in human serum by ultra high performance liquid chromatography quadrupole time of flight mass spectrometry detection." Journal of Pharmaceutical and Biomedical Analysis 165: 276-283.	Abstract
474	Suarez-Kurtz, G. (2014). "Impact of CYP2C9* 3/* 3 genotype on the pharmacokinetics and pharmacodynamics of oxicam NSAIDs." Pharmacogenetics and Genomics 24(8): 406-407.	Title
475	Subba, R., et al. (2022). "Targeting NRF2 in Type 2 diabetes mellitus and depression: Efficacy of natural and synthetic compounds." European Journal of Pharmacology 925: 174993.	Review
476	Subramanian, M., et al. (2012). "Effect of P450 oxidoreductase variants on the metabolism of model substrates mediated by CYP2C9. 1, CYP2C9. 2, and CYP2C9. 3." Pharmacogenetics and genomics 22(8): 590-597.	Title
477	Sun, L., et al. (2018). "The Solubility-Permeability Trade-Off of Progesterone With Cyclodextrins Under Physiological Conditions: Experimental Observations and Computer Simulations." Journal of Pharmaceutical Sciences 107(1): 488-494.	Title
478	Surendiran, A., et al. (2011). "Influence of CYP2C9 gene polymorphisms on response to glibenclamide in type 2 diabetes mellitus patients." European journal of clinical pharmacology 67: 797-801.	Title
479	Surendran, S., et al. (2017). "A validated LC–MS/MS method for the estimation of glimepiride and pitavastatin in rat plasma: Application to drug interaction studies." Journal of Chromatography B 1046: 218-225.	Animal
480	Suriyo, T., et al. (2021). "Interactive effects of Andrographis paniculata extracts and cancer chemotherapeutic 5-Fluorouracil on cytochrome P450s expression in human hepatocellular carcinoma HepG2 cells." Journal of Herbal Medicine 26: 100421.	Title

481	Suthar, S. K., et al. (2022). "Quinoxaline: A comprehension of current pharmacological advancement in medicinal chemistry." European Journal of Medicinal Chemistry Reports 5: 100040.	Review
482	Swen, J. J., et al. (2010). "Effect of CYP2C9 polymorphisms on prescribed dose and time-to-stable dose of sulfonylureas in primary care patients with Type 2 diabetes mellitus." Pharmacogenomics 11(11): 1517-1523.	Title
483	Szmitko, P. E., et al. (2010). "The incretin system and cardiometabolic disease." Canadian Journal of Cardiology 26(2): 87-95.	Review
484	Tahboub, Y. R. (2014). "Chromatographic behavior of co-eluted plasma compounds and effect on screening of drugs by APCI-LC–MS(/MS): Applications to selected cardiovascular drugs." Journal of Pharmaceutical Analysis 4(6): 384-391.	Title
485	TANG, C., et al. (2003). "A Simple and Rapid RP-HPLC Assay Method to Determine Glimepiride Concentration in Human Plasma." Chinese Journal of Pharmaceutical Analysis 23(4): 271-273.	Language
486	Tateishi, H., et al. (2021). "Hypoglycemia possibly caused by CYP2C9-mediated drug interaction in combination with bucolome: a case report." Journal of Pharmaceutical Health Care and Sciences 7: 1-6.	Title
487	Teague, S. and K. Valko (2017). "How to identify and eliminate compounds with a risk of high clinical dose during the early phase of lead optimisation in drug discovery." European Journal of Pharmaceutical Sciences 110: 37-50.	Abstract
488	Thakkar, D. and R. P. Dash (2017). "Pharmacokinetic interactions between glimepiride and rosuvastatin in healthy Korean subjects: does the SLCO1B1 or CYP2C9 genetic polymorphism affect these drug interactions? Observations and introspection of the bioanalysis." Drug Design, Development and Therapy: 1263-1265.	Abstract
489	Thikekar, A. K., et al. (2022). "Effect of herbal formulation on glimepiride pharmacokinetics and pharmacodynamics in nicotinamide-streptozotocin-induced diabetic rats." J Ayurveda Integr Med 13(3): 100633.	Animal
490	Thompson, A. M. and J. M. Trujillo (2015). "Dulaglutide: the newest GLP-1 receptor agonist for the management of type 2 diabetes." Ann Pharmacother 49(3): 351-359.	Review
491	Thrasher, J. (2017). "Pharmacologic Management of Type 2 Diabetes Mellitus: Available Therapies." The American Journal of Cardiology 120(1, Supplement): S4-S16.	Title
492	Timm, R., et al. (2005). "Association of cyclophosphamide pharmacokinetics to polymorphic cytochrome P450 2C19." The pharmacogenomics journal 5(6): 365-373.	Title
493	Tkáč, I. (2015). "Genetics of drug response in type 2 diabetes." Current Diabetes Reports 15: 1-9.	Title
494	Tomlinson, B., et al. (2017). "Evaluation of the pharmacokinetics, pharmacodynamics and clinical efficacy of empagliflozin for the treatment of type 2 diabetes." Expert Opinion on Drug Metabolism & Toxicology 13(2): 211-223.	Review
495	Tomlinson, B., et al. (2022). "Evaluating gliclazide for the treatment of type 2 diabetes mellitus." Expert Opin Pharmacother 23(17): 1869-1877.	Review
496	Tornio, A., et al. (2012). "Drug interactions with oral antidiabetic agents: pharmacokinetic mechanisms and clinical implications." Trends in pharmacological sciences 33(6): 312-322.	Review
497	Trujillo, J. M. and W. Nuffer (2014). "Albiglutide: a new GLP-1 receptor agonist for the treatment of type 2 diabetes." Ann Pharmacother 48(11): 1494-1501.	Review

498	Tsai, M., et al. (2013). "Pharmacokinetics (PK) & pharmacodynamics (PD) of the GPR40 agonist TAK-875 and glimepiride following co-administration in subjects with type II diabetes (T2DM)." Diabetes 62: A303.	Title
499	Tsunekawa, T., et al. (2003). "Plasma adiponectin plays an important role in improving insulin resistance with glimepiride in elderly type 2 diabetic subjects." Diabetes care 26(2): 285-289.	Abstract
500	Tye, S. C., et al. "Biomarkers and Precision Medicine in Diabetes."	Review
501	Uçaktürk, E. (2013). "Development of a gas chromatography–mass spectrometry method for the analysis of sitagliptin in human urine." Journal of Pharmaceutical and Biomedical Analysis 74: 71-76.	Title
502	Ueno, K., et al. (2002). "Evaluation of mexiletine clearance in a Japanese population." Annals of Pharmacotherapy 36(2): 241-245.	Title
503	Unger, J. (2008). "Current Strategies for Evaluating, Monitoring, and Treating Type 2 Diabetes Mellitus." The American Journal of Medicine 121(6, Supplement): S3-S8.	Title
504	Unger, M. S., et al. (2019). "Clinically relevant OATP2B1 inhibitors in marketed drug space." Molecular pharmaceutics 17(2): 488-498.	Title
505	Uwaifo, G. I. and R. E. Ratner (2007). "Differential Effects of Oral Hypoglycemic Agents on Glucose Control and Cardiovascular Risk." The American Journal of Cardiology 99(4, Supplement): 51-67.	Review
506	Vaidyanathan, J., et al. (2012). "Type 2 diabetes in pediatrics and adults: thoughts from a clinical pharmacology perspective." Journal of pharmaceutical sciences 101(5): 1659-1671.	Review
507	Valko, K., et al. (2012). "In Vitro Measurement of Drug Efficiency Index to Aid Early Lead Optimization." Journal of Pharmaceutical Sciences 101(11): 4155-4169.	Title
508	Van Booven, D., et al. (2010). "Cytochrome P450 2C9-CYP2C9." Pharmacogenetics and genomics 20(4): 277.	Review
509	van de Steeg, E., et al. (2013). "Drug-drug interactions between rosuvastatin and oral antidiabetic drugs occurring at the level of OATP1B1." Drug Metab Dispos 41(3): 592-601.	Abstract
510	Van Leeuwen, N., et al. (2013). "The role of pharmacogenetics in drug disposition and response of oral glucose-lowering drugs." Clinical pharmacokinetics 52: 833-854.	Review
511	Varma, M. V., et al. (2014). "Mechanism-based pharmacokinetic modeling to evaluate transporter-enzyme interplay in drug interactions and pharmacogenetics of glyburide." The AAPS journal 16: 736-748.	Title
512	Vendramini, M. F. (2007). "[Glimepiride and the bioequivalence tests]." Arq Bras Endocrinol Metabol 51(6): 898-899.	Title
513	Veneman, T. F., et al. (1998). "The newly developed sulfonylurea glimepiride: a new ingredient, an old recipe." Neth J Med 52(5): 179-186.	Review
514	Verma, D. and S. K. Sharma (2021). "Recent advances in guar gum based drug delivery systems and their administrative routes." International Journal of Biological Macromolecules 181: 653-671.	Title
515	Viana, A. L. M., et al. (2018). "Pharmacokinetics and pharmacodynamics of glimepiride polymorphs." International Journal of Pharmaceutics 553(1-2): 272-280.	Animal

516	Viswanathan, P., et al. (2013). "Pharmacokinetics (PK) and pharmacodynamics (PD) of the GPR40 agonist fasiglifam (TAK-875) and glimepiride following co-administration in type 2 diabetes subjects." Diabetologia 56: S353.	Abstract
517	Vivian, E. M. (2015). "Dapagliflozin: a new sodium-glucose cotransporter 2 inhibitor for treatment of type 2 diabetes." Am J Health Syst Pharm 72(5): 361-372.	Review
518	Vondracek, S. F., et al. (2021). "Principles of Kidney Pharmacotherapy for the Nephrologist: Core Curriculum 2021." American Journal of Kidney Diseases 78(3): 442-458.	Review
519	Voronkov, M., et al. (2022). "Modifying naloxone to reverse fentanyl-induced overdose." International Journal of Pharmaceutics 611: 121326.	Title
520	Vučićević, K. and B. Miljković "Gojaznost kao faktor farmakokinetičke varijabilnosti."	Language
521	Vučićević, K. and B. Miljković (2011). "Obesity as a factor of pharmacokinetic variability." Arhiv za farmaciju 61(4): 365-382.	Review
522	Vullendula, S. K. A., et al. (2022). "Polymeric solid dispersion Vs co-amorphous technology: A critical comparison." Journal of Drug Delivery Science and Technology 78: 103980.	Title
523	Vyas, M. and V. J. Galani (2010). "In vivo and In vitro Drug Interactions Study of Glimepride with Atorvastatin and Rosuvastatin." Journal of Young Pharmacists 2(2): 196-200.	Animal
524	Wang, B., et al. (2023). "Discovery of a structurally novel, potent, and once-weekly free fatty acid receptor 1 agonist for the treatment of diabetes." European Journal of Medicinal Chemistry 245: 114883.	Title
525	Wang, D., et al. (2022). "Clinical significance of the series of CYP2C9* non3 variants, an unignorable predictor of warfarin sensitivity in Chinese population." Frontiers in Cardiovascular Medicine 9: 1052521.	Title
526	Wang, R., et al. (2005). "Pharmacokinetics of glimepiride and cytochrome P450 2C9 genetic polymorphisms." Clinical Pharmacology & Therapeutics 78(1): 90-92.	Full-Length Text

527	Watson, W. A., et al. (2004). "2003 annual report of the American Association of Poison Control Centers Toxic Exposure Surveillance System1 1US poison centers make possible the compilation and reporting of this comprehensive description of human exposures to potentially toxic substances through their meticulous documentation of each case using standardized definitions and compatible computer systems. Participating centers include: Regional Poison Control Center, Birmingham, AL; Alabama Poison Center, Tuscaloosa, AL; Arizona Poison and Drug Information Center, Tucson, AZ; Banner Poison Control Center, Phoenix, AZ; Arkansas Poison and Drug Information Center, Little Rock, AR; California Poison Control System—Fresno/Madera Division, CA; California Poison Control System—Sacramento Division, CA; California Poison Control System—San Diego Division, CA; California Poison Control System—San Francisco Division, CA; Rocky Mountain Poison and Drug Center, Denver, CO; Connecticut Poison Control Center, Farmington, CT; National Capital Poison Center, Washington, DC; Florida Poison Information Center, Tampa, FL; Florida Poison Information Center, Jacksonville, FL; Florida Poison Information Center, Miami, FL; Georgia Poison Center, Atlanta, GA; Illinois Poison Center, Chicago, IL; Indiana Poison Center, Indianapolis, IN; Iowa Statewide Poison Control Center, Sioux City, IA; Mid-America Poison Control Center, Kansas City, KS; Kentucky Regional Poison Center, Louisville, KY; Louisiana Drug and Poison Information Center, Monroe, LA; Northern New England Poison Center, Portland, ME; Maryland Poison Center, Baltimore, MD; Regional Center for Poison Control and Prevention Serving Massachusetts and Rhode Island, Boston, MA; Children's Hospital of Michigan Regional Poison Control Center, Detroit, MI; DeVos Children's Hospital Regional Poison Center, Grand Rapids, MI; Hennepin Regional Poison Center, Minneapolis, MN; Mississippi Regional Poison Control Center, Jackson, MS; Missouri Regional Poison Center, St. Louis, MO; Nebraska Regional Poison Center, Omaha, NE; New Hampshire Poison Information Center, Lebanon, NH; New Jersey Poison Information and Education System, Newark, NJ; New Mexico Poison and Drug Information Center, Albuquerque, NM; New York City Poison Control Center, New York, NY; Long Island Regional Poison and Drug Information Center, Mineola, NY; Finger Lakes Regional Poison and Drug Information Center, Rochester, NY; Central New York Poison Center, Syracuse, NY; Western New York Poison Center, Buffalo, NY; Carolinas Poison Center, Charlotte, NC; Cincinnati Drug and Poison Information Center, Cincinnati, OH; Central Ohio Poison Center, Columbus, OH; Greater Cleveland Poison Control Center, Cleveland, OH; Oklahoma Poison Control Center, Oklahoma City, OK; Oregon Poison Center, Portland, OR; Pittsburgh Poison Center, Pittsburgh, PA; The Poison Control Center, Philadelphia, PA; Penn State Poison Center, Hershey, PA; San Jorge Children's Hospital Poison Center, Santurce, PR; Palmetto Poison Center, Columbia, SC; Tennessee Poison Center, Nashville, TN; Southern Poison Center, Memphis, TN; Central Texas Poison Center, Temple, TX; North Texas Poison Center, Dallas, TX; Southeast Texas Poison Center, Galveston, TX; Texas Panhandle Poison Center, Amarillo, TX; West Texas Regional Poison Center, El Paso, TX; South Texas Poison Center, San Antonio, TX; Utah Poison Control Center, Salt Lake City, UT; Virginia Poison Center, Richmond, VA; Blue Ridge Poison Center, Charlottesville, VA; Washington Poison Center, Seattle, WA; West Virginia Poison Center, Charleston, WV; and Children's Hospital of Wisconsin Poison Center, Milwaukee, WI." The American Journal of Emergency Medicine 22(5): 335-404.	Title
528	Watson, W. A., et al. (2005). "2004 Annual report of the American Association of Poison Control Centers Toxic Exposure Surveillance System." The American Journal of Emergency Medicine 23(5): 589-666.	Title
529	White, J. (2009). "Efficacy and safety of incretin-based therapies: Clinical trial data." Journal of the American Pharmacists Association 49(5, Supplement 1): S30-S40.	Review
530	Williams, M. E. and R. Garg (2014). "Glycemic Management in ESRD and Earlier Stages of CKD." American Journal of Kidney Diseases 63(2, Supplement 2): S22-S38.	Title
531	Wu, J., et al. (2009). "Dipeptidyl peptidase IV(DPP IV): a novel emerging target for the treatment of type 2 diabetes." Journal of Nanjing Medical University 23(4): 228-235.	Review

532	Wu, J.-j., et al. (2024). "Ginsenoside Rg1, lights up the way for the potential prevention of Alzheimer's disease due to its therapeutic effects on the drug-controllable risk factors of Alzheimer's disease." Journal of Ethnopharmacology 318: 116955.	Not accessible
533	Xu, H., et al. (2009). "Influence of genetic polymorphisms on the pharmacokinetics and pharmacodynamics of sulfonylurea drugs." Current drug metabolism 10(6): 643-658.	Not accessible
534	Yang, F., et al. (2018). "OATP1B3 (699G> A) and CYP2C9* 2,* 3 significantly influenced the transport and metabolism of glibenclamide and glipizide." Scientific Reports 8(1): 18063.	Abstract
535	Yang, F., et al. (2018). "CYP2C9 and OATP1B1 genetic polymorphisms affect the metabolism and transport of glimepiride and gliclazide." Sci Rep 8(1): 10994.	Abstract
536	Yang, K., et al. (2013). "An updated review on drug-induced cholestasis: Mechanisms and investigation of physicochemical properties and pharmacokinetic parameters." Journal of Pharmaceutical Sciences 102(9): 3037-3057.	Review
537	Yang, P., et al. (2016). "Pharmacogenetics and personalized treatment of type 2 diabetes mellitus." International Journal of Diabetes in Developing Countries 36: 508-518.	Review
538	Yang, W., et al. (2018). "Determination of tranilast in bio-samples by LC–MS/MS: Application to a pharmacokinetic and brain tissue distribution study in rats." Journal of Pharmaceutical and Biomedical Analysis 147: 479-484.	Animal
539	Yee, J., et al. (2021). "Association between the CYP2C9 genotype and hypoglycemia among patients with type 2 diabetes receiving sulfonylurea treatment: a meta-analysis." Clinical Therapeutics 43(5): 836-843. e834.	Abstract
540	Yen, F.-S., et al. (2020). "Sulfonylureas may be useful for glycemic management in patients with diabetes and liver cirrhosis." Plos one 15(12): e0243783.	Review
541	Yoo, H. D., et al. (2011). "Population pharmacokinetic analysis of glimepiride with CYP2C9 genetic polymorphism in healthy Korean subjects." Eur J Clin Pharmacol 67(9): 889-898.	Title
542	Yu, X., et al. (2020). "Development and Characterization of a Glimepiride-Loaded Gelatin-Coated Mesoporous Hollow Silica Nanoparticle Formulation and Evaluation of Its Hypoglycemic Effect on Type-2 Diabetes Model Rats." Assay Drug Dev Technol 18(8): 369-378.	Animal
543	Yun, H. Y., et al. (2006). "Pharmacokinetic and pharmacodynamic modelling of the effects of glimepiride on insulin secretion and glucose lowering in healthy humans." Journal of clinical pharmacy and therapeutics 31(5): 469-476.	Abstract
544	Zafar, M. I. (2020). "Suitability of APINCH high-risk medications use in diabetes mellitus." European Journal of Pharmacology 867: 172845.	Review
545	Zakrzewski-Jakubiak, M., et al. (2010). "Enantioselective quantification of carvedilol in human plasma by HPLC in heavily medicated heart failure patients." Journal of Pharmaceutical and Biomedical Analysis 52(4): 636-641.	Title
546	Zargar, A., et al. (2005). "Sulphonylureas in the management of type 2 diabetes during the fasting month of Ramadan." J Indian Med Assoc 103(8): 444-446.	Review
547	Zhang, C., et al. (2010). "Validated LC–MS/MS method for the determination of sarpogrelate in human plasma: Application to a pharmacokinetic and bioequivalence study in Chinese volunteers." Journal of Pharmaceutical and Biomedical Analysis 53(3): 546-551.	Title

548	Zhang, Q., et al. (2023). "Identification and in vitro functional assessment of 10 CYP2C9 variants found in Chinese Han subjects." Frontiers in Endocrinology 14: 1139805.	Title
549	Zhang, T., et al. (2022). "Drug pharmacokinetics in the obese population: challenging common assumptions on predictors of obesity-related parameter changes." Expert Opinion on Drug Metabolism & Toxicology 18(10): 657-674.	Review
550	Zhang, W., et al. (2015). "Nanomicelles based on X-shaped four-armed peglyated distearylglycerol as long circulating system for doxorubicin delivery." European Journal of Pharmaceutical Sciences 66: 96-106.	Title
551	ZHANG, W., et al. (2007). "Progress and research in pharmacogenetics of oral antidiabetic drugs." Chinese Journal of Clinical Pharmacology and Therapeutics 12(1): 7.	Title
552	Zhang, X., et al. (2020). "Development of small molecule inhibitors targeting NLRP3 inflammasome pathway for inflammatory diseases." European Journal of Medicinal Chemistry 185: 111822.	Review
553	Zhang, Y., et al. (2015). "Different effects of two dipeptidyl peptidase-4 inhibitors and glimepiride on beta-cell function in a newly designed two-step hyperglycemic clamp." Journal of diabetes 7(2): 213-221.	Not accessible
554	Zhao, L., et al. (2023). "Network pharmacology, a promising approach to reveal the pharmacology mechanism of Chinese medicine formula." Journal of Ethnopharmacology 309: 116306.	Title
555	Zharikova, O. L., et al. (2009). "Identification of the major human hepatic and placental enzymes responsible for the biotransformation of glyburide." Biochemical pharmacology 78(12): 1483-1490.	Title
556	Zhivkova, Z. and I. Doytchinova (2012). "Quantitative structure—plasma protein binding relationships of acidic drugs." Journal of Pharmaceutical Sciences 101(12): 4627-4641.	Title
557	Zhivkova, Z. and I. Doytchinova (2013). "Quantitative structure–clearance relationships of acidic drugs." Molecular pharmaceutics 10(10): 3758-3768.	Title
558	Zhou, K., et al. (2016). "Pharmacogenomics in diabetes mellitus: insights into drug action and drug discovery." Nature Reviews Endocrinology 12(6): 337-346.	Review
559	Zhou, S., et al. (2017). "A rapid hydrophilic interaction liquid chromatographic determination of glimepiride in pharmaceutical formulations." Saudi pharmaceutical journal 25(6): 852-856.	Abstract
560	Zhou, Y., et al. (2014). "Development and Validation of Liquid Chromatographic Tandem Mass Spectrometry for Determination and Pharmacokinetic Study of Glimepiride in Rat Plasma." Lat. Am. J. Pharm 33(4): 645-650.	Animal
561	Zhu, J., et al. (2020). "Magnetic solid phase extraction followed with LC-MS/MS for determination of glimepiride in beagle dog plasma and its application to bioequivalence study." Journal of Pharmaceutical and Biomedical Analysis 184: 113180.	Animal
562	Zhukova, L., et al. (2015). "The role of glimepiride in combination with metformin in modern practice of using antihyperglycemic therapy for type 2 diabetes mellitus." Consilium Medicum 17(4): 20-25.	Language
563	Городецкая, Г., et al. (2017). "Фармакогенетическое тестирование в оптимизации терапии сахарного диабета 2 типа препаратами сульфонилмочевины." Ведомости научного центра экспертизы средств медицинского применения 7(4): 233-241.	Language

564	Жукова, Л. А., et al. (2015). "Роль глимепирида в комбинации с метформином в современной практике сахароснижающей терапии при сахарном диабете типа 2." Consilium medicum 17(4): 20-25.	Language
565	Киселева, Т., et al. (2014). "Биохимические и генетические аспекты персонализации диагностики и лечения сахарного диабета." Вестник Казанского технологического университета 17(24): 136-140.	Language
566	Кононенко, И. В., et al. (2015). "Фармакогенетика сахароснижающих препаратов." Сахарный диабет 18(4): 28-34.	Language
567	Поздняков, Н., et al. (2020). "Фармакогенетические аспекты в терапии сахарного диабета 2-го типа." Acta Biomedica Scientifica 5(3): 13-23.	Language
568	Полторак, В., et al. "Международный эндокринологический журнал 6 (54) 2013."	Language
569	Полторак, В. and В. Липсон (2013). "Бренды и генерики: критерии оценки эффективности." Международный эндокринологический журнал(6 (54)): 61-70.	Language
570	Руяткина, Л. А. and М. Ю. Сорокин (2012). "Глимепирид в современной гипогликемизирующей терапии: безопасность и эффективность." Сахарный диабет(2): 89-97.	Language
571	Царукян, А. А. (2015). Этнические особенности применения варфарина у жителей Ставропольского края: клинические и фармакогенетические аспекты.	Language
572	เสถียร, ส. ร. ย. จ. ม. ห. (2006). "เภสัช จลนพลศาสตร์ ของ ยา ใน ผู้ ป่วย อ้วน เกิน." Thai Bulletin of Pharmaceutical Sciences 3(1): 53-65.	Language
573	김동욱 (2006). "Frequency of Cytochrome P450 2C9 Mutant Alleles in a Korean Population and Pharmacokinetics of Glimepiride according to the CYP2C9 Genotype." 대한임상약리학회 학술대회: 45-46.	Language
574	刘婧 and 熊玉卿 (2012). "基因多态性对氟伐他汀药代动力学和药效学特征的影响." 中国临床药理学与治疗学 17(11): 1282.	Language
575	大山貴子, et al. (2012). "血中ミチグリニド濃度高値を呈した遷延性重症低血糖の 1 例." 糖尿病 55(3): 199-203.	Language
576	张伟, et al. (2007). "口服降糖药的遗传药理学研究进展." 中国临床药理学与治疗学 12(1): 7.	Language
577	张逸凡, et al. (2005). 细胞色素 P450 CYP2C9* 3 对格列本脲和氯诺昔康中国人体药代动力学的影响.	Language
578	李赟, et al. (2012). "CYP2C9, CYP2C19, CYP3A4 基因多态性对磺脲类降糖药代谢的影响." 中国临床药理学与治疗学 17(5): 582.	Language
579	淡海丽 and 易飞 (2011). "吉非罗齐对临床几种常用口服降糖药的作用." 中国临床药理学与治疗学 16(1): 116.	Language
580	田邊智子, et al. (2005). "遺伝子多型情報に基づく投与指針作成に向けて CYP2C9." 臨床薬理 36(5): 255-260.	Language
581	黄作君, et al. (2007). "CYP2C9 基因多态性与磺酰脲类降糖药药代动力学药效学相关性的研究进展." 中国临床药理学与治疗学 12(2): 219.	Language

PRISMA 2020 CHECKLIST

Section and Topic	Item #	Checklist item	Location where item is reported
**TITLE**			
Title	1	Identify the report as a systematic review.	1
**ABSTRACT**			
Abstract	2	See the PRISMA 2020 for Abstracts checklist.	2
**INTRODUCTION**			
Rationale	3	Describe the rationale for the review in the context of existing knowledge.	4
Objectives	4	Provide an explicit statement of the objective(s) or question(s) the review addresses.	4
**METHODS**			
Eligibility criteria	5	Specify the inclusion and exclusion criteria for the review and how studies were grouped for the syntheses.	6
Information sources	6	Specify all databases, registers, websites, organisations, reference lists, and other sources searched or consulted to identify studies. Specify the date when each source was last searched or consulted.	5
Search strategy	7	Present the full search strategies for all databases, registers and websites, including any filters and limits used.	5
Selection process	8	Specify the methods used to decide whether a study met the inclusion criteria of the review, including how many reviewers screened each record and each report retrieved, whether they worked independently, and if applicable, details of automation tools used in the process.	6
Data collection process	9	Specify the methods used to collect data from reports, including how many reviewers collected data from each report, whether they worked independently, any processes for obtaining or confirming data from study investigators, and	6

Section and Topic	Item #	Checklist item	Location where item is reported
		if applicable, details of automation tools used in the process.	
Data items	10a	List and define all outcomes for which data were sought. Specify whether all results that were compatible with each outcome domain in each study were sought (e.g. for all measures, time points, analyses), and if not, the methods used to decide which results to collect.	6
	10b	List and define all other variables for which data were sought (e.g. participant and intervention characteristics, funding sources). Describe any assumptions made about any missing or unclear information.	6
Study risk of bias assessment	11	Specify the methods used to assess risk of bias in the included studies, including details of the tool(s) used, how many reviewers assessed each study and whether they worked independently, and if applicable, details of automation tools used in the process.	7
Effect measures	12	Specify for each outcome the effect measure(s) (e.g. risk ratio, mean difference) used in the synthesis or presentation of results.	5, 6
Synthesis methods	13a	Describe the processes used to decide which studies were eligible for each synthesis (e.g. tabulating the study intervention characteristics and comparing against the planned groups for each synthesis (item #5)).	5, 6
	13b	Describe any methods required to prepare the data for presentation or synthesis, such as handling of missing summary statistics, or data conversions.	5, 6
	13c	Describe any methods used to tabulate or visually display results of individual studies and syntheses.	5, 6
	13d	Describe any methods used to synthesize results and provide a rationale for the choice(s). If meta-analysis was performed, describe the model(s), method(s) to identify the presence and extent of statistical heterogeneity, and software package(s) used.	6, 7
	13e	Describe any methods used to explore possible causes of	6, 7

Section and Topic	Item #	Checklist item	Location where item is reported
		heterogeneity among study results (e.g. subgroup analysis, meta-regression).	
	13f	Describe any sensitivity analyses conducted to assess robustness of the synthesized results.	6, 7
Reporting bias assessment	14	Describe any methods used to assess risk of bias due to missing results in a synthesis (arising from reporting biases).	7
Certainty assessment	15	Describe any methods used to assess certainty (or confidence) in the body of evidence for an outcome.	6, 7
**RESULTS**			
Study selection	16a	Describe the results of the search and selection process, from the number of records identified in the search to the number of studies included in the review, ideally using a flow diagram.	7
	16b	Cite studies that might appear to meet the inclusion criteria, but which were excluded, and explain why they were excluded.	6
Study characteristics	17	Cite each included study and present its characteristics.	9-13
Risk of bias in studies	18	Present assessments of risk of bias for each included study.	7, 8
Results of individual studies	19	For all outcomes, present, for each study: (a) summary statistics for each group (where appropriate) and (b) an effect estimate and its precision (e.g. confidence/credible interval), ideally using structured tables or plots.	14, 19, 21, 25 and 29
Results of syntheses	20a	For each synthesis, briefly summarise the characteristics and risk of bias among contributing studies.	6, 7 Supp File page no 42
	20b	Present results of all statistical syntheses conducted. If meta-analysis was done, present for each the summary estimate and its precision (e.g. confidence/credible interval) and measures of statistical heterogeneity. If comparing groups, describe the direction of the effect.	33

Section and Topic	Item #	Checklist item	Location where item is reported
	20c	Present results of all investigations of possible causes of heterogeneity among study results.	33-37
	20d	Present results of all sensitivity analyses conducted to assess the robustness of the synthesized results.	33-37
Reporting biases	21	Present assessments of risk of bias due to missing results (arising from reporting biases) for each synthesis assessed.	Supp File page no 42
Certainty of evidence	22	Present assessments of certainty (or confidence) in the body of evidence for each outcome assessed.	33-37
**DISCUSSION**			
Discussion	23a	Provide a general interpretation of the results in the context of other evidence.	37, 38
	23b	Discuss any limitations of the evidence included in the review.	38. 39
	23c	Discuss any limitations of the review processes used.	38, 39
	23d	Discuss implications of the results for practice, policy, and future research.	39
**OTHER INFORMATION**			
Registration and protocol	24a	Provide registration information for the review, including register name and registration number, or state that the review was not registered.	N/A
	24b	Indicate where the review protocol can be accessed, or state that a protocol was not prepared.	N/A
	24c	Describe and explain any amendments to information provided at registration or in the protocol.	N/A
Support	25	Describe sources of financial or non-financial support for the review, and the role of the funders or sponsors in the review.	39
Competing interests	26	Declare any competing interests of review authors.	39
Availability of data, code and other materials	27	Report which of the following are publicly available and where they can be found: template data collection forms; data extracted from included studies; data used for all	39

Section and Topic	Item #	Checklist item	Location where item is reported
		analyses; analytic code; any other materials used in the review.	

From: Page MJ, McKenzie JE, Bossuyt PM, Boutron I, Hoffmann TC, Mulrow CD, et al. The PRISMA 2020 statement: an updated guideline for reporting systematic reviews. BMJ 2021;372:n71. doi: 10.1136/bmj.n71

References

1. Yoo, H.; Kim, Y.; Jang, I.-J.; Yu, K.-S.; Lee, S. Pharmacokinetic/pharmacodynamic interactions between evogliptin and glimepiride in healthy male subjects. *Drug Design, Development and Therapy* **2020**, 5179-5187.
2. Dawra, V.K.; Cutler, D.L.; Zhou, S.; Krishna, R.; Shi, H.; Liang, Y.; Alvey, C.; Hickman, A.; Saur, D.; Terra, S.G. Assessment of the drug interaction potential of ertugliflozin with sitagliptin, metformin, glimepiride, or simvastatin in healthy subjects. *Clinical pharmacology in drug development* **2019**, *8*, 314-325.
3. Ahmed, T.A.; Suhail, M.A.; Hosny, K.M.; Abd-Allah, F.I. Clinical pharmacokinetic study for the effect of glimepiride matrix tablets developed by quality by design concept. *Drug Development and Industrial Pharmacy* **2018**, *44*, 66-81.
4. Kim, C.O.; Oh, E.S.; Kim, H.; Park, M.S. Pharmacokinetic interactions between glimepiride and rosuvastatin in healthy Korean subjects: does the SLCO1B1 or CYP2C9 genetic polymorphism affect these drug interactions? *Drug Design, Development and Therapy* **2017**, 503-512.
5. Ahmed, T.A.; Khalid, M.; Aljaeid, B.M.; Fahmy, U.A.; Abd-Allah, F.I. Transdermal glimepiride delivery system based on optimized ethosomal nano-vesicles: Preparation, characterization, in vitro, ex vivo and clinical evaluation. *International journal of pharmaceutics* **2016**, *500*, 245-254.
6. Zalat, Z.A. Study of Pharmacokinetic Drug-Drug Interaction between Glimepiride and Gemfibrozil in Healthy Subjects.
7. Qiu, X.; Zhao, J.; Wang, Z.; Xu, Z.; Xu, R.-a. Simultaneous determination of bosentan and glimepiride in human plasma by ultra performance liquid chromatography tandem mass spectrometry and its application to a pharmacokinetic study. *Journal of Pharmaceutical and Biomedical Analysis* **2014**, *95*, 207-212.
8. Ni, X.-J.; Wang, Z.-Z.; Shang, D.-W.; Zhang, M.; Hu, J.-Q.; Qiu, C.; Wen, Y.-G. Simultaneous determination of glimepiride and pioglitazone in human plasma by liquid chromatography–tandem mass spectrometry and its application to pharmacokinetic study. *Journal of Chromatography B* **2014**, *960*, 247-252.
9. Jung SangHoon, J.S.; Chae JungWoo, C.J.; Song ByungJeong, S.B.; Kwon KwangIl, K.K. Bioequivalence comparison of two formulations of fixed-dose combination glimepiride/metformin (2/500 mg) tablets in healthy volunteers. **2014**.
10. He, L.; Wickremasingha, P.; Lee, J.; Tao, B.; Mendell-Harary, J.; Walker, J.; Wight, D. The effects of colesevelam HCl on the single-dose pharmacokinetics of glimepiride, extended-release glipizide, and olmesartan medoxomil. *The Journal of Clinical Pharmacology* **2014**, *54*, 61-69.
11. Choi, H.Y.; Kim, Y.H.; Kim, M.J.; Lee, S.H.; Bang, K.; Han, S.; Lim, H.-S.; Bae, K.-S. Evaluation of pharmacokinetic drug interactions between gemigliptin (dipeptidylpeptidase-4 inhibitor) and glimepiride (sulfonylurea) in healthy volunteers. *Drugs in R&D* **2014**, *14*, 165-176.
12. Polagani, S.R.; Pilli, N.R.; Gajula, R.; Gandu, V. Simultaneous determination of atorvastatin, metformin and glimepiride in human plasma by LC–MS/MS and its application to a human pharmacokinetic study. *Journal of Pharmaceutical Analysis* **2013**, *3*, 9-19.

13. Helmy, S.A.; El Bedaiwy, H.M.; Mansour, N.O. Dose linearity of glimepiride in healthy human Egyptian volunteers. *Clinical Pharmacology in Drug Development* **2013**, *2*, 264-269.
14. Smulders, R.; Zhang, W.; Veltkamp, S.; van Dijk, J.; Krauwinkel, W.; Keirns, J.; Kadokura, T. No pharmacokinetic interaction between ipragliflozin and sitagliptin, pioglitazone, or glimepiride in healthy subjects. *Diabetes, Obesity and Metabolism* **2012**, *14*, 937-943.
15. Lee, H.; Lim, M.s.; Lee, J.; Jegal, M.Y.; Kim, D.W.; Lee, W.K.; Jang, I.J.; Shin, J.G.; Yoon, Y.R. Frequency of CYP2C9 variant alleles, including CYP2C9* 13 in a Korean population and effect on glimepiride pharmacokinetics. *Journal of clinical pharmacy and therapeutics* **2012**, *37*, 105-111.
16. Dutta, L.; Ahmad, S.I.; Mishra, S.; Khuroo, A.; Monif, T. Selective, sensitive, and rapid liquid chromatography–tandem mass spectrometry method for determination of Glimepiride in human plasma. *Clinical Research and Regulatory Affairs* **2012**, *29*, 15-22.
17. Cho, H.-J.; Lee, S.-Y.; Kim, Y.-G.; Oh, S.-Y.; Kim, J.-W.; Huh, W.-S.; Ko, J.-W.; Kim, H.-G. Effect of genetic polymorphisms on the pharmacokinetics and efficacy of glimepiride in a Korean population. *Clinica Chimica Acta* **2011**, *412*, 1831-1834.
18. Noh, K.; Kim, E.; Jeong, T.; Na, M.; Baek, M.-C.; Liu, K.-H.; Park, P.-H.; Shin, B.S.; Kang, W. Simultaneous determination of glimepiride and its metabolites in human plasma by liquid chromatography coupled to a tandem mass spectrometry. *Archives of pharmacal research* **2011**, *34*, 2073-2078.
19. Kotagiri, H.; Gannu, R.; Palem, C.R.; Yamsani, S.K.; Yamsani, V.V.; Yamsani, M.R. Simultaneous determination of glimepiride and atorvastatin in human serum by high-performance liquid chromatography: Application to pharmacokinetic study. *Journal of liquid chromatography & related technologies* **2011**, *34*, 2420-2432.
20. Liu, Y.; Zhang, M.-q.; Zhu, J.-m.; Jia, J.-y.; Liu, Y.-m.; Liu, G.-y.; Li, S.; Weng, L.-p.; Yu, C. Bioequivalence and pharmacokinetic evaluation of two formulations of glimepiride 2 mg: a single-dose, randomized-sequence, open-label, two-way crossover study in healthy Chinese male volunteers. *Clinical therapeutics* **2010**, *32*, 986-995.
21. Kasichayanula, S.; Liu, X.; Shyu, W.; Zhang, W.; Pfister, M.; Griffen, S.; Li, T.; LaCreta, F.; Boulton, D. Lack of pharmacokinetic interaction between dapagliflozin, a novel sodium–glucose transporter 2 inhibitor, and metformin, pioglitazone, glimepiride or sitagliptin in healthy subjects. *Diabetes, Obesity and Metabolism* **2011**, *13*, 47-54.
22. Gu, N.; Kim, B.-H.; Rhim, H.; Chung, J.-Y.; Kim, J.-R.; Shin, H.-S.; Yoon, S.-H.; Cho, J.-Y.; Shin, S.-G.; Jang, I.-J. Comparison of the bioavailability and tolerability of fixed-dose combination glimepiride/metformin 2/500-mg tablets versus separate tablets: A single-dose, randomized-sequence, open-label, two-period crossover study in healthy Korean volunteers. *Clinical therapeutics* **2010**, *32*, 1408-1418.
23. Kim, B.-H.; Shin, K.-H.; Kim, J.; Lim, K.S.; Kim, K.-p.; Kim, J.-R.; Cho, J.-Y.; Shin, S.-G.; Jang, I.-J.; Yu, K.-S. Pharmacokinetic comparison of a new glimepiride 1-mg+ metformin 500-mg combination tablet formulation and a glimepiride 2-mg+ metformin 500-mg combination tablet formulation: a single-dose, randomized, open-label, two-period, two-way crossover study in healthy, fasting Korean male volunteers. *Clinical therapeutics* **2009**, *31*, 2755-2764.
24. Matsuki, M.; Matsuda, M.; Kohara, K.; Shimoda, M.; Kanda, Y.; Tawaramoto, K.; Shigetoh, M.; Kawasaki, F.; Kotani, K.; Kaku, K. Pharmacokinetics and pharmacodynamics of glimepiride in type 2 diabetic patients: compared effects of once-versus twice-daily dosing. *Endocrine journal* **2007**, *54*, 571-576.
25. Karim, A.; Zhao, Z.; Slater, M.; Bradford, D.; Schuster, J.; Laurent, A. Replicate study design in bioequivalency assessment, pros and cons: bioavailabilities of the antidiabetic drugs pioglitazone and glimepiride present in a fixed-dose combination formulation. *The Journal of Clinical Pharmacology* **2007**, *47*, 806-816.
26. Suzuki, K.; Yanagawa, T.; Shibasaki, T.; Kaniwa, N.; Hasegawa, R.; Tohkin, M. Effect of CYP2C9 genetic polymorphisms on the efficacy and pharmacokinetics of glimepiride in subjects with type 2 diabetes. *Diabetes research and clinical practice* **2006**, *72*, 148-154.
27. Jovanović, D.; Stojšić, D.; Zlatković, M.; Jović-Stošić, J.; Jovanović, M. Bioequivalence assessment of the two brands of glimepiride tablets. *Vojnosanitetski pregled* **2006**, *63*, 1015-1020.
28. Rabbaa-Khabbaz, L.; Abi Daoud, R.; Karam-Sarkis, D.; Atallah, C.; Zoghbi, A. A simple and sensitive method for determination of glimepiride in human serum by HPLC. *Journal of liquid chromatography & related technologies* **2005**, *28*, 3255-3263.

29. Pistos, C.; Koutsopoulou, M.; Panderi, I. Improved liquid chromatographic tandem mass spectrometric determination and pharmacokinetic study of glimepiride in human plasma. *Biomedical Chromatography* **2005**, *19*, 394-401.
30. Song, Y.-K.; Maeng, J.-E.; Hwang, H.-R.; Park, J.-S.; Kim, B.-C.; Kim, J.-K.; Kim, C.-K. Determination of glimepiride in human plasma using semi-microbore high performance liquid chromatography with column-switching. *Journal of Chromatography B* **2004**, *810*, 143-149.
31. Shukla, U.A.; Chi, E.M.; Lehr, K.-H. Glimepiride pharmacokinetics in obese versus non-obese diabetic patients. *Annals of Pharmacotherapy* **2004**, *38*, 30-35.
32. Salem, I.I.; Idrees, J.; Al Tamimi, J.I. Determination of glimepiride in human plasma by liquid chromatography–electrospray ionization tandem mass spectrometry. *Journal of chromatography B* **2004**, *799*, 103-109.
33. Niemi, M.; Cascorbi, I.; Timm, R.; Kroemer, H.K.; Neuvonen, P.J.; Kivistö, K.T. Glyburide and glimepiride pharmacokinetics in subjects with different CYP2C9 genotypes. *Clinical Pharmacology & Therapeutics* **2002**, *72*, 326-332.
34. Niemi, M.; Backman, J.T.; Neuvonen, M.; Laitila, J.; Neuvonen, P.J.; Kivistö, K.T. Effects of fluconazole and fluvoxamine on the pharmacokinetics and pharmacodynamics of glimepiride. *Clinical Pharmacology & Therapeutics* **2001**, *69*, 194-200.
35. Niemi, M.; Neuvonen, P.J.; Kivistö, K.T. Effect of gemfibrozil on the pharmacokinetics and pharmacodynamics of glimepiride. *Clinical Pharmacology & Therapeutics* **2001**, *70*, 439-445.
36. Niemi, M.; Kivistö, K.T.; Backman, J.T.; Neuvonen, P.J. Effect of rifampicin on the pharmacokinetics and pharmacodynamics of glimepiride. *British journal of clinical pharmacology* **2000**, *50*, 591-595.
37. Rosenkranz, B.; Profozic, V.; Metelko, Z.; Mrzljak, V.; Lange, C.; Malerczyk, V. Pharmacokinetics and safety of glimepiride at clinically effective doses in diabetic patients with renal impairment. *Diabetologia* **1996**, *39*, 1617-1624.
38. Malerczyk, V.; Badian, M.; Korn, A.; Lehr, K.-H.; Waldhäusl, W. Dose linearity assessment of glimepiride (Amaryl®) tablets in healthy volunteers. *Drug Metabolism and Drug Interactions* **1994**, *11*, 341-357.
39. Badian, M.; Korn, A.; Lehr, K.-H.; Malerczyk, V.; Waldhäusl, W. Absolute bioavailability of glimepiride (Amaryl®) after oral administration. *Drug Metabolism and Drug Interactions* **1994**, *11*, 331-340.
40. Lehr, K.; Damm, P. Simultaneous determination of the sulphonylurea glimepiride and its metabolites in human serum and urine by high-performance liquid chromatography after pre-column derivatization. *Journal of Chromatography B: Biomedical Sciences and Applications* **1990**, *526*, 497-505.
